# The effect of ginsenosides on liver injury in preclinical studies: a systematic review and meta-analysis

**DOI:** 10.3389/fphar.2023.1184774

**Published:** 2023-05-11

**Authors:** Xing-Bo Bian, Peng-Cheng Yu, Xiao-Hang Yang, Liu Han, Qi-Yao Wang, Li Zhang, Lian-Xue Zhang, Xin Sun

**Affiliations:** ^1^ College of Pharmacy, Jilin Medical University, Jilin, Jilin, China; ^2^ College of Pharmacy, Heilongjiang University of Chinese Medicine, Harbin, Heilongjiang, China; ^3^ College of Chinese Medicinal Materials, Jilin Agriculture University, Changchun, Jilin, China

**Keywords:** ginsenosides, liver injury, animal models, meta-analysis, systematic review

## Abstract

**Background:** Liver injury is a severe liver lesion caused by various etiologies and is one of the main areas of medical research. *Panax ginseng* C.A. Meyer has traditionally been used as medicine to treat diseases and regulate body functions. Ginsenosides are the main active components of ginseng, and their effects on liver injury have been extensively reported.

**Methods:** Preclinical studies meeting the inclusion criteria were retrieved from PubMed, Web of Science, Embase, China National Knowledge Infrastructure (CNKI), and Wan Fang Data Knowledge Service Platforms. The Stata 17.0 was used to perform the meta-analysis, meta-regression, and subgroup analysis.

**Results:** This meta-analysis included ginsenosides Rb1, Rg1, Rg3, and compound K (CK), in 43 articles. The overall results showed that multiple ginsenosides significantly reduced alanine aminotransferase (ALT) and aspartate aminotransferase (AST), affected oxidative stress-related indicators, such as superoxide dismutase (SOD), malondialdehyde (MDA), glutathione (GSH), glutathione peroxidase (GSH-Px), and catalase (CAT), and reduced levels of inflammatory factor, such as factor-α (TNF-α), interleukin-1β (IL-1β), interleukin-6 (IL-6). Additionally, there was a large amount of heterogeneity in the meta-analysis results. Our predefined subgroup analysis shows that the animal species, the type of liver injury model, the duration of treatment, and the administration route may be the sources of some of the heterogeneity.

**Conclusion:** In a word, ginsenosides have good efficacy against liver injury, and their potential mechanisms of action target antioxidant, anti-inflammatory and apoptotic-related pathways. However, the overall methodological quality of our current included studies was low, and more high-quality studies are needed to confirm their effects and mechanisms further.

## 1 Introduction

The liver, which is the largest digestive and metabolic organ in the human body, plays an essential role in metabolizing vitamins, hormones, bile, and toxins (Protzer et al., 2012; Lee and Takebe, 2022). It is also the body’s most crucial detoxification organ, responsible for biotransforming various non-nutrient substances, such as drugs, poisons, and toxic metabolites. Typically, the liver can metabolize or transform these substances into less toxic substances and then excrete them from the body. However, liver tissue is vulnerable to damage by various toxic substances or drugs (Sun et al., 2022). Liver injury refers to the swelling, degeneration, necrosis or apoptosis of liver cells in varying degrees under the action of some physical and chemical factors or external environment (Malhi et al., 2010). Moreover, liver injury is a fundamental pathological state common to all kinds of liver diseases during the early onset stage. Over time, after a series of complex pathological changes, liver injury can progress to liver fibrosis, cirrhosis, liver cancer and liver failure (Duarte et al., 2015; Chen et al., 2022).

Several factors can lead to liver injury, such as viral infection, exposure to toxic substances, high alcohol consumption, and the use of certain drugs. Environmental pollution, the decline of air quality, and other related factors increase people’s exposure to hepatotoxic chemical, which have resulted in an increasing incidence such as hepatitis, cirrhosis, fatty liver and liver cancer caused by chemical liver injury (Asrani et al., 2019). Additionally, long-term excessive alcohol consumption poses a hidden threat to human health, leading to liver injury (Lucey et al., 2020). Certain drugs or their metabolites can also cause liver injury, which can be predictable, such as acetaminophen (APAP), or unpredictable (Leise et al., 2014). Moreover, immune liver injuries and liver ischemia-reperfusion injuries are also common types of liver damage (Adams et al., 2010; Konishi and Lentsch, 2017).

Ginseng (*Panax ginseng* C.A. Meyer) is a highly valuable medicinal plants, particularly in China, Korea, and Japan (Li et al., 2022). It has been globally utilized to treat a variety of diseases (Fan et al., 2020; Kim and Cho, 2021). Currently, food products that incorporate ginseng are being developed at a gradual pace (Guo et al., 2021). Ginsenosides, the primary bioactive components of ginseng, are responsible for its various pharmacological effects. More than 40 ginsenosides components have been identified and isolated (Yu et al., 2019). In general, ginsenosides are classified into four types: 1) protopanaxadiol (PPD) type, such as ginsenoside Ra1-3, Rb1-2, Rd, and Rg3; 2) protopanaxatriol (PPT) type, such as ginsenoside Re, Rf, Rg1-2, and Rh1; 3) oleanolic acid type, such as ginsenoside Ro; 4) ocotillol type such as ginsenoside F11, RT2, and RT4 (Liu et al., 2017; Kim and Cho, 2021). Numerous studies have confirmed the pharmacological properties of ginsenosides, such as anti-tumor, neuroprotective, anti-inflammatory effect, among others (Sng et al., 2022). Furthermore, various ginsenosides are capable of enhancing and safeguarding liver function and health. In previous studies, different ginsenosides have been investigated to prove their protective or therapeutic effects on multiple types of liver injury, and their corresponding mechanisms of action were proposed (Huu Tung et al., 2012).

The study of the effects of ginsenosides on liver injury has primarily been conducted using animal and cell models, which have demonstrated the significant potential of ginsenosides. These studies have investigated the mechanism of action of specific ginsenosides on liver injury using diverse models. Nevertheless, there has been no meta-analysis to date based on preclinical studies that synthesize the evidence on the effects of ginsenosides for treating liver injury. Therefore, this study performed a rigorous and comprehensive systematic review and meta-analysis to evaluate the effects of ginsenosides on various types of liver injury. The findings provide a foundation and a point of reference for the clinical application of ginsenosides.

## 2 Materials and methods

### 2.1 Search strategy

To investigate the effects of ginsenosides on liver injury, two trained researchers independently searched articles published up to May 2022 from five databases, including PubMed (https://pubmed.ncbi.nlm.nih.gov/), Web of Science (http://apps.webofknowledge.com/), Embase (https://www.embase.com), China National Knowledge Infrastructure (CNKI) (https://www.cnki.net/) and Wan Fang (https://www.wanfangdata.com.cn). The search terms such as: “ginsenosides,” “ginseng saponin,” “liver injury,” “liver damage,” “hepatic injury,” and “hepatotoxicity.” The search used medical subject headings (MeSH) and free-text words and was modified to suit each database. Furthermore, there were no restrictions on language or year of publication. The search strategy of the PubMed database is shown in the supplementary material.

### 2.2 Inclusion and exclusion criteria

Included studies were screened based on preformed inclusion and exclusion criteria. The inclusion criteria were: 1) peer-reviewed original articles; 2) research on animal models of liver injury; 3) treatment with ginsenoside; 4) the control group was untreated-controlled or vehicle-controlled; 5) outcomes include changes in necessary blood or liver tissue biomarkers or pathological changes, or other relevant mechanisms.

The exclusion criteria were: 1) reviews or case reports; 2) clinical trials or only *in vitro* studies; 3) not the liver injury models; 4) treatment without ginsenosides or in combination with other interventions; 5) lack of control group; 6) the control group was treated with any other therapeutic drugs.

### 2.3 Data extraction and management

EndNote software (version X9) was used to manage the retrieved literature and delete duplicate literature. Then, two independent researchers assessed the quality of the literature and performed data collection separately based on the inclusion and exclusion criteria. The specific processing process and data extraction are as follows: 1) titles and abstracts are surveyed for preliminary screening; 2) full texts are reviewed to assess their suitability for meta-analysis; 3) information extracted from each study, including the publication information (author name and publication year), animal information (animal species, sex, number of animals in each group, and modeling methods), intervention information (types of ginsenosides, vendor, dosage form, dose, duration of treatment, and administration route), and outcome assessment (specific blood and liver tissue biomarkers and mechanisms). For a graphical presentation of the results, GetData software (version 2.26) was used to extract the data. Any discrepancies were resolved through discussion by the researchers.

### 2.4 Risk of bias assessment

To determine the methodological quality of the included studies, two researchers conducted independent evaluations of these studies using SYRCLE’s bias risk tool (Hooijmans et al., 2014). In this section, six types of bias are evaluated, including selection bias, performance bias, detection bias, attrition bias, reporting bias, and other biases. By judgment, each entry is identified as one of three outcomes, including low risk, high risk, and unclear risk. One point is awarded for each item judged to be low risk, and the total score of each study was 10 points. Any discrepancies were resolved through discussion by the researchers.

### 2.5 Data synthesis and analysis

When standard errors (SE) was used in the study to report the results, we used the following formula to calculate the standard deviations (SD) values:

$$SD=SE*\sqrt{N}$$

N is the number of samples.

All meta-analyses were performed using Stata software (version 17.0). Here, in view of the differences in animal experimental design, we use the random-effects model to pool the effect sizes. As the major outcomes were continuous variables, the effect sizes were expressed using the standard mean difference (SMD) and 95% confidence interval (95% CI). *p* < 0.05 was considered statistically significant. And I^2^ and Cochran’s *Q* statistics were used to assess the heterogeneity of included studies. I^2^ > 50%, *P*_Q-test_ < 0.1 is considered to have significant heterogeneity. Meta-regression analysis was performed using Stata software (version 17.0) to assess potential vital variables that could have a significant impact on heterogeneity. The variables we considered included the animal species, the type of liver injury model, the duration of treatment (once; ≤7 days; >7 days and < 30 days; ≥30 days), and the administration route. Then, when meta-regression results showed that a variable had a significant effect on inter-study heterogeneity, a subgroup analysis was performed on that variable. Sensitivity analysis was also performed to assess the influence of individual studies on SMD and 95% CI by excluding each study, in turn, for each of the parameters considered. Finally, Stata software (version 17.0) was used to evaluate potential publication bias with Egger’s linear regression test.

## 3 Results

### 3.1 Description of included studies

**FIGURE 1:**
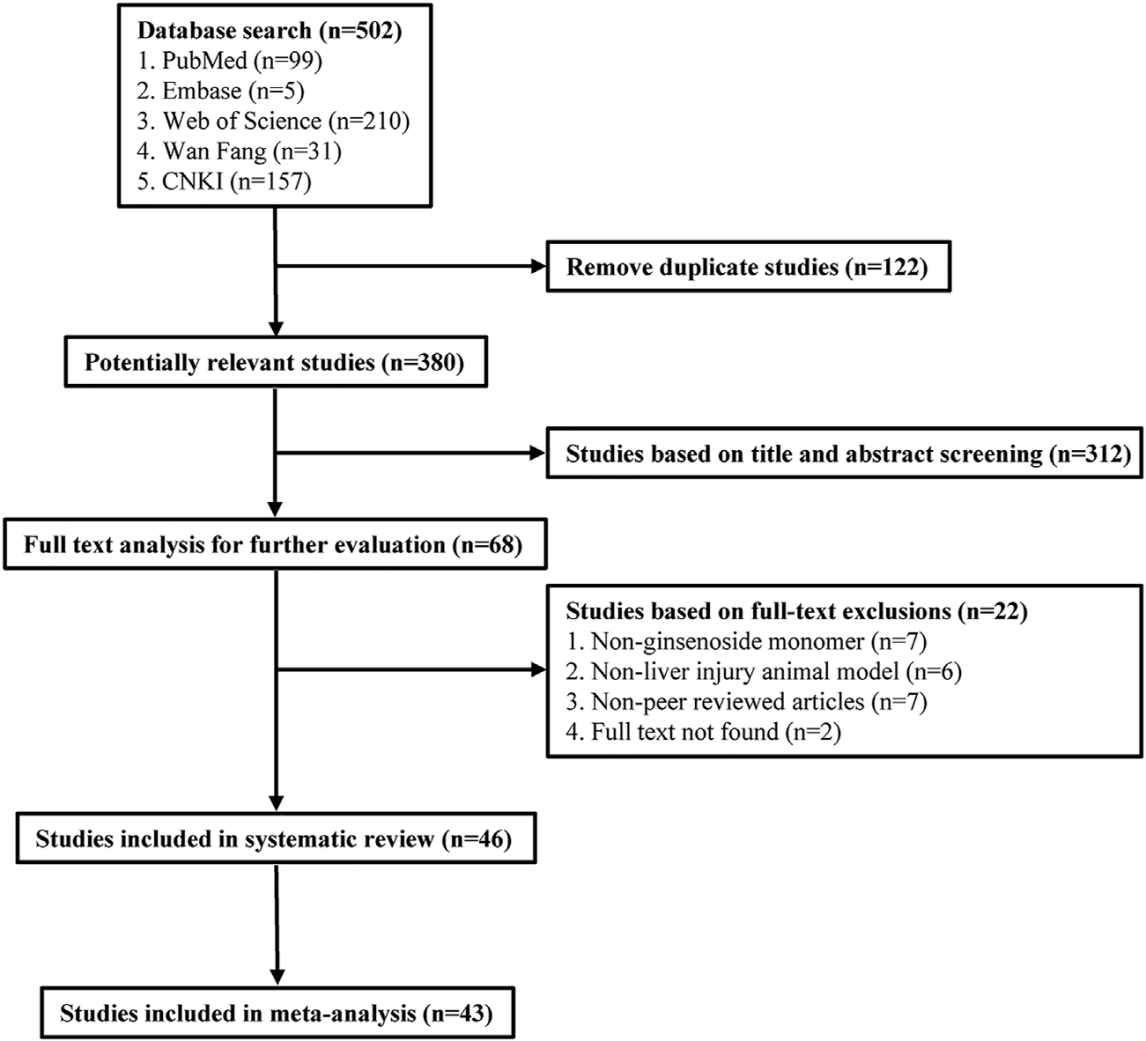
Flowchart for selection of studies.

A total of 502 publications were retrieved from five databases (99 from PubMed, 5 from Embase, 210 from Web of Science, 31 from Wan Fang, and 157 from CNKI). After eliminating duplicates, 380 publications remained. Out of these, 312 publications were excluded as they did not meet our inclusion criteria during the initial screening of the titles and abstracts. Further, 22 studies were excluded after the full-text screening, and 46 studies were ultimately included in the systematic review. Finally, 46 studies were included in the systematic review, of which 43 publications were included in the meta-analysis. Figure 1 depicts the details of the study selection process.

### 3.2 Characteristics of included studies

The characteristics of the 46 studies that were included in this research have been summarized in Supplementary Table S1. The publications of these studies range from 2005 to 2022, where 10 of them are in the Chinese language and 36 in English. In addition, five of these studies analyzed two ginsenosides concurrently, while the remaining studies concentrated on examining a single ginsenoside.

#### 3.2.1 Animals and liver injury models

In the studies included in this review, rats, mice, and zebrafish were used in liver injury research. Sprague Dawley (SD) rats were used in 9 studies (Liu et al., 2014; Li, 2015; Tian et al., 2017; Li et al., 2018; Zhang Y. et al., 2019; Chen et al., 2020; Lin et al., 2020; Yan et al., 2020; Zhou et al., 2020), Wistar rats were used in 4 studies (Zhang et al., 2006; Kang et al., 2007; Wang et al., 2008; Wang et al., 2022), and one study (Yao, 2016) did not identify rat strains. Of the studies using mice, Kunming mice were used in 5 studies (Yang et al., 2015; Qi, 2016; Xin et al., 2016; Qi et al., 2017; Bi et al., 2021), ICR mice were used in 10 studies (LEE et al., 2005a; Lee H. U. et al., 2005; Li et al., 2011; Wang et al., 2017; Lu et al., 2018; Zhou et al., 2018; Qu et al., 2019; Ren et al., 2019; Li et al., 2020; Qu et al., 2021), C57BL/6 mice were used in 15 studies (Tao et al., 2014; Zhang et al., 2015; Gao et al., 2016; Gao et al., 2017; Ning et al., 2018a;b; Ning et al., 2018c; Xiao et al., 2018; Kim et al., 2020; Gao et al., 2021; Liu et al., 2021; Wu et al., 2021; Yang et al., 2021; Zhang et al., 2021; Zhao et al., 2021), and db/db mice were used in one study (Jiang et al., 2021). Four studies did not report the sex of mice or rats (Zhang et al., 2006; Li, 2015; Yao, 2016; Chen et al., 2020), two used female mice (Bi et al., 2021; Yang et al., 2021), and the rest used male mice or rats. There was also a study using zebrafish for liver injury research (Lai et al., 2021). In this study, the researchers used wild-type, lfabp10α: EGFP, and MPO: EGFP zebrafish.

Our review encompasses various multiple animal models of liver injury (Supplementary Table S1) given the different types of liver injuries. Chemical liver injury studies involving ginsenosides are the most reported, and CCl_4_-induced liver injury as the most commonly used model. Other studies have explored the efficacy of ginsenosides using LPS, D-Gal, or *t*-BHP-induced liver injury. Drug-induced liver injury is also an important type, and we included 7 studies of APAP-induced liver injury models, and one study each for cisplatin-induced liver injury model and sodium valproate-induced liver injury model. Seven studies investigated alcohol-induced liver injury, while one study focused on Con A-induced the immune liver injury. Additionally, we examined 6 studies on ginsenosides’ effects on diabetic liver injury, with 5 studies establish the type Ⅱ diabetic liver injury model using high sugar and high-fat feeding and streptozocin, and one study on spontaneous diabetic liver injury in db/db mice. Six studies analyzed the effects of ginsenosides on liver ischemia-reperfusion injury, and another study analyzed liver ischemia-reperfusion injury in diabetic rats. Finally, we reviewed a model of septic liver injury that was constructed through caecal ligation and puncture.

#### 3.2.2 Ginsenosides

Among the included studies, 11 species of ginsenosides were reported to affect liver injury. Among them, there were 22 studies on ginsenoside Rg1, with the highest number (Tao et al., 2014; Li, 2015; Yang et al., 2015; Zhang et al., 2015; Gao et al., 2016; Qi, 2016; Xin et al., 2016; Yao, 2016; Gao et al., 2017; Qi et al., 2017; Tian et al., 2017; Lu et al., 2018; Ning et al., 2018a; Ning et al., 2018b; Ning et al., 2018c; Xiao et al., 2018;Chen et al., 2020; Lin et al., 2020;Bi et al., 2021; Yang et al., 2021; Zhang et al., 2021; Zhao et al., 2021). This was followed by ginsenoside Rg3 (LEE et al., 2005a; Kang et al., 2007; Zhou et al., 2018; Li et al., 2020; Gao et al., 2021; Jiang et al., 2021; Wu et al., 2021), Rb1 (Lee H. U. et al., 2005; Wang et al., 2008; Liu et al., 2014; Lu et al., 2018; Ren et al., 2019; Lai et al., 2021; Liu et al., 2021), CK (Lee H. U. et al., 2005; Zhang et al., 2006; Li et al., 2011; Li et al., 2018; Zhang Y. et al., 2019; Yan et al., 2020; Zhou et al., 2020), Rk3 (Qu et al., 2019; Qu et al., 2021), Rh1 (Li et al., 2018; Bi et al., 2021), Rh2 (LEE et al., 2005a), Rg5 (Wang et al., 2017), F2 (Kim et al., 2020), and Mc1 (Wang et al., 2022). Four studies highlighted the epimer of ginsenoside Rg3 [20(*S*) or 20(*R*)] (LEE et al., 2005a; Kang et al., 2007; Zhou et al., 2018; Li et al., 2020), and 1 study highlighted the epimer of Rh2 [20(*S*)] (LEE et al., 2005a).

The studies involve both the pretreatment and post-treatment application of ginsenosides. In the studies of ginsenoside pretreatment, the protective effect of ginsenoside on the liver was emphasized. Ginsenosides are mainly administered by intragastric and intraperitoneal injection. Additionally, vein injection was conducted in three studies (Liu et al., 2014; Lin et al., 2020; Zhang et al., 2021).

The administration of ginsenosides varies in duration depending on the model of liver injury (Supplementary Table S1). The shortest duration of ginsenoside administration was observed in the liver ischemia-reperfusion injury model, where pretreatment with ginsenosides occurred only once. Of the 17 studies reviewed, dosing durations of 7 days or less were reported. In 10 of the studies, the duration of dosing was 4 weeks or less. In 8 studies, the dosing duration exceeded 4 weeks, with the longest duration being 8 weeks. Some studies included two durations in their experimental design. For instance, one study reported of 5 or 8 days, while two other studies reported durations of either once or 3 days.

### 3.3 Quality of the included studies

Bias risk assessment was performed on 46 included studies based on 10 items from SYRCLE’s risk of bias tool (Hooijmans et al., 2014). The risk of bias for each included study is summarized in Supplementary Table S1. The risk of bias scores for all studies were in the range of 2-3, and details of some studies were underreported. As a result, several of the studies were judged to have an “unclear risk of bias.” None of the 46 studies provided a detailed description of the method of allocation sequence generation and baseline characteristics similarity between groups. In addition, no studies have explicitly addressed whether the allocation to the different groups was adequately concealed. A total of 26 studies detailed the living conditions of the animals, we assumed that the animals were housed randomly during the experiments. For the blinding of caregivers or investigators, randomization, or blinding of outcome assessment, none of the included studies provided adequate information. All included studies reported all animals or adequately processed incomplete outcome data. At the same time, all expected outcomes were comprehensively reported in all included studies. Finally, other biases in these studies could not be accurately assessed.

### 3.4 Efficacy of ginsenoside Rg1 in liver injury

#### 3.4.1 Alanine aminotransferase (ALT) and aspartate aminotransferase (AST)

**FIGURE 2:**
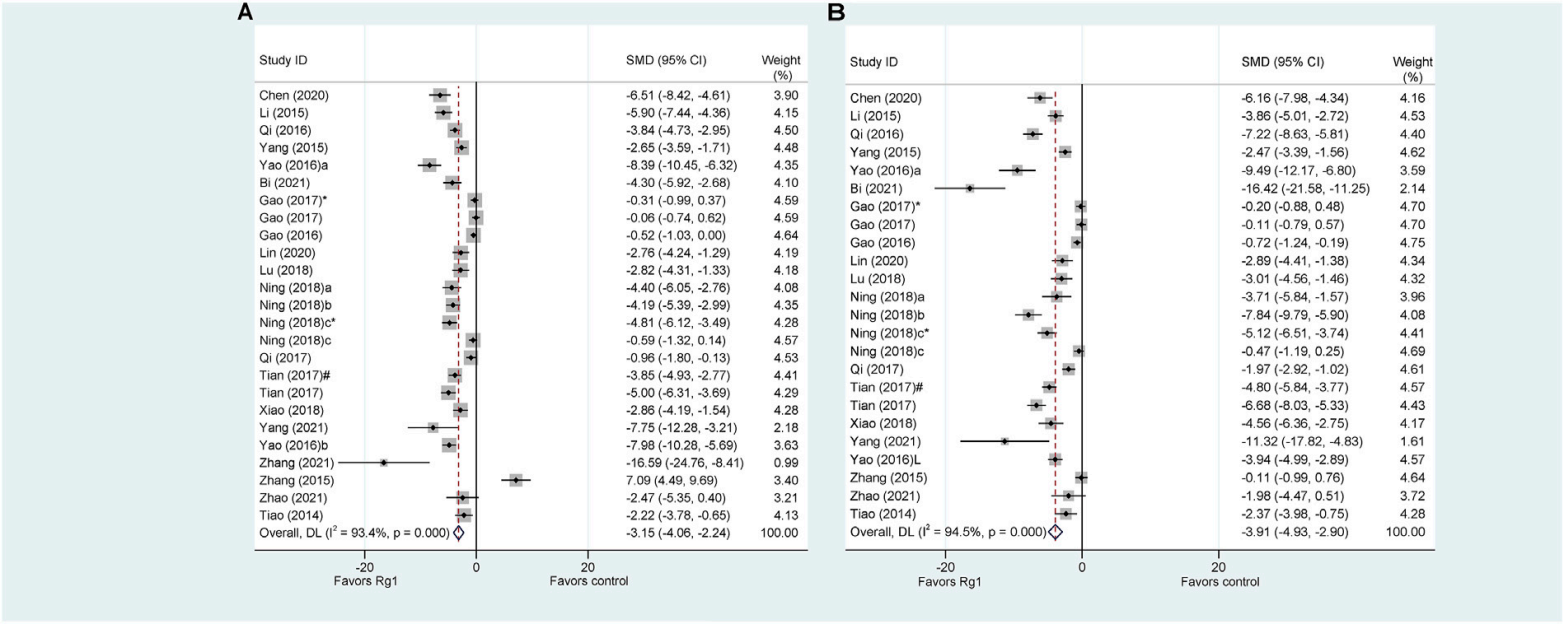
Standard mean differences estimates for the effects of ginsenoside Rg1 on **(A)** ALT and **(B)** AST.

Serum ALT and AST are the most sensitive biomarkers for evaluating liver injury (Zhang et al., 2016). ALT was used as the outcome measure in all 22 articles that investigated the effect of Rg1 on liver injury. The results of the meta-analysis showed that Rg1 could reduce ALT levels compared with the control group (SMD = −3.151, 95% CI: −4.063 to −2.238, *p* = 0.000; heterogeneity: I^2^ = 93.4%, *P*_Q-test_ = 0.000) (Figure 2A). Given the heterogeneity of the studies and the number of included studies, a meta-regression analysis was conducted. The findings indicated that neither “Animal species,” “Liver injury model,” “Administration route,” “Prophylactic or therapeutic,” nor “Duration of treatment” was the source of heterogeneity across studies (Table 1).

**TABLE 1:** The results of meta-regression analysis of the effects of ginsenoside Rg1 on ALT and AST.Results of subgroup

Parameter	Variable	Coefficient	t	*p*-value	95% CI
ALT	Animal species	1.302599	0.50	0.624	−4.174009 to 6.779207
	Liver injury model	0.8013307	1.35	0.193	−0.441466 to 2.044127
	Administration route	0.2487962	0.3	0.769	−1.498301 to 1.995893
	Prophylactic or therapeutic	0.5532367	0.33	0.745	−2.953059 to 4.059533
	Duration of treatment	−0.9702481	−0.9	0.379	−3.225855 to 1.285359
AST	Animal species	0.3542071	0.13	0.898	−5.347071 to 6.055485
	Liver injury model	0.3884657	0.64	0.53	−0.8875267 to 1.664458
	Administration route	−0.296225	−0.34	0.738	−2.12884 to 1.53639
	Prophylactic or therapeutic	−2.146682	−1.21	0.244	−5.888305 to 1.594941
	Duration of treatment	1.186682	1.37	0.304	−1.171683 to 3.545046

Only one article on Rg1 did not report AST levels. The results of the analysis indicate that Rg1 treatment significantly reduced AST levels in comparison to the control group (SMD = −3.91, 95% CI: −4.93 to −2.90, *p* = 0.000; heterogeneity: I^2^ = 94.5%, *P*_Q-test_ = 0.000) (Figure 2B). The meta-regression analysis of the AST was conducted and yielded results similar to those of the ALT. “Animal species,” “Liver injury model,” “Administration route,” “Prophylactic or therapeutic,” and “Duration of treatment” were not identified as source of study heterogeneity (Table 2).

**TABLE 2:** Results of subgroup analysis of the effects of ginsenoside Rg1 on oxidative stress indicators.

Indicators	Subgroup		No. of studies	SMD [95% CI]	*p*-value	I^2^	*p*-value for heterogeneity
SOD	Species	rats	3	2.474 [1.491 to 3.456]	0.000	71.60%	0.029
		mice	8	3.581 [2.335 to 4.827]	0.000	90.0%	0.000
	Model	liver injury in type 2 diabetic rats	3	2.474 [1.491 to 3.456]	0.000	71.6%	0.029
		chemical liver injury	6	3.778 [2.150 to 5.405]	0.000	92.1%	0.000
		drug-induced liver injsury	2	3.021 [0.866 to 5.176]	0.06	82.4%	0.017
	Administration	by intragastric	9	2.342 [3.920 to 82.52]	0.000	80.4%	0.000
		by intraperitoneal injection	2	3.976 [22121.630 to 9.581]	0.000	97.1%	0.000
	Prophylactic or Therapeutic	therapeutic	4	2.134 [1.202 to 3.067]	0.000	78.4%	0.003
		prophylactic	7	3.944 [2.714 to 5.174]	0.000	86.4%	0.000
	Duration	≥30 days	4	2.134 [1.202 to 3.067]	0.000	78.4%	0.003
		≤7 days	7	3.944 [2.714 to 5.174]	0.000	86.4%	0.000
MDA	Species	rats	2	−2.637 [−4.288 to −0.985]	0.002	82.4%	0.017
		mice	12	−3.228 [−4.175 to −2.281]	0.000	88.9%	0.000
	Model	liver injury in type 2 diabetic rats	4	−3.070 [−3.990 to −2.149]	0.000	70.4%	0.017
		chemical liver injury	5	−2.927 [−3.771 to −2.083]	0.000	67.9%	0.014
		drug-induced liver injury	4	−3.556 [−5.826 to −1.285]	0.002	94.7%	0.000
		alcoholic liver injury	1	——	——	——	——
	Administration	by intragastric	11	−3.161 [−4.114 to −2.208]	0.000	89.6%	0.000
		by intraperitoneal injection	3	−2.938 [−4.630 to −1.247]	0.001	76%	0.015
	Prophylactic or Therapeutic	therapeutic	6	−2.444 [−3.640 to −1.248]	0.000	87.3%	0.000
		prophylactic	8	−3.615 [−4.712 to −2.517]	0.000	87%	0.000
	Duration	≥30 days	5	−3.575 [−5.162 to −1.988]	0.000	89%	0.000
		≤7 days	8	−3.176 [−4.272 to −2.080]	0.000	87.4%	0.000
		>7 days and < 30 days	1	——	——	——	——
GSH	Model	drug-induced liver injury	4	2.505 [0.324 to 4.685]	0.024	94.7%	0.000
		chemical liver injury	3	5.050 [0.319 to 9.780]	0.036	96.3%	0.000
	Administration	by intragastric	5	3.061 [0.937 to 5.184]	0.005	94.6%	0.000
		by intraperitoneal injection	2	4.896 [−2.598 to 12.391]	0.2	97.7%	0.000
	Prophylactic or Therapeutic	therapeutic	2	0.869 [0.342 to 1.397]	0.001	0	0.334
		prophylactic	5	4.738 [1.520 to 7.957]	0.004	96.1%	0.000
	Duration	≥30 days	1	——	——	——	——
		≤7 days	5	4.821 [1.840 to 7.801]	0.002	95.4%	0.000
		>7 days and < 30 days	1	——	——	——	——
GSH-Px	Species	rats	1	——	——	——	——
		mice	4	1.063 [−0.358 to 2.484]	0.143	89.8%	0.000
	Model	liver injury in type 2 diabetic rats	1	——	——	——	——
		drug-induced liver injury	2	−0.174 [−0.771 to 0.423]	0.567	34.7%	0.216
		chemical liver injury	1	——	——	——	——
		alcoholic liver injury	1	——	——	——	——
	Administration	by intragastric	3	0.194 [−0.612 to 1.000]	0.637	74.2%	0.021
		by intraperitoneal injection	2	3.249 [−0.199 to 6.697]	0.065	77%	0.037
	Prophylactic or Therapeutic	therapeutic	4	1.324 [−0.120 to 2.769]	0.072	89.3%	0.000
		prophylactic	1	——	——	——	——
	Duration	≥30 days	2	1.401 [0.572 to 2.230]	0.001	57.9%	0.123
		≤7 days	1	——	——	——	——
		>7 days and < 30 days	2	2.518 [−2.642 to 7.679]	0.169	89.5%	0.002
MPO	Model	drug-induced liver injury	2	−0.004 [−1.086 to 1.078]	0.994	79.9%	0.026
		chemical liver injury	1	——	——	——	——
	Prophylactic or Therapeutic	therapeutic	2	−3.849 [−10.454 to 2.756]	0.253	97.2%	0.000
		prophylactic	1	——	——	——	——
	Duration	≤7 days	2	−3.308 [−10.995 to 4.379]	0.399	97.9%	0.000
		>7 days and < 30 days	1	——	——	——	——
CAT	Species	rats	1	——	——	——	——
		mice	2	5.658 [−0.003 to 11.319]	0.05	95.3%	0.000
	Prophylactic or Therapeutic	therapeutic	1	——	——	——	——
		prophylactic	2	5.658 [−0.003 to 11.319]	0.05	95.3%	0.000
	Duration	≥30 days	1	——	——	——	——
		≤7 days	2	5.658 [−0.003 to 11.319]	0.05	95.3%	0.000
T-AOC	Species	rats	1	——	——	——	——
		mice	2	0.107 [−2.674 to 2.888]	0.94	96.6%	0.000
	Model	liver injury in type 2 diabetic rats	1	——	——	——	——
		drug-induced liver injury	2	0.107 [−2.674 to 2.888]	0.94	96.6%	0.000
	Prophylactic or Therapeutic	therapeutic	2	0.074 [−2.655 to 2.802]	0.958	96%	0.000
		prophylactic	1	——	——	——	——

#### 3.4.2 Indicators of oxidative stress

**FIGURE 3:**
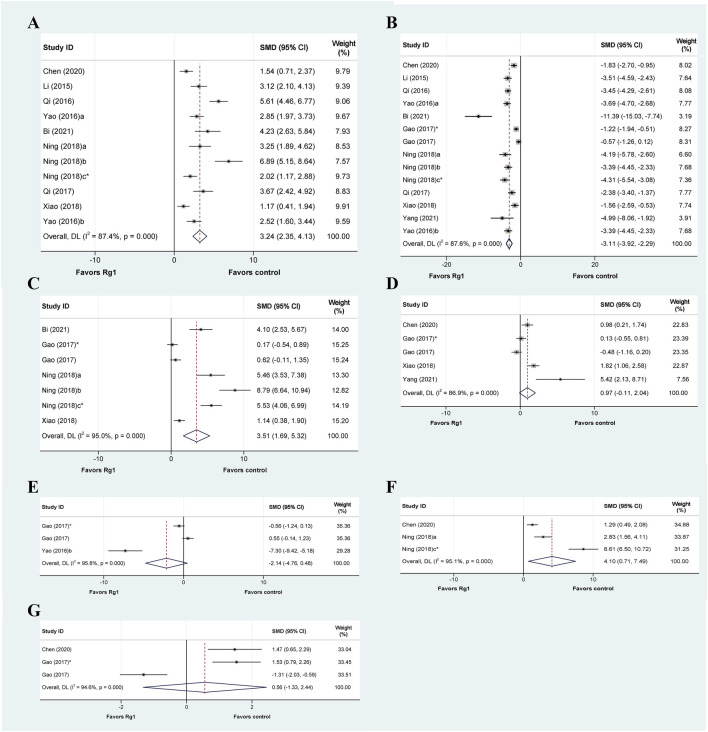
Standard mean differences estimates for the effects of ginsenoside Rg1 on **(A)** SOD, **(B)** MDA, **(C)** GSH, **(D)** GSH-Px, **(E)** MPO, **(F)** CAT, and **(G)** T-AOC.

Oxidative stress is believed to be a crucial factor in the development of liver injury (Reiter et al., 2000; Zhang X. et al., 2019). A total of 13 articles explored the oxidative stress-related mechanisms of Rg1 action on liver injury and analyzed the relevant indicators. Among them, eleven studies reported changes in superoxide dismutase (SOD), and the results showed that the Rg1 treatment significantly increased SOD levels significantly. These results were also confirmed by meta-analysis, with significant heterogeneity (SMD = 3.24, 95% CI: 2.35 to 4.13, *p* = 0.000; heterogeneity: I^2^ = 87.4%, *P*_Q-test_ = 0.000) (Figure 3A). Furthermore, we conducted predefined subgroup analyses to evaluate the effect of study characteristics on the Rg1 effect on SOD levels (Table 2), following the parameters set in the above meta-regression analysis. The subgroup analysis of SOD demonstrated substantial heterogeneity among the subgroups. Moreover, in the subgroup analysis of the “Liver injury model,” the result of drug-induced liver injury was not statistically significant (*p* = 0.06) (Table 2).

Thirteen articles encompassing fourteen studies examined the effects of Rg1 on malondialdehyde (MDA) levels in liver injury. The results indicated that Rg1 was successful in reducing MDA levels. The meta-analysis of these studies showed that Rg1 was effective (SMD = −3.11, 95% CI: −3.92 to −2.29, *p* = 0.000); however, significant heterogeneity was observed (I^2^ = 95.0%, *P*_Q_-test = 0.000) (Figure 3B). Additionally, subgroup analysis showed significant statistical differences across all subgroups; however, substantial heterogeneity persisted (Table 2).

Seven studies from five articles reported the effect of Rg1 on glutathione (GSH), and it could be observed that Rg1 could effectively reduce the level of GSH in liver injury (SMD = 3.51, 95% CI: 1.69 to 5.32, *p* = 0.000; heterogeneity: I^2^ = 95.0%, *P*_Q-test_ = 0.000) (Figure 3C). In the subgroup analysis of Administration, the result of intraperitoneal injection was not significant (*p* = 0.2). In the results from the subgroup analysis for “prophylactic or therapeutic,” the heterogeneity in the therapeutic was significantly reduced (I^2^ = 0, *P*_Q-test_ = 0.334) (Table 2).

Five studies were conducted to examine the effect of Rg1 treatment on glutathione peroxidase (GSH-Px) levels compared to a control group. The data suggest that Rg1 is not able to increase GSH-Px levels significantly (SMD = 0.97, 95% CI: −0.11 to 2.04, *p* = 0.078; heterogeneity: I^2^ = 86.9%, *P*_Q-test_ = 0.000) (Figure 3D). Notably, the predefined subgroup analyses did not yield statistically significant differences, with the exception of the subgroup analysis of “Duration of treatment” where the difference was significant for treatments ≥30 days (*p* = 0.001). In addition, our findings show that drug-induced liver injury heterogeneity was significantly reduced in the subgroup analysis of the “Liver injury model” (I^2^ = 34.7%, *P*_Q-test_ = 0.216) (Table 2).

Three studies analyzed myeloperoxidase (MPO), and based on the data, we found that Rg1 may not have a significant effect on MPO levels (SMD = −2.14, 95% CI: −4.76 to 0.48, *p* = 0.109). The heterogeneity test indicated the presence of heterogeneity (I^2^ = 95.8%, *P*_Q-test_ = 0.000) (Figure 3E). In the three subgroup analyses that could be performed, the results were also not significant, and the heterogeneity remained high (Table 2).

Three studies have investigated the effect of Rg1 treatment on catalase (CAT) activity. The analysis showed that Rg1 treatment significantly increased CAT levels and that heterogeneity was present (SMD = 4.10, 95% CI: −0.71 to 7.49, *p* = 0.000; heterogeneity: I^2^ = 95.1%, *P*_Q-test_ = 0.000) (Figure 3F). The same set of studies was included in all three subgroup analyses conducted on CAT. Consequently, the outcomes were consistent (SMD = 5.658, 95% CI: −0.003 to 11.319, *p* = 0.000; heterogeneity: I^2^ = 95.3%, *P*_Q-test_ = 0.000) (Table 2).

Three studies analyzed the effect of Rg1 treatment on total antioxidant capacity (T-AOC) in liver injury. The results of the meta-analysis showed that the effect of Rg1 on T-AOC was not significant, and the heterogeneity test indicated the presence of heterogeneity (SMD = 0.56, 95% CI: −1.33 to 2.44, *p* = 0.562; heterogeneity: I^2^ = 94.6%, *P*_Q-test_ = 0.000) (Figure 3G). We further conducted three subgroup analyses, but the outcomes still showed high heterogeneity (Table 2).

Furthermore, there is only one study that reported the effect of Rg1 treatment on the levels of reactive oxygen species (ROS). The results showed that Rg1 treatment resulted in a significant reduction in ROS levels (*p* < 0.05).

#### 3.4.3 Indicators of inflammatory response

**FIGURE 4:**
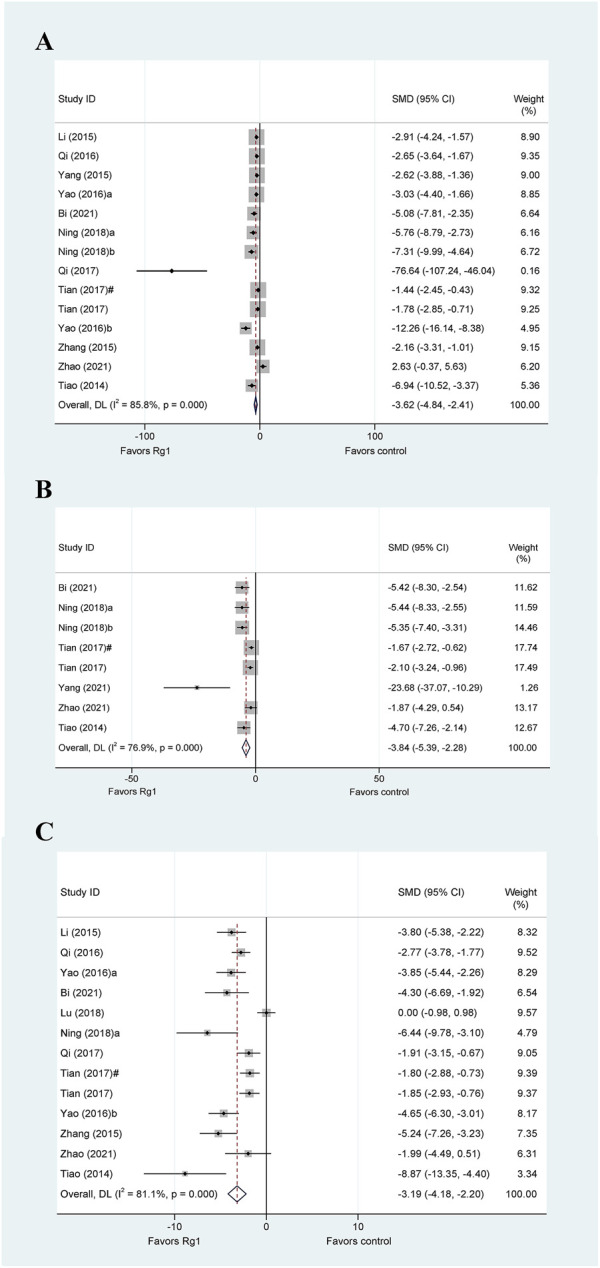
Standard mean differences estimates for the effects of ginsenoside Rg1 on **(A)** TNF-α, **(B)** IL-1β, and **(C)** IL-6.

Proinflammatory responses are crucial in various types of liver injury. Various inflammation-related cytokines, including tumor necrosis factor-α (TNF-α), interleukin-1β (IL-1β), and interleukin-6 (IL-6), play a crucial role in regulating the development of liver injury, and their blockade can alleviate injury. The TNF-α data in liver injury were reported in fourteen studies. The meta-analysis findings indicated that Rg1 reduced TNF-α levels in liver injury (SMD = −3.62, 95% CI: −4.84 to −2.41, *p* = 0.000), there was heterogeneity in the results (I^2^ = 85.8%, *P*_Q-test_ = 0.000) (Figure 4A). We performed five predefined subgroup analyses for TNF-α. In the subgroup analysis of “Animal species,” the heterogeneity level of rats was significantly decreased (I^2^ = 42.1%, *P*_Q-test_ = 0.159); the heterogeneity levels significantly decreased in the subgroup analysis of “Liver injury model” of chemical liver injury (I^2^ = 42.1%, *P*_Q-test_ = 0.159) and liver ischemia-reperfusion injury (I^2^ = 1.2%, *P*_Q-test_ = 0.84). Moreover, subgroup analysis based on both “Administration route” and “Prophylactic or therapeutic” also resulted in a significant decrease in heterogeneity (Table 3).

**TABLE 3:** Results of subgroup analysis of the effects of ginsenoside Rb1 on inflammatory response indicators.

Indicators	Subgroup		No. of studies	SMD [95% CI]	*p*-value	I^2^	*p*-value for heterogeneity
TNF-α	Species	rats	4	−2.182 [−2.957 to −1.407]	0.000	42.1%	0.159
		mice	10	−4.707 [−6.722 to −2.692]	0.000	88.8%	0.000
	Model	liver injury in type 2 diabetic rats	4	−2.182 [−2.957 to −1.407]	0.000	42.1%	0.159
		chemical liver injury	6	−6.349 [−10.817 to −1.880]	0.005	0	0.929
		immunological liver injury	1	——	——	——	——
		drug-induced liver injury	1	——	——	——	——
		liver ischemia reperfusion injury	2	−4.239 [−8.891 to 0.413]	0.000	1.2%	0.84
	Administration	by intragastric	10	−3.683 [−5.020 to −2.347]	0.000	0	0.854
		by intraperitoneal injection	4	−3.379 [−7.203 to 0.445]	0.083	0	0.899
	Prophylactic or Therapeutic	therapeutic	5	−1.762 [−2.919 to −0.604]	0.003	72.2%	0.006
		prophylactic	9	−5.469 [−7.467 to −3.472]	0.000	87.9%	0.000
	Duration	≥30 days	4	−2.182 [−2.957 to −1.407]	0.000	42.1%	0.159
		≤7 days	7	−6.056 [−8.730 to −3.383]	0.000	0	0.901
		>7 days and <30 days	1	——	——	——	——
		once	2	−2.106 [−11.487 to 7.276]	0.66	0	0.938
IL-6	Species	rats	4	−2.684 [−3.771 to −1.598]	0.000	94.1%	0.036
		mice	9	−3.573 [−5.072 to −2.073]	0.000	85.5%	0.000
	Model	liver injury in type 2 diabetic rats	4	−2.684 [−3.771 to −1.598]	0.000	64.1%	0.039
		chemical liver injury	6	−2.686 [−4.281 to −1.090]	0.001	85.7%	0.000
		drug-induced liver injury	1	——	——	——	——
		liver ischemia reperfusion injury	2	−6.484 [−9.859 to −3.109]	0.000	52.4%	0.000
	Administration	by intragastric	9	−3.097 [−3.910 to −2.284]	0.000	64.3%	0.004
		by intraperitoneal injection	4	−3.639 [−7.120 to −0.158]	0.04	90.8%	0.000
	Prophylactic or Therapeutic	therapeutic	5	−2.589 [−3.532 to −1.645]	0.000	52.8%	0.076
		prophylactic	8	−3.781 [−5.418 to −2.144]	0.000	87.3%	0.000
	Duration	≥30 days	4	−2.684 [−3.771 to −1.598]	0.000	64.1%	0.039
		≤7 days	7	−3.356 [−4.961 to −1.752]	0.000	87.2%	0.000
		once	2	−5.169 [−11.896 to 1.558]	0.132	85.6%	0.008
IL-1β	Species	rats	6	−4.929 [−6.929 to −2.928]	0.000	1.6%	0.64
		mice	2	−1.866 [−2.638 to −1.095]	0.000	58.6%	0.000
	Model	liver injury in type 2 diabetic rats	2	−1.866 [−2.638 to −1.095]	0.000	58.6%	0.000
		chemical liver injury	3	−4.210 [−6.538 to −1.883]	0.000	63.6%	0.064
		drug-induced liver injury	1	——	——	——	——
		liver ischemia reperfusion injury	1	——	——	——	——
		alcoholic liver injury	1	——	——	——	——
	Administration	by intragastric	4	−3.095 [−4.686 to −1.504]	0.000	71.6%	0.014
		by intraperitoneal injection	4	−4.972 [−8.170 to −1.774]	0.002	77%	0.005
	Prophylactic or Therapeutic	therapeutic	4	−2.234 [−3.975 to −0.492]	0.012	71.3%	0.015
		prophylactic	4	−5.227 [−6.485 to −3.968]	0.000	0	0.975
	Duration	≥30 days	2	−1.866 [−2.638 to −1.095]	0.000	58.6%	0.000
		≤7 days	4	−6.015 [−8.555 to −3.475]	0.000	6.9%	0.577
		once	2	−3.254 [−6.029 to −0.480]	0.022	11.6%	0.598

Based on the data from the eight studies, Rg1 was observed to significantly reduce the levels of IL-1β in experimental animals (SMD = −3.84, 95% CI: −5.39 to −2.28, *p* = 0.000). However, the heterogeneity test revealed the existence of heterogeneity (I^2^ = 76.9%, *P*_Q-test_ = 0.000) (Figure 4B). The results of the subgroup analyses for IL-1β are presented in Table 3. Notably, none of the predetermined predefined subgroup analyses yielded a significant reduction in heterogeneity.

We collected data from 13 animal studies that evaluated IL-6 in liver injury (Figure 4C). Our meta-analysis revealed that Rg1 could significantly reduce IL-6 levels in liver injury (SMD = −3.19, 95% CI: −4.18 to −2.20, *p* = 0.006). Moreover, our heterogeneity test indicated high heterogeneity across the studies (I^2^ = 81.1%, *P*_Q-test_ = 0.000). In the results of the subgroup analysis, the heterogeneity of rats was significantly decreased for the subgroup analysis of “Animal species” (I^2^ = 1.6%, *P*_Q-test_ = 0.64), the heterogeneity of prophylactic was significantly decreased for the subgroup analysis of “Prophylactic or Therapeutic” (I^2^ = 0, *P*_Q-test_ = 0.975), and the heterogeneity of ≤7 days and once was significantly decreased for the subgroup analysis of “Duration of treatment” (I^2^ = 6.9%, *P*_Q-test_ = 0.577; I^2^ = 11.6%, *P*_Q-test_ = 0.598).

Several studies have identified additional inflammatory factors, including cyclooxygenase-2 (COX-2), inducible nitric oxide synthase (iNOS), interleukin-8 (IL-8), interleukin-10 (IL-10) and interferon-γ (IFN-γ), beyond the previously mentioned indicators. However, due to their limited availability in only one or two studies, a meta-analysis was not performed. The reported studies indicate that Rg1 plays a beneficial role in reducing the levels of these indicators, thereby positively contributing to the inflammatory response to liver injury.

#### 3.4.4 Sensitivity analysis

Each experiment on the effect of Rg1 on liver injury was excluded in turn. The results show that “Gao 2017” and “Gao 2016” may have a significant influence on the pooled effects of ALT, and the pooled effects excluding them are −2.306 (CI: −2.535 to −2.076) and −2.411 (CI: −2.650 to −2.171), respectively (Supplementary Figure S1).

#### 3.4.5 Publication bias

The risk of publication bias is shown in Supplementary Figure S2. An analysis of SMD values for ALT levels, a common indicator of liver injury, showed a publication bias (*p* = 0.001).

### 3.5 Efficacy of ginsenoside Rg3 in liver injury

#### 3.5.1 ALT and AST

**FIGURE 5:**
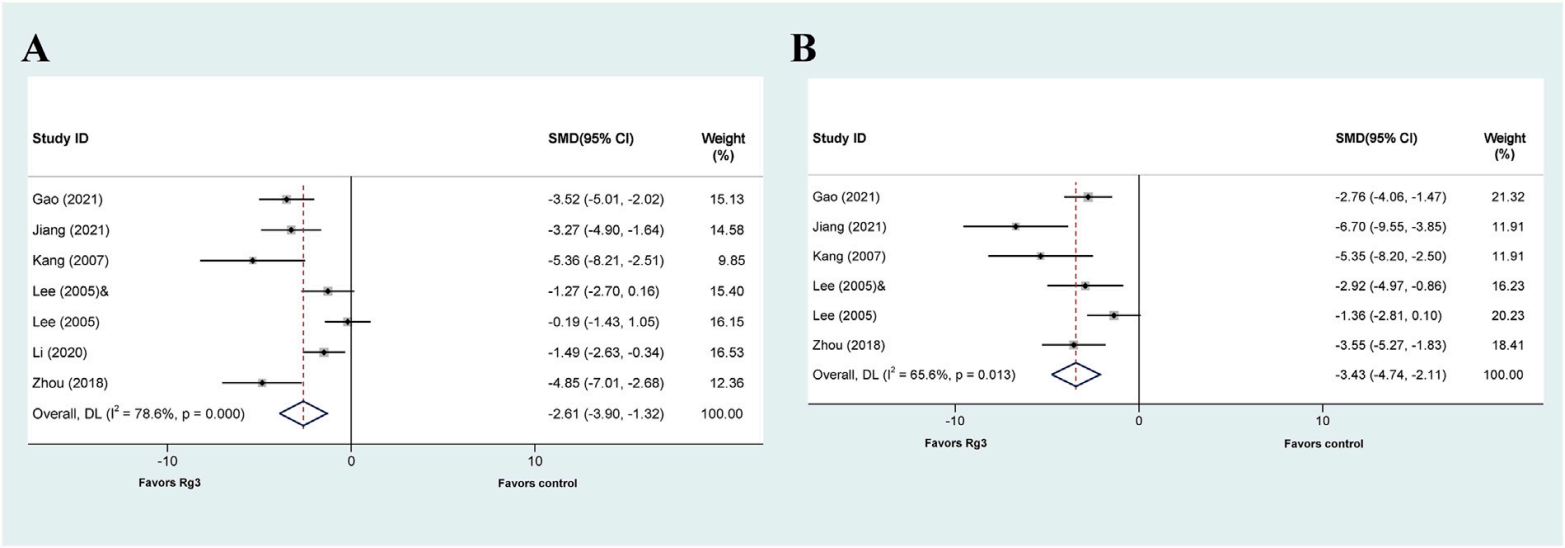
Standard mean differences estimates for the effects of ginsenoside Rg3 on **(A)** ALT and **(B)** AST.

Six out of the seven articles investigating the effect of Rg3 on liver injury reported the impact of ginsenosides on ALT levels. The meta-analysis results indicated that Rg1 could reduce ALT levels compared with the control group (SMD = −2.61, 95% CI: −3.90 to −1.32, *p* = 0.000; heterogeneity: I^2^ = 78.6%, *P*_Q-test_ = 0.000) (Figure 5A). Subsequently, we conducted a subgroup analysis that demonstrated no heterogeneity in the “model”-based subgroup concerning drug-induced liver injury (SMD = −3.946, 95% CI: −5.179 to −2.713, *p* = 0.000; heterogeneity: I^2^ = 0, *P*_Q-test_ = 0.322) (Table 4).

**TABLE 4:** Results of subgroup analysis of the effects of ginsenoside Rg3 on ALT and AST.

Indicators	Subgroup		No. of studies	SMD [95% CI]	*p*-value	I^2^	*p*-value for heterogeneity
ALT	Species	rats	1	——	——	——	——
		mice	6	−2.297 [−3.568 to −1.016]	0.000	78.0%	0.000
	Model	liver injury in type 2 diabetic rats	1	——	——	——	——
		chemical liver injury	4	−1.638 [−3.076 to -0.201]	0.0026	72.6%	0.012
		drug-induced liver injury	2	−3.946 [−5.179 to −2.713]	0.000	0	0.322
	Administration	by intragastric	6	−3.022 [−4.263 to −1.780]	0.000	70.1%	0.005
		by intraperitoneal injection	1	——	——	——	——
	Prophylactic or Therapeutic	therapeutic	2	−2.278 [−4.014 to −0.543]	0.010	67.4%	0.674
		prophylactic	5	−2.828 [−4.726 to −0.930]	0.003	84.0%	0.080
	Duration	≥30 days	2	−2.278 [−4.014 to −0.543]	0.010	67.4%	0.080
		≤7 days	4	−2.339 [−4.299 to −0.379]	0.019	84.6%	0.000
		>7 days and <30 days	1	——	——	——	——
AST	Species	rats	1	——	——	——	——
		mice	5	−3.149 [−4.502 to −1.796]	0.000	65.8%	0.020
	Model	liver injury in type 2 diabetic rats	1	——	——	——	——
		chemical liver injury	3	−2.927 [−5.046 to −0.809]	0.007	68.4%	0.042
		drug-induced liver injury	2	−3.047 [−4.083 to −2.012]	0.000	0	0.475
	Administration	by intragastric	5	−3.866 [−5.144 to −2.588]	0.000	50.1%	0.091
		by intraperitoneal injection	1	——	——	——	——
	Prophylactic or Therapeutic	therapeutic	1	——	——	——	——
		prophylactic	5	−2.890 [−3.974 to −1.807]	0.000	47.2%	0.109
	Duration	≥30 days	1	——	——	——	——
		≤7 days	4	−2.565 [−3.492 to −1.638]	0.000	26.7%	0.252
		once	1	——	——	——	——

The effect of Rg3 on AST levels in animal models of liver injury has been explored in five published articles. Similar to ALT analysis, the results of the meta-analysis demonstrate that Rg3 significantly reduce serum AST levels in animal models of liver injury. (SMD = −3.425, 95% CI: −4.742 to −2.109, *p* = 0.000; heterogeneity: I^2^ = 65.6%, *P*_Q-test_ = 0.013) (Figure 5B). The presence of heterogeneity is apparent in the findings. Subgroup analysis was then conducted, revealing no significant heterogeneity in the prophylactic subgroup in “Prophylactic or Therapeutic” (SMD = −2.890 95% CI: −3.974 to −1.807, *p* = 0.000; heterogeneity: I^2^ = 47.2%, *P*_Q-test_ = 0.109) as well as ≤7 days in “Duration” (SMD = −2.565, 95% CI: −3.492 to −1.638, *p* = 0.000; heterogeneity: I^2^ = 26.7%, *P*_Q-test_ = 0.252) (Table 4).

#### 3.5.2 Indicators of oxidative stress

Only one study has examined the effect of Rg3 on the levels of two necessary antioxidant enzymes, SOD and CAT. The results of this study indicate a significant increase in the levels of these enzymes in the Rg3-treated group compared to the model group.

Only two studies used GSH as an outcome indicator. Zhou et al. (2018) found that the Rg3-treated group had significantly higher GSH levels compared to the model group. Similarly, Gao et al. (2021) reported that the Rg3-treated group had significantly higher GSH levels than the model group. Additionally, Gao et al. (2021) identified differences in GSH-Px levels among the groups, and found that the Rg3-treated group had significantly lower GSH-Px levels compared to the model group.

The MDA data were obtained in three studies. The meta-analysis findings indicate that Rg3 decreased MDA levels in liver injuries (SMD = −3.639, 95% CI: −4.614 to −2.663, *p* = 0.000; heterogeneity: I^2^ = 0, *P*_Q-test_ = 0.408) (Supplementary Figure S3). Moreover, all predetermined subgroups in the three studies were consistent, except for “Model” and “Duration.” Therefore, we performed a subgroup analysis based on both “Model” and “Duration,” and the results were consistent (Supplementary Figure S3).

Only one study reported data on T-AOC. The results of Gao et al. (2021) showed that Rg3 significantly increased T-AOC.

#### 3.5.3 Indicators of inflammatory response

Only the TNF-α data were reported in this study by Jiang et al. (Jiang et al., 2021), whose results showed that Rg3 significantly reduced TNF-α levels in a liver injury model. The study by Gao et al. (Gao et al., 2021) noted that the Rg3-treated group significantly reduced the levels of IL-1β in a liver injury model. Finally, both of the above studies reported data on IL-6, and the results showed that IL-6 levels were significantly lower in the Rg3-treated group compared to the model group.

#### 3.5.4 Sensitivity analysis

After excluding each Rg3-related experiment in turn from the meta-analysis, there was no significant difference between the combined pre- and post-sensitivity pooled effects of ALT (Supplementary Figure S4). After omitting the data of “Lee 2005” and “Gao 2021,” respectively, the pooled effect values are the lowest (SMD = −2.763 CI: −3.401 to −2.124) and the highest (SMD = −1.984 CI: −2.598 to −1.369).

#### 3.5.5 Publication bias

The risk of publication bias is shown in the Egger publication bias plot (Supplementary Figure S5). The analysis of SMD values for ALT levels showed a statistically significant publication bias (*p* = 0.026).

### 3.6 Efficacy of ginsenoside Rb1 in liver injury

#### 3.6.1 ALT and AST

**FIGURE 6:**
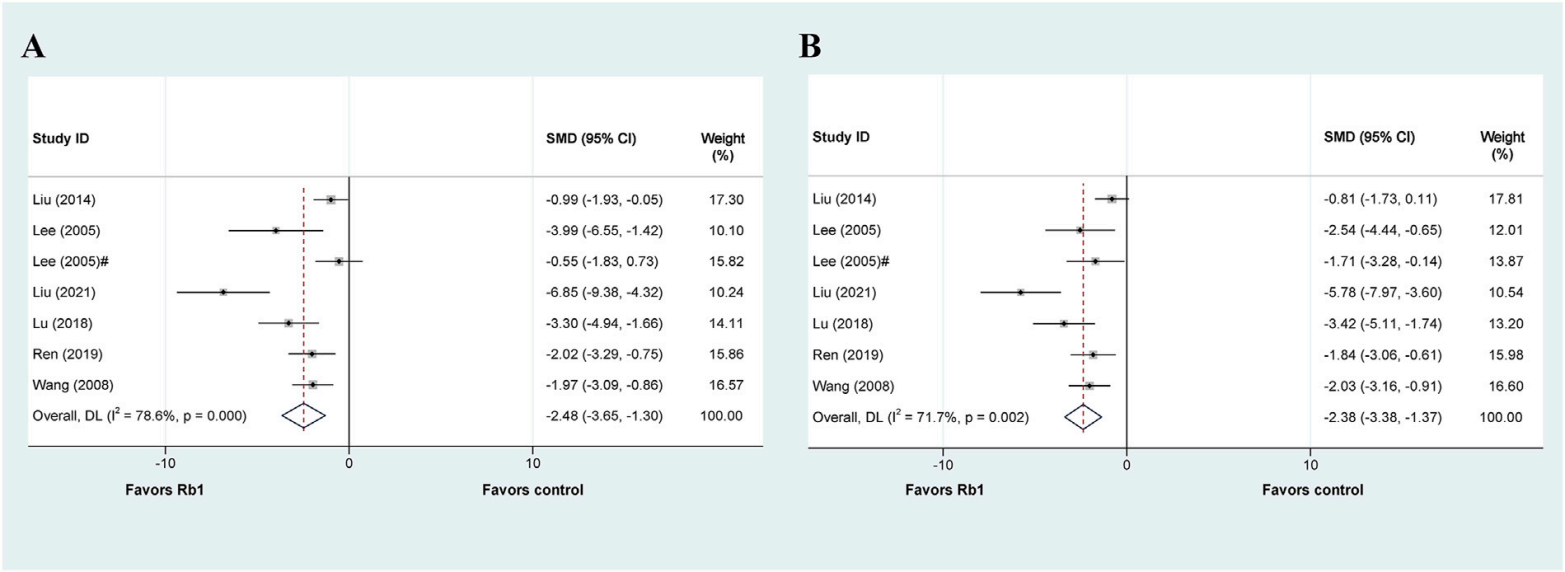
Standard mean differences estimates for the effects of ginsenoside Rb1 on **(A)** ALT and **(B)** AST.

Seven studies investigated the therapeutic or protective effects of ginsenoside Rb1 on liver injury, while six others focused on ALT levels, except for the study by Lai et al. (Lai et al., 2021). The meta-analysis results revealed that Rb1 effectively decreased ALT levels in liver injury (SMD = −2.48, 95% CI: −3.65 to −1.30, *p* = 0.000; heterogeneity: I^2^ = 78.6%, *P*_Q-test_ = 0.000) (Figure 6A). Out of the four subgroup analyses available, the subgroups based on “Model” for liver ischemia injury (SMD = −1.436, 95% CI: −2.397 to −0.475, *p* = 0.000; heterogeneity: I^2^ = 43.2%, *P*_Q-test_ = 0.185), by intraperitoneal injection based on “Administration” (SMD = −2.676, 95% CI: −4.489 to −0.862, *p* = 0.004; heterogeneity: I^2^ = 44.8%, *P*_Q-test_ = 0.178), and once based on “Duration” (SMD = −1.193, 95% CI: −1.977 to −0.409, *p* = 0.003; heterogeneity: I^2^ = 34.6%, *P*_Q-test_ = 0.217) showed no significant heterogeneity (Table 5).

**TABLE 5:** Results of subgroup analysis of the effects of ginsenoside Rb1 on ALT and AST.

Indicators	Subgroup		No. of studies	SMD [95% CI]	*p*-value	I^2^	*p*-value for heterogeneity
ALT	Species	rats	1	——	——	——	——
		mice	6	−2.823 [−4.208 to −1.438]	0.000	78.5%	0.000
	Model	liver ischemia reperfusion injury	2	−1.436 [−2.397 to −0.475]	0.003	43.2%	0.185
		chemical liver injury	4	−3.517 [−6.111 to −0.924]	0.008	86.7%	0.000
		drug-induced liver injury	1	——	——	——	——
	Administration	by intragastric	2	−2.676 [−4.489 to −0.862]	0.004	44.8%	0.178
		by intraperitoneal injection	4	−2.923 [−4.950 to −0.896]	0.005	85.9%	0.000
		by vein injection	1	——	——	——	——
	Duration	once	3	−1.193 [−1.977 to −0.409]	0.003	34.6%	0.217
		≤7 days	4	−3.818 [−5.693 to −1.943]	0.000	74.4%	0.008
AST	Species	rats	1	——	——	——	——
		mice	6	−2.687 [−3.689 to −1.684]	0.000	60.3%	0.027
	Model	liver ischemia reperfusion injury	2	−1.377 [−2.572 to −0.181]	0.024	63.3%	0.099
		chemical liver injury	4	−3.257 [−4.852 to −1.662]	0.007	67.7%	0.026
		drug-induced liver injury	1	——	——	——	——
	Administration	by intragastric	2	−2.045 [−3.073 to −1.017]	0.000	0.0%	0.538
		by intraperitoneal injection	4	−3.059 [−4.597 to −1.522]	0.000	73.3%	0.010
		by vein injection	1	——	——	——	——
	Duration	once	3	−1.423 [−2.235 to −0.611]	0.001	32.0%	0.230
		≤7 days	4	−3.246 [−4.810 to −1.683]	0.000	70.3%	0.018

The articles mentioned six additional studies that also presented data on AST. Our meta-analysis results show that Rb1 administration reduces AST levels in liver injury (SMD = −2.379, 95% CI: −3.384 to −1.373, *p* = 0.000; heterogeneity: I^2^ = 71.7%, *P*_Q-test_ = 0.002) (Figure 6B). We then performed a predefined subgroup analysis, which demonstrated no significant heterogeneity in the by intragastric based on “Administration” (SMD = −2.379, 95% CI: −3.384 to −1.373, *p* = 0.000; heterogeneity: I^2^ = 0.0%, *P*_Q-test_ = 0.538) and the once based on “Duration” (SMD = −1.423, 95% CI: −2.235 to −0.611, *p* = 0.001; heterogeneity: I^2^ = 32.0%, *P*_Q-test_ = 0.230) (Table 5).

#### 3.6.2 Indicators of oxidative stress

Two studies have reported the effect of Rb1 on SOD levels in liver injury. The data showed that Rb1 treatment could significantly increase SOD levels in animal models with liver injury (*p* < 0.05).

Three studies have investigated the effect of Rb1 on MDA levels in liver injury. Meta-analysis data indicate that Rb1 treatment significantly improves MDA levels in animal models with liver injury, and there was significant heterogeneity (SMD = −2.86, 95% CI: −5.12 to −0.60, *p* = 0.013; heterogeneity: I^2^ = 87.9%, *P*_Q-test_ = 0.000) (Supplementary Figure S6). The results of the pre-defined subgroup grouping are presented in Supplementary Table S2. There was no significant heterogeneity in the liver ischemia-reperfusion injury based on the “Model” (SMD = −2.993, 95% CI: −7.215 to 1.229, *p* = 0.000; heterogeneity: I^2^ = 42.1%, *P*_Q-test_ = 0.159) and the once based on “Duration” (SMD = −2.993, 95% CI: −7.215 to 1.229, *p* = 0.000; heterogeneity: I^2^ = 42.1%, *P*_Q-test_ = 0.159).

Ren et al. (Ren et al., 2019) and Liu et al. (Liu et al., 2021) presented data on GSH and GSH-Px, respectively. The findings showed that treatment with Rb1 significantly increased the levels of GSH or GSH-Px in animal models with liver injury (*p* < 0.01 or *p* < 0.05).

Two studies were reported on MPO, and their results showed that Rb1 treatment significantly reduced MPO levels in animal models of liver injury (*p* < 0.05).

Only one study reported the effect of Rb1 treatment on ROS in an animal model of liver injury, and their results showed that Rb1 treatment significantly reduced ROS levels compared to the model group (*p* < 0.05).

#### 3.6.3 Indicators of inflammatory response

Three studies investigated the effect of Rb1 treatment on TNF-α in animal models of liver injury. The results of the meta-analysis indicate that Rb1 can effectively reduce TNF-α levels in animal models, with significant heterogeneity (SMD = −4.377, 95% CI: −7.207 to −1.546, *p* = 0.002; heterogeneity: I^2^ = 86.1%, *P*_Q-test_ = 0.001) (Supplementary Figure S7). Although predefined subgroup analysis was conducted, it failed to find any significant reduction in heterogeneity (Supplementary Table S3).

Two studies have reported IL-1β data, and their results suggest that Rb1 treatment can significantly reduce its levels in liver injury models (*p* < 0.05). Two studies have reported the effect of Rb1 on IL-6. The reported data showed that IL-6 levels were significantly lower in the Rb1 treatment group than in the model group (*p* < 0.05).

In addition, studies have reported the effect of Rb1 on the levels of two inflammatory cytokines (IL-8 and IL-18) (Lu et al., 2018; Liu et al., 2021). Their reported results suggest that Rb1 can significantly reduce their levels of liver injury (*p* < 0.05).

#### 3.6.4 Sensitivity analysis

The seven trials included in the meta-analysis were excluded in turn, and the results showed that the previously observed results on the effect of Rb1 on ALT were not altered (Supplementary Figure S8). Notably, after the exclusion of “Liu 2014” and “Liu 2021,” the pooled effect values are the lowest (SMD = −2.384 CI: −2.986 to −1.783) and highest (SMD = −1.770 CI: −2.287 to −1.252) respectively.

#### 3.6.5 Publication bias

The Egger’s regression test was performed on the included Rb1-related trials. The Egger publication bias plot showed a significant publication bias (*p* = 0.013) (Supplementary Figure S9).

### 3.7 Efficacy of ginsenoside CK in liver injury

#### 3.7.1 ALT and AST

**FIGURE 7:**
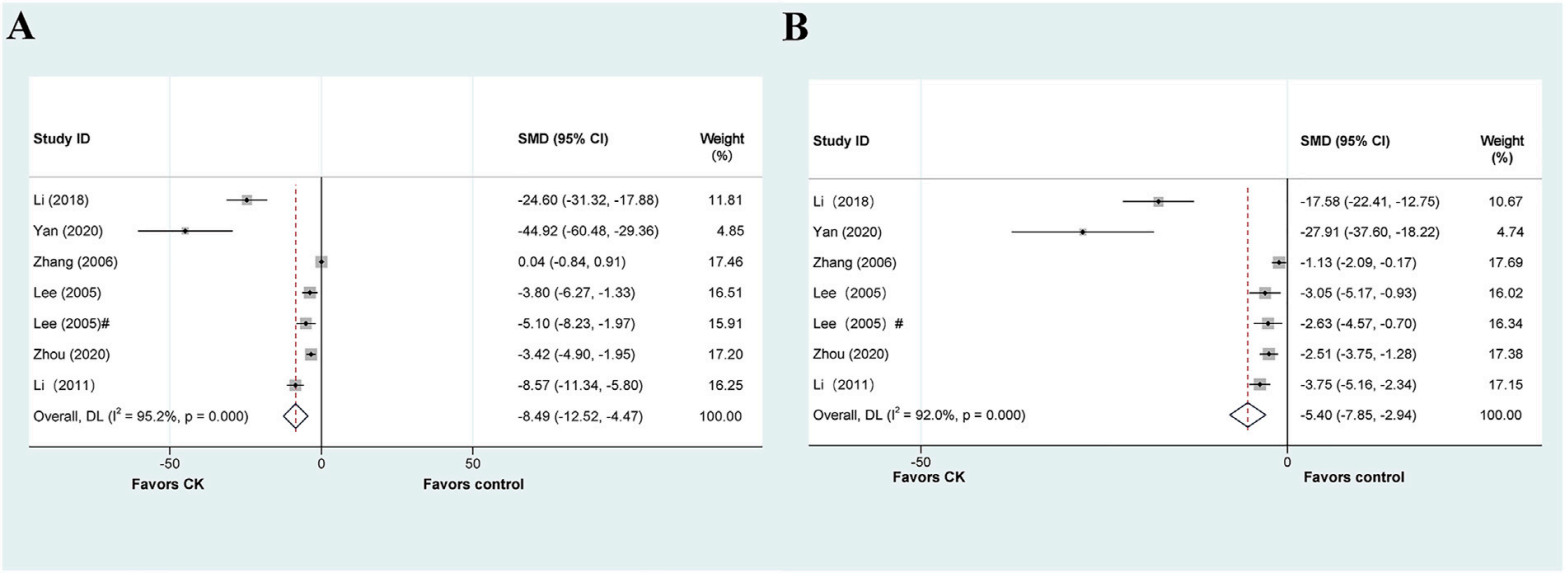
Standard mean differences estimates for the effects of ginsenoside CK on **(A)** ALT and **(B)** AST.

Seven articles explored the therapeutic or prophylactic effects of CK on liver injury. Among these, five studies reported the effect of CK treatment on ALT levels in animal models of liver injury. It is worth noting that Lee et al. (Lee H. U. et al., 2005) conducted the experiment with two administrations (by intragastric and intraperitoneal injection) in the article. The meta-analysis results of ALT data are shown in Figure 7A, and the results show that CK treatment can significantly reduce ALT in animal models of liver injury (SMD = −8.495, 95% CI: −12.518 to −4.472, *p* = 0.000; heterogeneity: I^2^ = 98.5%, *P*_Q-test_ = 0.000). A predefined subgroup analysis was performed, but no subgroup showed significantly reduced heterogeneity (Table 6).

**TABLE 6:** Results of subgroup analysis of the effects of ginsenoside CK on ALT and AST.

Indicators	Subgroup		No. of studies	SMD [95% CI]	*p*-value	I^2^	*p*-value for heterogeneity
ALT	Species	rats	4	−12.02 [−18.417 to −5.623]	0.000	96.8%	0.000
		mice	3	−5.796 [−8.698 to −2.894]	0.000	69.4%	0.038
	Model	Liver injury in type 2 diabetic rats	1	——	——	——	——
		chemical liver injury	4	−4.233 [−8.378 to −0.088]	0.045	93.3%	0.000
		drug-induced liver injury	1	——	——	——	——
		alcoholic liver injury	1	——	——	——	——
	Administration	by intragastric	6	−9.330 [13.899 to −4.761]	0.000	95.9%	0.000
		by intraperitoneal injection	1	——	——	——	——
	Prophylactic or Therapeutic	prophylactic	4	−5.06 [−7.313 to −2.807]	0.000	72.7%	0.013
		therapeutic	3	−22.115 [−45.504 to 1.274]	0.064	97.6%	0.000
	Duration	once	1	——	——	——	——
		>7 days and <30 days	4	−7.801 [−12.931 to −2.670]	0.003	96.6%	0.000
		≥30 days	1	——	——	——	——
		≤7 days	1	——	——	——	——
AST	Species	rats	4	−9.269 [−14.148 to −4.390]	0.000	95.8%	0.000
		mice	3	−3.293 [−4.296 to −2.290]	0.000	0.0%	0.636
	Model	Liver injury in type 2 diabetic rats	1	——	——	——	——
		chemical liver injury	4	−2.545 [−3.957 to −1.133]	0.000	70.7%	0.017
		drug-induced liver injury	1	——	——	——	——
		alcoholic liver injury	1	——	——	——	——
	Administration	by intragastric	6	−6.186 [−9.085 to −3.287]	0.000	93.3%	0.000
		by intraperitoneal injection	1	——	——	——	——
	Prophylactic or Therapeutic	prophylactic	4	−2.982 [−3.760 to −2.203]	0.000	0.0%	0.608
		therapeutic	3	−14.985 [−30.311 to 0.341]	0.055	97.2%	0.000
	Duration	once	1	——	——	——	——
		>7 days and <30 days	4	−4.954 [−7.945 to −1.963]	0.001	93.8%	0.000
		≥30 days	1	——	——	——	——
		≤7 days	1	——	——	——	——

The above six articles have reported AST data. Our meta-analysis demonstrates that CK has a significantly impact on reducing AST levels in liver injury models. However, we observed considerable heterogeneity (SMD = −5.398, 95% CI: −7.853 to −2.942, *p* = 0.000; heterogeneity: I^2^ = 97.2%, *P*_Q-test_ = 0.000) (Figure 7B). By predefined subgroup analysis, we found no significant heterogeneity in the mice based on “Species” (SMD = −3.293, 95% CI: −4.296 to −2.290, *p* = 0.000; heterogeneity: I^2^ = 0.0%, *P*_Q-test_ = 0.636) and the prophylactic based on “Prophylactic or Therapeutic” (SMD = −2.644, 95% CI: −3.578 to −1.711, *p* = 0.000; heterogeneity: I^2^ = 0.0%, *P*_Q-test_ = 0.912).

#### 3.7.2 Indicators of oxidative stress

Three studies investigating the effect of CK on SOD in liver injury models. The results of the meta-analysis showed that CK could significantly enhanced SOD levels in animal models with liver injury; however, significant heterogeneity was observed (SMD = 5.107, 95% CI: 1.865 to 8.349, *p* = 0.002; heterogeneity: I^2^ = 98.4%, *P*_Q-test_ = 0.000) (Supplementary Figure S10). Supplementary Table S4 presents the predefined subgroup analyses that were performed, with the “Prophylactic or Therapeutic” subgroup showing no significant results (*p* = 0.289 and *p* = 0.143). Two studies provided data on the effect of CK on CAT levels, both of which demonstrated a significant increase in CAT levels following CK treatment compared to the model group (*p* < 0.05).

Two studies reported data on GSH levels, and both showed that CK treatment significantly increased GSH levels in animal models of liver injury (*p* < 0.05). Only one study reported the effect of CK treatment on GSH-Px levels, and it found that CK treatment significantly increased GSH-Px levels in the treated group compared to the model group (*p* < 0.05).

Three studies investigated the effect of CK treatment on the MDA levels in liver injury. The meta-analysis results, shown in Supplementary Figure S11, indicated that CK treatment significantly reduced MDA levels in these animal models (SMD = −3.102, 95% CI: −6.059 to −0.145, *p* = 0.040; heterogeneity: I^2^ = 92.0%, *P*_Q-test_ = 0.000). A subgroup analysis was also performed, there was a significant reduction in the heterogeneity of prophylactic entries in the “Prophylactic or Therapeutic” subgroup (SMD = −4.233, 95% CI: −5.834 to −2.633, *p* = 0.000; heterogeneity: I^2^ = 47.9%, *P*_Q-test_ = 0.166) (Supplementary Table S5).

#### 3.7.3 Indicators of inflammatory response

Out of all the studies related to CK that we included, only Yan et al. (Yan et al., 2020) reported on the effect of CK on inflammatory factors. This study found that CK treatment significantly reduced inflammatory factors, including TNF-α, IL-6, and IL-1β, in the liver injury model of type 2 diabetes rats.

#### 3.7.4 Sensitivity analysis

The pooled effect values after the sequential exclusion of the included trials are shown in Supplementary Figure S12. The results show that the pooled effect value was the lowest after “Zhang 2006 was excluded (SMD = −10.663 CI: −15.469 to −5.857).

#### 3.7.5 Publication bias

The risk analysis of publication bias for included trials is shown in Supplementary Figure S13. There may be significant publication bias in the included studies of CK-related liver injury (*p* = 0.001).

### 3.8 Efficacy of other ginsenoside in liver injury

In addition to the previously mentioned four ginsenoside, we also reviewed studies investigating the effects of other ginsenosides on liver injury. Due to the limited number of included studies (two or less), a meta-analysis was not be performed. Nevertheless, these studies demonstrate that several ginsenosides have positive effects on liver injury. For instance, the study by Wang et al. (Wang et al., 2017) revealed that Rg5 offers liver protection against acute hepatotoxicity induced by APAP; and the experiment of Kim et al. (Kim et al., 2020) demonstrated that F2 treatment alleviates alcoholic liver injury.

## 4 Discussion

This review and meta-analysis systematically assesses the effects of ginsenosides on liver injury caused by multiple factors in different animal models. The accumulated data from the included studies indicate that multiple ginsenosides significantly reduce ALT and AST levels in liver injury models. However, there is suspected publication bias, as shown by Egger’s linear regression test on the accumulated results of various ginsenosides (*p* < 0.05). Therefore, the role of multiple ginsenosides in the prevention or treatment of liver injury may be overestimated. This may occur because 1) studies conducted on animals are characterized by small sample sizes per group and, therefore, the results of the analysis may be overestimated; and 2) studies reporting negative or no treatment effects are not often published (Radice et al., 2021). Four ginsenosides, Rg1 (*N* = 22), Rg3 (*N* = 7), Rb1 (*N* = 7), and CK (*N* = 7), have been extensively studied in relation to liver injury, and a meta-analysis was conducted on each one. No statistical differences between dose groups were reported in any of the included studies. We employed a strategy of analyzing multiple dose combined into a single dose group; therefore, we could not performed meta-regression analyses or subgroup analyses based on dose (Wu et al., 2022). Significant heterogeneity was observed in the meta-analysis of ALT and AST. For the effects of Rg1 on ALT and AST in liver injury, we performed a meta-regression analysis. However, the results showed that the species of experimental animal, types of liver injury models, administration route, prophylactic or therapeutic, and duration of treatment were not sources of heterogeneity among studies. Consequently, we did not conduct subsequent subgroup analysis. For Rg3, the types of liver injury models were identified as a potential source of ALT heterogeneity, and prophylactic or therapeutic and duration of treatment were identified as potential source of AST heterogeneity. In the case of Rb1, the types of liver injury models, administration route, and the duration of treatment were all possible sources of ALT heterogeneity, whereas administration route and duration of treatment were identified as potential source of AST heterogeneity. Finally, for CK, the species of experimental animal and prophylactic or therapeutic could be sources of AST heterogeneity. The reduction in heterogeneity observed in the subgroup analysis suggests that ginsenosides have different effects on different liver injury models, dosing times and dosing methods. Thus, the efficacy of ginsenosides may vary depending on the administration routes, duration of treatment, and models of liver injury.

There is evidence that oxidative stress and inflammation play critical roles in all types of liver injury (Liu et al., 2022). The liver is a vital metabolic organ that demands high energy and is thus composed of numerous mitochondria. When liver damage occurs, damaged mitochondria produce excess of ROS. Such hepatotoxic compounds or other factors can interfere with the electron transport chain located in the mitochondrial membrane, leading to excessive ROS production. Most of the included studies conducted oxidative stress-related studies investigations, which revealed that various ginsenosides significantly reduced the level of MDA and increase the level of SOD, GSH, GSH-Px, and CAT in liver injury. However, there was high heterogeneity among studies in the results of the meta-analysis. Interestingly, in the subgroup analysis for Rg1, the high heterogeneity of SOD may be associated with different liver injury models, while the heterogeneity of GSH may be related to prophylactic or therapeutic use. Additionally, the heterogeneity of GSH-Px data may be related to the duration of treatment and the liver injury model. These subgroup analysis results suggest that the effect of Rg1 on oxidative stress in liver injury may be related to the type of liver injury, prophylactic or therapeutic, and treatment duration. In the case of Rb1 data, the source of MDA heterogeneity may be related to the liver injury model and duration of treatment. Notably, the effect of CK on SOD and MDA levels in liver injury was not significant based on predefined subgroup analysis. These results imply that different ginsenosides may have varying effects on oxidative stress. However, it is premature to determine which ginsenoside is superior to others in dealing with oxidative stress in liver injury due to the limited relevant research data currently available. Moreover, multiple sources of high heterogeneity have not been identified.

Liver injury typically triggers an inflammatory response. In turn, persistent inflammation with elevated inflammatory factors can lead to extensive liver injury and the progression of chronic liver disease and even liver fibrosis (Seitz et al., 2018). Consequently, we focused on the effect of ginsenosides on inflammatory responses in models of liver injury. Existing data indicate that various ginsenosides can effectively reduce inflammatory factors in liver injury models, including TNF-α, IL-1β, and IL-6. However, due to the limited number of included studies, we performed a meta-analysis solely on the data collected for TNF-α, IL-1β, and IL-6 in Rg1, and TNF-α in Rg3. Nevertheless, high heterogeneity still existed in these results. The sources of heterogeneity in the Rg1 study included several of our predefined subgroup analysis items and may account for the heterogeneous TNF-α sources (Table 3). For IL-6 data, heterogeneity source may be associated with test animal species, prophylactic or therapeutic treatment, and treatment duration. No sources of heterogeneity were discovered for other data.

Despite conducting meta-regression analyses, subgroup analyses, and sensitivity analyses, we still observed unexplained heterogeneity. We hypothesize that these heterogeneities might originate, at least in part, from differences in the methodological design of liver injury models. Even in cases where the same model of liver injury was used, differences in modeling methods existed. For example, in the APAP-induced liver injury model, the dose of APAP varied among studies. However, due to the differences in the design of these methods and the limited number of studies, it was not possible to explore them by meta-regression or subgroup analyses. Therefore, we used a random-effect model to pool effect sizes. In fact, the vast majority of meta-analyses for animal model studies also utilize a random-effect model.

To assess the quality of animal experiments conducted in liver injury studies, we utilized SYRCLE’s risk of bias tool, which is specifically designed for animal studies (Hooijmans et al., 2014). The assessment of the risk of bias revealed that all studies included in the analysis obtained a score ranging from 2 to 3. The results indicate a need for improvement in animal studies involving liver injury needs to be improved. Given the low methodological quality of the studies analyzed, it is necessary to exercise caution when interpreting the data.

The protective and therapeutic effects of ginsenosides on liver injury are currently under investigation with a focus on apoptosis, inflammation, and oxidative stress. Various ginsenosides, including Rb1, Rg1, Rg3, Rg5, Rh1, Rh2, and CK, have anti-inflammatory effects and are promising in treating inflammation-related diseases (Kim et al., 2017). Additionally, the mechanism of ginseng on oxidative stress has also been extensively studied (He et al., 2022). Despite its low content in ginseng, Rg1 was the most studied ginsenoside, and it has been found to have anti-liver injury effects in various models. In the type Ⅱ diabetic and ischemia/reperfusion liver injury models, Rg1 significantly improving inflammatory response factors, oxidation indices, and lipid levels, while exhibiting anti-apoptotic effects and inhibit the activation of c-Jun N-terminal kinase (JNK) signaling pathway (Zhang et al., 2015; Tian et al., 2017). Rg1 may also restrict the mitochondrial apoptosis pathway and the Hippo-Yap pathway (Lin et al., 2020; Zhang et al., 2021). In models of drug-induced liver injury and chemical liver injury, Rg1 protects against liver injury by activating the nuclear factor E2-related factor 2 (Nrf2) signaling pathway, inhibiting the nuclear factor-kappa B (NF-κB)/NLRP3 inflammasome signaling pathway, and toll-like receptor 4 (TLR4) signaling pathway, and activating the AMP-activated protein kinase (AMPK) signaling pathway (Xin et al., 2016;Gao et al., 2017;Ning et al., 2018a;b;Ning et al., 2018c;Zhao et al., 2021) Collectively, multiple preclinical studies have demonstrated its efficacy by establishing different causes of liver injury and further exploring the mechanism of action, so that Rg1 can be a promising candidate for anti-liver injury agents.

In addition to Rg1, the protective effects of Rg3, Rb1, and CK on liver injury have also received attention. Similar to Rg1, Rg3 exhibits anti-inflammatory activity by targeting NLRP3 in APAP-induced liver injury (Gao et al., 2021). Moreover, Rg3 has been shown to alleviate apoptosis and inflammation in liver injury by upregulating PI3K/Akt signaling pathways (Zhou et al., 2018; Li et al., 2020). Rg3 can also downregulate the levels of angiotensin II (Ang II) and activate the peroxisome proliferator-activated receptor gamma (PPARγ) pathway in type II diabetic mice, (Jiang et al., 2021). Notably, Rg3 is the only ginsenoside reported to alleviating septic liver injury (Wu et al., 2021). Studies related to Rg1 have shown that its effect on liver injury involves the regulation of JNK/ERK/p38 MAPKs, PI3K/Akt signaling pathways, and the NF-κB signaling pathway (Ren et al., 2019; Lai et al., 2021; Liu et al., 2021). The TGF-β/Smad pathway may also be involved in the mechanism by which Rg3 protects against CCl_4_-induced liver injury (Lu et al., 2018). Ginsenoside CK [20-O-beta-D-glucopyranosyl-20(S)-protopanaxadiol; also known as M1, compound K, IH901] is a main active metabolite of 20(S)-protopanaxadiol type ginsenoside (Kim, 2013). In the sodium valproate-induced hepatotoxicity model, CK upregulates the antioxidant defense system to inhibit oxidative stress, which may be related to its regulatory effect on the peroxisome pathway (Zhou et al., 2020).

The present study has several limitations. Firstly, although the number of studies included in this work is comparable to that of other meta-analyses, we performed separate meta-analyses for different ginsenosides. Thus, the available data on each ginsenoside is still limited. Additionally, due to the unavailability of data, we could only describe some ginsenosides and indicators without meta-analysis. Secondly, the majority of the included animal studies had small sample sizes (6–10 animals per group), which may have compromised the overall quality of the evidence. Thirdly, the SYRCLE’s assessment indicated that the quality of the included studies was generally low, which in turn weakened the strength of the evidence in this meta-analysis. Fourthly, despite conducting meta-regression analysis and subgroup analysis based on predefined moderating variables, a considerable number of results showed significant heterogeneity. This heterogeneity suggests that the effect of ginsenosides on liver injury may have been overestimated due to suspected publication bias.

## 5 Conclusion

According to our study, various ginsenosides demonstrate a significantly potential in improve the animal model of liver injury. The mechanism of ginsenosides on different liver injury models may be different, and the possible mechanism is mainly embodied in antioxidant, anti-inflammatory, and apoptosis inhibition. The most researched ginsenoside is Rg1, yet it is uncertain which ginsenoside has the best efficacy. Notably, the included studies were of low quality and publication bias might have overestimated the effect of ginsenosides on liver injury. Hence, although ginsenosides exhibit promising potential for liver injury prevention and treatment, the limitations of existing preclinical studies make their clinical application challenging. To more accurately assess the efficacy of ginsenosides, future studies require a larger sample size and a more rigorous methodological design.

## Abbreviations

ALT, alanine aminotransferase; AST, aspartate aminotransferase; APAP, acetaminophen; AMPK, AMP-activated protein kinase; Ang II, angiotensin II; CNKI, China National Knowledge Infrastructure; CK, compound K; CAT, catalase; COX-2, cyclooxygenase-2; Con A, Concanavalin A; D-Gal, D-galactosamine; JNK, c-Jun N-terminal kinase; GSH, glutathione; GSH-Px, glutathione peroxidase; IL-1β, interleukin-1β; IL-6, interleukin-6; iNOS, inducible nitric oxide synthase; IL-8, interleukin-8; IL-10, interleukin-10; IL-18, interleukin-18; IFN-γ, interferon-γ; LPS, lipopolysaccharide; MDA, malondialdehyde; MeSH, medical subject headings; MPO, myeloperoxidase; Nrf2, nuclear factor E2-related factor 2; NF-κB, nuclear factor-kappa B; PPD, protopanaxadiol; PPT, protopanaxatriol; PPARγ, peroxisome proliferator-activated receptor γ; ROS, reactive oxygen species; SOD, superoxide dismutase; SE, standard errors; SD, standard deviations; SMD, standard mean difference; TNF-α, tumour necrosis factor-α; T-AOC, total antioxidant capacity; TLR4, toll-like receptor 4; t-BHP, tert-butyl hydroperoxide.
