# Supplementary material for: The effect of ginsenosides on liver injury in preclinical studies: a systematic review and meta-analysis
Source: Front Pharmacol. 2023 May 11;14:1184774. doi: 10.3389/fphar.2023.1184774 (PMC10213882; doi:10.3389/fphar.2023.1184774)
Supplement: Supplementary file 1 [file Table1.docx]

|  |  |  |  |  | **Intervention** |  |  |  |  |
| --- | --- | --- | --- | --- | --- | --- | --- | --- | --- |
| **Study** | **Ginsenoside** | **Species (strain; sex; age; weight)** | **Sample size (T/V)** | **Modeling method (establish)** | **Dose** | **Source** | **Administration/duration** | **Outcomes** | **Intergroup difference** |
| Qi (2016) | Rg1 | mice (Kunming; male; NA; 150-180g) | 16, 16, 16/16 | CCl_4_ | 10, 20, 40 mg·kg^-1^ | Shanghai Yihe Biotechnology Co., LTD | By intragastric/pretreatment 7 days | **1. Blood indicators**: AST↓, ALT↔ MDA↔, SOD (10 mg·kg-1); AST↓, ALT↓, MDA↓, SOD↑ (20 mg·kg^-1^); AST↓, ALT↓, MDA↓, SOD↑ (40 mg·kg^-1^). **2. HE Staining.** | 1. p < 0.05 |
| Zhang (2006) | CK | Rats（Wistar; NA; NA; NA) | 10, 10, 10/10 | CCl_4_ | 0.3, 1, 3 mg·kg^-1^ | Shandong Natural Medicine Engineering Technology Research Center | By intragastric/4 weeks | **1. Blood indicators**: ALT↑, AST↑, MDA↓, SOD↑, HA↓, PCⅢ↓ (0.3 mg·kg^-1^); ALT↔, AST↔, MDA↔, SOD↑, HA↓, PCⅢ↔ (1 mg·kg^-1^); ALT↔, AST↔, MDA↔, SOD↔, HA↔, PCⅢ↔ (3 mg·kg^-1^). **2. HE Staining.** | 1. p < 0.01 or p < 0.05. |
| Li (2011) | CK | mice (ICR; male; NA; 22-25g) | 12/12 | CCl_4_ | 30 mg·kg^-1^ | Laboratory preparation | By intragastric/ 4 weeks | **1. Liver weight**↓ (30 mg·kg^-1^). **2. Blood indicators**: ALT↓, AST↓ (30 mg·kg^-1^). **3. Liver tissue indicators**: SOD↑, MDA↓ (30 mg·kg^-1^). **4. HE Staining.** | 1. p < 0.05, 2. p < 0.05, 3. p < 0.05. |
| Lu (2018) | Rg1 | mice (ICR; male; NA; NA) | 8/8 | CCl_4_ | 30 mg·kg_-1_ | Sigma-Aldrich Chemical Co. LTD | By intraperitoneal injection/pretreatment 7 days | **1. HE staining**. **2. Blood indicators**: ALT↓, AST↓ (30 mg·kg^-1^). **3. Liver tissue indicators**: IL-6↓, IL-8↓ (30 mg·kg^-1^). **4. Gene expression**: TGF-β↓, Smad2↓, Smad3↓ (30 mg·kg^-1^). **5. Protein expression**. | 1. p < 0.05, 2. p < 0.05, 3. p < 0.05, 4. p < 0.05. |
|  | Rb1 | mice (ICR; male; NA; NA) | 8/8 | CCl_4_ | 30 mg·kg_-1_ | Sigma-Aldrich Chemical Co. LTD | By intraperitoneal injection/pretreatment 7 days | **1. HE staining**. **2. Blood indicators**: ALT↓, AST↓ (30 mg·kg^-1^). **3. Liver tissue indicators**: IL-6↓, IL-8↓ (30 mg·kg^-1^). **4. Gene expression**: TGF-β↓, Smad2↓, Smad3↓ (30 mg·kg^-1^). **5. Protein expression**. | 1. p < 0.05, 2. p < 0.05, 3. p < 0.05, 4. p < 0.05. |
| Ning (2018) | Rg1 | mice (C57BL/6; male; 8 weeks; NA) | 6, 6, 6/6 | CCl_4_ | 15, 30, 60 mg·kg^-1^ | Baoji Herbest Biotechnology Co., LTD | By intragastric/pretreatment 7 days | **1. Liver weight**↓ (15 mg·kg^-1^); Liver weight↓ (30 mg·kg^-1^); Liver weight↓ (60 mg·kg^-1^). **2. Blood indicators**: total protein↔, ALT↔, AST↔, ALP↔ (15 mg·kg^-1^); total protein↓, ALT↓, AST↓, ALP↓ (30 mg·kg^-1^); total protein↓, ALT↓, AST↓, ALP↓ (60 mg·kg^-1^). **3. HE Staining**: necrosis score↔ (15 mg·kg^-1^); necrosis score↓ (30 mg·kg^-1^); necrosis score↓ (60 mg·kg^-1^). **4. Liver tissue indicators**: SOD↑, MDA↓, GSH↑, CAT↑ (15 mg·kg^-1^); SOD↑, MDA↓, GSH↑, CAT↑ (30 mg·kg^-1^); SOD↑, MDA↓, GSH↑, CAT↑ (60 mg·kg^-1^). **5. Protein expression**: TNF-α↓, IL-1β↓, IL-6↓, COX-2↓, iNOS↓, Keap1↓, Nrf2↑, NQO1↑, GCLM↑, Bsep↑, Mrp2↑, Mrp3↑, Mrp4↑ (60 mg·kg^-1^). **6. Gene expression**: GCLC↑, HO-1↑, CYP2E1↓ (60 mg·kg^-1^). **7. Immunohistochemistry**: BrdU-positive hepatocytes↑ (15 mg·kg^-1^); BrdU-positive hepatocytes↑ (30 mg·kg^-1^); BrdU-positive hepatocytes↑ (60 mg·kg^-1^). | 1. p < 0.05, 2. p < 0.05, 3. p < 0.05, 4. p < 0.05, 5. p < 0.05, 6. p < 0.05, 7. p < 0.05. |
| Qi (2017) | Rg1 | mice (Kunming; male; 8-10 weeks; 20±2g) | 8, 8, 8/8 | CCl_4_ | 10, 20, 40 mg·kg^-1^ | Shanghai YaJi Biotechnology Co., LTD. | By intragastric/pretreatment 7 days | **1. HE Staining**. **2. Blood indicators**: ALT↔, AST↓, TNF‑α↔, IL‑6↔ (10 mg·kg^-1^); ALT↓, AST↓, TNF‑α↓, IL‑6↓ (20 mg·kg^-1^); ALT↓, AST↓, TNF‑α↓, IL‑6↓ (40 mg·kg^-1^). **3. Liver tissue indicators**: SOD↑, MDA↔ (10 mg·kg^-1^); SOD↑, MDA↓ (20 mg·kg^-1^); SOD↑, MDA↓ (40 mg·kg^-1^). **4. Protein expression**: p65↓ (10 mg·kg^-1^); p65↓ (20 mg·kg^-1^); p65↓ (40 mg·kg^-1^). | 2. p < 0.05, 3. p < 0.05, 4. p < 0.05. |
| Zhao (2021) | Rg1 | mice (C57BL/6; male; 6-8 weeks; NA) | 3/3 | CCl_4_ | 0.2 ml (4 mg/ml) | Yunnan, Jicui Biotechnology Co., LTD. | By intraperitoneal injection/once | **1. Blood indicators**: ALT↓, AST↓, TNF-α↓, IL-1β↓, IL-6↓ (4 mg/ml). **2. HE Staining**. **3. Flow cytometry**: autophagy↑, apoptotic↓ (4 mg/ml). **4. TUNEL staining**: Apoptotic↓ (4 mg/ml). **5. Gene expression**: NF-κB↓, NLRP3↓, cleaved caspase 1↓, cleaved caspase 3↓, IL-18↓, IL-1β↓, LC3-II↔, Beclin-1↓, PINK1↑, Parkin↑ (4 mg/ml). **6. Protein expression**. | 1. p < 0.05 or p < 0.01, 2. p < 0.05, 3. p < 0.05,  4. p < 0.05, 5. p < 0.05 or p < 0.01. |
| Yao (2016) | Rg1 | mice (Kunming; male; NA; 18-22g) | 10, 10/10 | CCl_4_ | 20, 40 mg·kg^-1^ | National Institutes for Food and Drug Control of China | By intragastric/pretreatment 7 days | **1. Blood indicators**: ALT↓, AST↓, TNF-α↓, IL-6↓ (20 mg·kg-1); ALT↓, AST↓, TNF-α↓, IL-6↓ (40 mg·kg^-1^). **2. Liver tissue indicators**: ALT↓, AST↓, MDA↓, MPO↓, SOD↑ (20 mg·kg^-1^); ALT↓, AST↓, MDA↓, MPO↓, SOD↑ (40 mg·kg^-1^). **3. HE staining**. **4. Gene expression**: TNF-α↓, IL-6↓, iNOS↓, NO↓, MMP-2↓, MMP-3↓, MMP-9↓, NF- κB p65↓ (20 mg·kg^-1^); TNF-α↓, IL-6↓, iNOS↓, NO↓, MMP-2↓, MMP-3↓, MMP-9↓, NF- κB p65↓ (40 mg·kg^-1^). **5. Immunohistochemistry**: NF- κB p65↓ (20 mg·kg^-1^); NF- κB p65↓ (40 mg·kg^-1^). | 1. p < 0.01, 2. p < 0.01, 4. p < 0.01, 5. p < 0.01. |
| Kang (2007） | 20(S)-Rg3 | Rats (Wistar; male; NA; 120-130g) | 6, 6/6 | LPS | 5, 10 mg·kg^-1^ | Laboratory preparation | By intragastric/pretreatment 15 days | **1. Blood indicators**: NO^2-^/NO^3-^↔, ALT↓, AST↓, creatinine↓, urea nitrogen↔, TBA-reactive substance↓ (5 mg·kg^-1^); NO2-/NO3-↓, ALT↓, AST↓, creatinine↓, urea nitrogen↔, TBA-reactive substance↓ (10 mg·kg^-1^). **2. Liver tissue indicators**: TBA-reactive substance↔ (5 mg·kg^-1^); TBA-reactive substance↓ (10 mg·kg^-1^). **3. Protein expression**: p65↓, COX-2↔, iNOS↔, HO-1↑ (5 mg·kg^-1^); p65↓, COX-2↓, iNOS↓, HO-1↑ (10 mg·kg^-1^). | 1. p < 0.05 or p < 0.01, 2. p < 0.05, 3. p < 0.05 or p < 0.01. |
| Li (2020) | 20(R)-Rg3 | mice (ICR; male; NA; 20-25g) | 8, 8/8 | D-galactose | 10, 20 mg·kg^-1^ | Laboratory preparation | By intragastric/ 4 weeks | **1. Blood indicators**: ALT↓, SOD↑, MDA↓, CAT↑ (10 mg·kg^-1^); ALT↓, SOD↑, MDA↓, CAT↑ (20 mg·kg^-1^). 2. Liver Index↓ (10 mg·kg^-1^); Liver Index↓ (20 mg·kg^-1^). **3. HE Staining**. **4. Immunohistochemistry**: AGEs↓ (10 mg·kg^-1^); AGEs↓ (20 mg·kg^-1^). **5. Liver tissue indicators**: SOD↑, MDA↓, CAT↑ (10 mg·kg^-1^); SOD↑, MDA↓, CAT↑ (20 mg·kg^-1^). **6. Immunofuorescence**: 4-HNE↓, CYP2E1↓ (10 mg·kg^-1^); 4-HNE↓, CYP2E1↓ (20 mg·kg^-1^). **7. Protein expression**: p-PI3K↑, p-Akt↑, p53↓, Bax↓, Bcl-2↓, cleaved-caspase-3↓ (10 mg·kg^-1^); p-PI3K↑, p-Akt↑, p53↓, Bax↓, Bcl-2↑, cleaved-caspase-3↓ (20 mg·kg^-1^). | 1. p < 0.05 or p < 0.01, 2. p < 0.05, 4. p < 0.01, 5. p < 0.05 or p < 0.01, 6. p < 0.01, 7. p < 0.05 or p < 0.01. |
| Xiao (2018) | Rg1 | mice (C57BL/6; male; 6-8 weeks; 16±2g) | 10/10 | D-galactose | 20 mg·kg^-1^ | Tonghua Hongjiu Biotech Co., LTD. | By intraperitoneal injection/35 days | **1. Body weight**↔ (20 mg·kg^-1^). **2. Liver weight**↔ (20 mg·kg^-1^). **3. Liver indices**↓ (20 mg·kg^-1^). **4. Blood indicators**: ALT↓, AST↓, ALB↑, TBiL↓ (20 mg·kg^-1^). **5. HE Staining**. **6. TEM**. **7. SA‑β‑gal staining**: ROD↓ (20 mg·kg^-1^). **8. Liver tissue indicators**: SOD↑, MDA↓, GSH‑Px↑, GSH↑, 8‑OH‑dG↓, AGEs↔ (20 mg·kg^-1^). | 3. p < 0.05, 4. p < 0.05, 6. p < 0.05, 7. p < 0.05, 8. p < 0.05. |
| Liu (2021) | Rb1 | mice (C57BL/6; male; 8-10 weeks; 25±5g) | 10, 10/10 | D-galactose+LPS | 30, 60 mg·kg^-1^ | Dalian Meilun Pharmaceutical Co. LTD | By intraperitoneal injection/pretreatment 3 days | **1. Blood indicators**: ALT↓, AST↓, IL-6↓, TNF-α↓, IL-1β↓, IL-18↓ (30 mg·kg^-1^); ALT↓, AST↓, IL-6↓, TNF-α↓, IL-1β↓, IL-18↓ (60 mg·kg^-1^). **2. Hepatic index**↓ (30 mg·kg^-1^); Hepatic index↓ (60 mg·kg^-1^). **3. Histological score**↓ (30 mg·kg^-1^); Histological score↓ (60 mg·kg^-1^). **4. HE Staining**. **5. Liver tissue indicators**: IL-6↓, TNF-α↓, IL-1β↓, IL-18↓, ROS↓, MPO↓, MDA↓, SOD↑, GSH-px↑ (30 mg·kg^-1^); IL-6↓, TNF-α↓, IL-1β↓, IL-18↓, ROS↓, MPO↓, MDA↓, SOD↑, GSH-px↑ (60 mg·kg^-1^). **6. Immunohistochemistry**. **7. Gene expression**: IL-1β↓, IL-18↓, TLR4↓, MyD88↓ (30 mg·kg^-1^); IL-1β↓, IL-18↓, TLR4↓, MyD88↓ (60 mg·kg^-1^). **8. Protein expression**: NLRP3↓, ASC↓, caspase-1↓, TLR4↓, MyD88↓, p-IκBα↓, IκBα↓, nuc p65↓, cyto p65↑ (30 mg·kg^-1^); NLRP3↓, ASC↓, caspase-1↓, TLR4↓, MyD88↓, p-IκBα↓, IκBα↓, nuc p65↓, cyto p65↑ (60 mg·kg^-1^). | 1. p < 0.05, 2. p < 0.05, 3. p < 0.05, 5. p < 0.05, 7. p < 0.05, 8. p < 0.05. |
| Ning (2018) | Rg1 | mice (C57BL/6; male; 8 weeks; NA) | 10, 10, 10/10 | D-galactose+LPS | 15, 30, 60 mg·kg^-1^ | Chengdu Must Biotechnology Co., LTD | By intraperitoneal injection/pretreatment 3 days | **1. Blood indicators**: ALT↔, AST↔ (15 mg·kg^-1^); ALT↓, AST↓ (30 mg·kg^-1^); ALT↓, AST↓ (60 mg·kg^-1^). **2. Liver weight**↔ (15 mg·kg^-1^); Liver weight↓ (30 mg·kg^-1^); Liver weight↓ (60 mg·kg^-1^). **3. HE Staining**: necrosis score↔ (15 mg·kg^-1^); necrosis score↓ (30 mg·kg^-1^); necrosis score↓ (60 mg·kg^-1^). **4. Liver tissue indicators**: SOD↑, MDA↔, GSH↑, MPO↑, ROS↓ (15 mg·kg^-1^); SOD↑, MDA↓, GSH↑, MPO↑, ROS↓ (30 mg·kg^-1^); SOD↑, MDA↓, GSH↑, MPO↑, ROS↓ (60 mg·kg^-1^). **5. Gene expression**: TNF-α↓, IL-1β↓, IL-6↓, Mcp-1↓, Mip-2↓, iNOS↓, IL-10↓, Nrf2↑, Mrp2↑, GCLC↑, GCLM↑, HO-1↑, NQO1↑, TLR4↓, MD2↓, CD14↔ (15 mg·kg^-1^); TNF-α↓, IL-1β↓, IL-6↓, Mcp-1↓, Mip-2↓, iNOS↓, IL-10↓, Nrf2↑, Mrp2↑, GCLC↑, GCLM↑, HO-1↑, NQO1↑, TLR4↓, MD2↓, CD14↓ (60 mg·kg^-1^). **6. Immunohistochemistry**. **7. Protein expression**: TLR4↓, IκB↑, p-IκB↓, NF-κB p65↓, p-NF-κB p65↓, p-IRF3/IRF3↓, p-JNK/JNK↓, p-ERK/ERK↓, p-p38/P38↓ (15 mg·kg^-1^); TLR4↓, IκB↑, p-IκB↓, NF-κB p65↓, p-NF-κB p65↓, p-IRF3/IRF3↓, p-JNK/JNK↓, p-ERK/ERK↓, p-p38/P38↓ (60 mg·kg^-1^). | 1. p < 0.05, 2. p < 0.05, 3. p < 0.05, 4. p < 0.05, 5. p < 0.05, 6. p < 0.05, 7. p < 0.05. |
| Lee (2005) | 20(S)-Rg3 | mice (ICR; male; NA; 20-25g) | 5, 5/5 | tert-Butyl Hydroperoxide | 12.5, 25, 50 mg·kg^-1^ | Laboratory preparation | By intragastric/pretreatment 3 days; intraperitoneal injection/pretreatment once | **1. Blood indicators**: ALT↔, AST↓ (intragastric 25 mg·kg^-1^), ALT↔, AST↓ (intragastric 50 mg·kg^-1^), ALT↔, AST↔ (intraperitoneal injection 12.5 mg·kg^-1^), ALT↔, AST↔ (intraperitoneal injection 25 mg·kg^-1^). | 1. p < 0.05. |
|  | 20(S)-Rh2 | mice (ICR; male; NA; 20-25g) | 5, 5/5 | tert-Butyl Hydroperoxide | 12.5, 25, 50 mg·kg^-1^ | Laboratory preparation | By intragastric/pretreatment 3 days; intraperitoneal injection/pretreatment once | **1. Blood indicators**: ALT↓, AST↓ (intragastric 25 mg·kg^-1^), ALT↓, AST↓ (intragastric 50 mg·kg^-1^), ALT↓, AST↓ (intraperitoneal injection 12.5 mg·kg^-1^), ALT↓, AST↓ (intraperitoneal injection 25 mg·kg^-1^). | 1. p < 0.05. |
| Lee (2005) | Rb1 | mice (ICR; male; NA; 20-25g) | 5, 5/5 | tert-Butyl Hydroperoxide | 25, 50 mg·kg^-1^ | Laboratory preparation | By intragastric/pretreatment 3 days; intraperitoneal injection/pretreatment once | **1. Blood indicators**: ALT↓, AST↓ (intragastric 25 mg·kg-1), ALT↔, AST↔ (intraperitoneal injection 25 mg·kg-1), ALT↔, AST↔ (intraperitoneal injection 50 mg·kg-1). | 1. p < 0.05. |
|  | CK | mice (ICR; male; NA; 20-25g) | 5, 5/5 | tert-Butyl Hydroperoxide | 2, 10, 25, 50 mg·kg^-1^ | Laboratory preparation | By intragastric/pretreatment 3 days; intraperitoneal injection/pretreatment once | **1. Blood indicators**: ALT↓, AST↓ (intragastric 25 mg·kg^-1^), ALT↓, AST↓ (intragastric 50 mg·kg^-1^), ALT↔, AST↓ (intraperitoneal injection 2 mg·kg^-1^), ALT↓, AST↓ (intraperitoneal injection 10 mg·kg^-1^), ALT↓, AST↓ (intraperitoneal injection 25 mg·kg^-1^), ALT↔, AST↔ (intraperitoneal injection 50 mg·kg^-1^). | 1. p < 0.05. |
| Bi (2021) | Rg1 | mice (Kunming; female; 16 weeks; 22-25g) | 6, 6, 6/6 | APAP | 10, 20, 30 mg·kg^-1^ | Laboratory preparation | By intragastric/pretreatment 7 days | **1. Blood indicators**: AST↓, ALT↔ (10 mg·kg^-1^); AST↓, ALT↓ (20 mg·kg^-1^); AST↓, ALT↓ (30 mg·kg^-1^). **2. Liver tissue indicators**: MDA↓, GSH↔, SOD↔; TNF-α↔; IL-6↔; 1L-1β↔ (10 mg·kg^-1^); MDA↓, GSH↑, SOD↑; TNF-α↔; IL-6↔; 1L-1β↓ (20 mg·kg^-1^); MDA↓, GSH↑, SOD↑; TNF-α↔; IL-6↓; 1L-1β↓ (30 mg·kg^-1^); **3. HE Staining**: Histological Score↔ (10 mg·kg^-1^); Histological Score↓ (20 mg·kg^-1^); Histological Score↓ (30 mg·kg^-1^); **4. Protein expression**: BAX↔, BCL-2↑ (10 mg·kg-1); BAX↓, BCL-2↑ (20 mg·kg-1); BAX↓, BCL-2↑ (30 mg·kg^-1^). | 1. p < 0.05. 2. p < 0.05 or p < 0.01, 3. p < 0.05, 4. p < 0.05 |
|  | Rh1 | mice (Kunming; female; 16 weeks; 22-25g) | 6, 6, 6/6 | APAP | 10, 20, 30 mg·kg^-1^ | Laboratory preparation | By intragastric/pretreatment 7 days | **1. Blood indicators**: AST↓, ALT↔ (10 mg·kg^-1^); AST↓, ALT↓ (20 mg·kg^-1^); AST↓, ALT↓ (30 mg·kg^-1^). **2. Liver tissue indicators**: MDA↓, GSH↔, SOD↑; TNF-α↔; IL-6↔; 1L-1β↔ (10 mg·kg^-1^); MDA↓, GSH↑, SOD↑; TNF-α↓; IL-6↓; 1L-1β↓ (20 mg·kg^-1^); MDA↓, GSH↑, SOD↑; TNF-α↓; IL-6↓; 1L-1β↓ (30 mg·kg^-1^). **3. HE Staining**: Histological Score↔ (10 mg·kg^-1^); Histological Score↓ (20 mg·kg^-1^); Histological Score↓ (30 mg·kg^-1^). **4. Protein expression**: BAX↓, BCL-2↑ (10 mg·kg^-1^); BAX↓, BCL-2↔ (20 mg·kg^-1^); BAX↓, BCL-2↑ (30 mg·kg^-1^). | 1. p < 0.05. 2. p < 0.05 or p < 0.01, 3. p < 0.05, 4. p < 0.05 |
| Gao (2021) | Rg3 | mice (C57BL/6; male; 8 weeks; NA) | 10, 10/10 | APAP | 5, 10, 20 mg·kg^-1^ | the Chinese Academy of Medical Sciences and Peking Union Medical College | By intragastric/pretreatment 7 days | **1. Blood indicators**: ALT↓, AST↓, LDH↓, ALP↓ (5 mg·kg^-1^); ALT↓, AST↓, LDH↓, ALP↓ (10 mg·kg^-1^); ALT↓, AST↔, LDH↓, ALP↓ (20 mg·kg^-1^). **2. Liver tissue indicators**: MDA↓, T-AOC↑, GSH↑, GSH-PX↓ (5 mg·kg^-1^); MDA↓, T-AOC↑, GSH↑, GSH-PX↓ (10 mg·kg^-1^); MDA↓, T-AOC↔, GSH↑, GSH-PX↓ (20 mg·kg^-1^). **3. HE Staining**: necrotic scores↓ (5 mg·kg^-1^); necrotic scores↓ (10 mg·kg^-1^); necrotic scores↓ (20 mg·kg^-1^). **4. inflammatory factors**: TNF-α↔, CCL3↓, IL-1α↓, IL-1β↓, IL-2↔, IL-3↔, IL-4↔, IL-5↔, IL-6↓, IL-10↔, CCL4↔, CCL5↔, G-CSF↔, GM-CSF↔, GROα/KC↓, CCL11↔ (5 mg·kg^-1^); TNF-α↔, CCL3↓, IL-1α↔, IL-1β↓, IL-2↔, IL-3↔, IL-4↔, IL-5↓, IL-6↓, IL-10↔, CCL4↔, CCL5↔, G-CSF↔, GM-CSF↑, GROα/KC↔, CCL11↓ (10 mg·kg^-1^); TNF-α↓, CCL3↓, IL-1α↔, IL-1β↓, IL-2↔, IL-3↔, IL-4↔, IL-5↓, IL-6↓, IL-10↔, CCL4↔, CCL5↔, G-CSF↔, GM-CSF↑, GROα/KC↓, CCL11↓ (20 mg·kg^-1^). **5. Molecular docking**. | 1. p < 0.05 or p < 0.01 or p < 0.001, 2. p < 0.05 or p < 0.01 or p < 0.001, 3. p < 0.01 or p < 0.001,4. p < 0.05 or p < 0.01. |
| Qu (2021) | Rk3 | mice (ICR; male; 8 weeks; 20-22g) | 10, 10/10 | APAP | 25, 50 mg·kg-1 | Chengdu Puruifa Technology Co., LTD. | By intragastric/pretreatment 7 days | **1. HE Staining**: necrosis score↓ (25 mg·kg^-1^); necrosis score↓ (50 mg·kg^-1^). **2. Immunohistochemistry**. **3. Blood indicators**: TNF‑α↓, IL‑6↓, IL-1β↓ (25 mg·kg^-1^); TNF‑α↓, IL‑6↔, IL-1β↓ (50 mg·kg^-1^). **4. Gene expression**. **5. Protein expression**: TNF‑α↓, IL‑6↔, IL-1β↓, TLR4↓, NLRP3↓, Caspase 1↓, CYP2E1↓, HO-1↓, NQO1↑, Cyto Nrf2↔, Nuc Nrf2↑ (25 mg·kg^-1^); TNF‑α↓, IL‑6↓, IL-1β↓, TLR4↓, NLRP3↓, Caspase 1↓, CYP2E1↓, HO-1↓, NQO1↑, Cyto Nrf2↔, Nuc Nrf2↑ (50 mg·kg^-1^). **6. Liver tissue indicators**: SOD↑, MDA↔, ROS↓ (25 mg·kg-1); SOD↑, MDA↓, ROS↓ (50 mg·kg^-1^). **7. TSM.** | 1. p < 0.01 or p < 0.001, 3. p < 0.01 or p < 0.001, 5. p < 0.05 or p < 0.01, 5. p < 0.05 or p < 0.01 or p < 0.001, 6. p < 0.05 or p < 0.01 or p < 0.001. |
| Ning (2018) | Rg1 | mice (C57BL/6; male; 8-10 weeks; 20-25g) | 10, 10, 10/10 | APAP | 15, 30, 60 mg·kg^-1^ | Chengdu Must Biotechnology Co., LTD | By intragastric/pretreatment 3 days | **1. HE Staining**: necrosis score↔ (15 mg·kg^-1^); necrosis score↓ (30 mg·kg^-1^); necrosis score↓ (60 mg·kg^-1^). **2. Blood indicators**: ALT↓, AST↓, LDH↓ (15 mg·kg^-1^); ALT↓, AST↓, LDH↓ (30 mg·kg^-1^); ALT↓, AST↓, LDH↓ (60 mg·kg^-1^). **3. Liver tissue indicators**: SOD↔, MDA↔, GSH↑, CAT↑, (15 mg·kg^-1^); SOD↑, MDA↓, GSH↑, CAT↑, (30 mg·kg^-1^); SOD↑, MDA↓, GSH↑, CAT↑, (60 mg·kg^-1^). **4. Protein expression**: Cyto Nrf2↓, Nuc Nrf2↑, Keap1↓, Nrf2↑, GCLC↑, GCLM↑, HO-1↑, NQO1↑, Mrp2↑, Mrp3↑, Mrp4↑ (60 mg·kg^-1^). **5. Gene expression**: Ugt1a1↑, Ugt1a6↑, Ugt2b1↑, Sult2a1↑, CYP2E1↓, Cyp3a11↓, Cyp1a2↓ (60 mg·kg^-1^). | 1. p < 0.05, 2. p < 0.05, 3. p < 0.05, 4. p < 0.05, 5. p < 0.05. |
| Wang (2017) | Rg5 | mice (ICR; male; 8 weeks; 26-28g) | 8, 8/8 | APAP | 10, 20 mg·kg^-1^ | Laboratory preparation | By intragastric/pretreatment 7 days | **1. Blood indicators**: ALT↔, AST↓ (10 mg·kg^-1^); ALT↓, AST↓ (20 mg·kg^-1^). **2. immunoﬂuorescence staining**: 4-HNE↓, CYP2E1↓, iNOS↓, 3-NT↓ (10 mg·kg^-1^); 4-HNE↓, CYP2E1↓, iNOS↓, 3-NT↓ (20 mg·kg^-1^). **3. Liver tissue indicators**: GSH↑, MDA↓, GST↑, TNF-α↔, IL-1β↓ (10 mg·kg^-1^); GSH↑, MDA↓, GST↑, TNF-α↓, IL-1β↓ (20 mg·kg^-1^). **4. HE staining**: necrosis scores↓ (10 mg·kg^-1^); necrosis scores↓ (20 mg·kg^-1^). **5. Hoechst 33258 staining**: percentage of apoptosis↓ (10 mg·kg^-1^); percentage of apoptosis↓ (20 mg·kg^-1^). **6. immunohistochemical**: Bax↓, Bcl-2↑, PCNA↓ (10 mg·kg^-1^); Bax↓, Bcl-2↑, PCNA↓ (20 mg·kg^-1^). **7. Protein expression**: Bcl-2↔, Bax↓, Cytochrome C↓, Cleaved Caspase 3↓, Cleaved Caspase 8↓, Cleaved Caspase 9↓ (10 mg·kg^-1^); Bcl-2↓, Bax↓, Cytochrome C↓, Cleaved Caspase 3↓, Cleaved Caspase 8↓, Cleaved Caspase 9↓ (20 mg·kg^-1^). | 1. p < 0.05 or p < 0.01, 2. p < 0.05 or p < 0.01, 3. p < 0.05, 4. p < 0.05, 5. p < 0.05, 6. p < 0.05 or p < 0.01, 7. p < 0.05 or p < 0.01. |
| Ren (2019) | Rb1 | mice (ICR; male; 8-10 weeks; 20-22g) | 8, 8/8 | APAP | 10, 20 mg·kg^-1^ | Laboratory preparation | By intragastric/pretreatment 7 days | **1. Blood indicators**: ALT↓, AST↓, TNF-α↓, IL-1β↓ (10 mg·kg^-1^); ALT↓, AST↓, TNF-α↓, IL-1β↓ (20 mg·kg^-1^). **2. HE Staining**: necrosis score↓ (10 mg·kg^-1^); necrosis score↓ (20 mg·kg^-1^). **3. Liver tissue indicators**: GSH↑ (10 mg·kg^-1^); GSH↑ (20 mg·kg^-1^). **4. Protein expression**: TNF-α↓, IL-1β↓, iNOS↓,COX-2↓, p-IKKα/IKKα↓, p-IKKβ/IKKβ↓, p-IκB/IκB↓, p-NF-κB/NF-κB↓, p-JNK/JNK↓, p-ERK/ERK↓, p-p38/p38↓, PI3K/PI3K↑, Akt/Akt↑ (10 mg·kg^-1^); TNF-α↓, IL-1β↓, iNOS↓, COX-2↓, p-IKKα/IKKα↓, p-IKKβ/IKKβ↓, p-IκB/IκB↓, p-NF-κB/NF-κB↓, p-JNK/JNK↓, p-ERK/ERK↓, p-p38/p38↓, PI3K/PI3K↑, Akt/Akt↑ (20 mg·kg^-1^). **5. Immunoﬂuorescence**: TNF-α↓, IL-1β↓, iNOS↓, COX-2↓ (10 mg·kg^-1^); TNF-α↓, IL-1β↓, iNOS↓, COX-2↓ (20 mg·kg^-1^). | 1. p < 0.05 or p < 0.01, 2. p < 0.05 or p < 0.01, 3. p < 0.05 or p < 0.01, 4. p < 0.05 or p < 0.01, 5. p < 0.05 or p < 0.01. |
| Zhou (2018) | 20(R)-Rg3 | mice (ICR; male; NA; 20-25g) | 8/8 | APAP | 10, 20 mg·kg^-1^ | Laboratory preparation | By intragastric/pretreatment 7 days | **1. Blood indicators**: ALT↓, AST↓, TNF-α↓, IL-1β↓ (10 mg·kg^-1^); ALT↓, AST↓, TNF-α↓, IL-1β↓ (10 mg·kg^-1^). **2. Liver tissue indicators**: GSH↑, MDA↓ (10 mg·kg^-1^); GSH↑, MDA↓ (20 mg·kg^-1^). **3. Immunoﬂuorescence**: 4HNE↓, CYP2E1↓ (10 mg·kg^-1^); 4HNE↓, CYP2E1↓ (20 mg·kg^-1^). **4. HE Staining**: necrosis scroe↓ (10 mg·kg^-1^); necrosis scroe↓ (20 mg·kg^-1^). **5. TUNEL Staining**: apoptotic↓ (10 mg·kg^-1^); apoptotic↓ (20 mg·kg^-1^). **6. Hoechst 33,258 Staining**: apoptotic↓ (10 mg·kg^-1^); apoptotic↓ (20 mg·kg^-1^). **7. protein expression**: p-PI3K/PI3K↑, p-AKT/AKT↑, Bax↓, Bcl-2↑, p-IKKα/β/IKKα↓, p-IKKα/β/IKKβ↓, p-IκBα/IκBα↓, p-NF-κB/NF-κB↓ (10 mg·kg^-1^); p-PI3K/PI3K↑, p-AKT/AKT↑, Bax↓, Bcl-2↑, p-IKKα/β/IKKα↓, p-IKKα/β/IKKβ↓, p-IκBα/IκBα↓, p-NF-κB/NF-κB↓ (20 mg·kg^-1^). | 1. p < 0.05 or p < 0.01, 2. p < 0.05 or p < 0.01, 3. p < 0.05 or p < 0.01, 4. p < 0.05 or p < 0.01, 5. p < 0.05 or p < 0.01, 6. p < 0.05 or p < 0.01, 7. p < 0.05 or p < 0.01. |
| Gao (2017) | Rg1 | mice (C57BL/6; male; 16 weeks; 23-25g) | 10, 10, 10, 10, 10/10 | Cisplati | 20, 40, 80, 160, 320 mg·kg^-1^ | Utilized in powder form was obtained from Yunna Jecui Health Industry Corp. Ltd. | By intragastric/ (1) Rg1 and cisplatin co-treatment for 5 days. | **1. Blood indicators**: AST↔, ALT↔, ALP↔, LDH↔ (20 mg·kg^-1^); AST↔, ALT↔, ALP↔, LDH↔ (40 mg·kg^-1^); AST↔, ALT↔, ALP↓, LDH↔ (80 mg·kg^-1^); AST↔, ALT↔, ALP↔, LDH↔ (160 mg·kg^-1^); AST↔, ALT↔, ALP↓, LDH↔ (320 mg·kg^-1^). **2. HE Staining**. **3. Liver tissue indicators**: GSH↔, GSH-Px↔, T-AOC↑, MDA↔, MPO↔ (20 mg·kg^-1^); GSH↔, GSH-Px↑, T-AOC↑, MDA↓, MPO↔ (40 mg·kg^-1^); GSH↔, GSH-Px↔, T-AOC↔, MDA↓, MPO↔ (80 mg·kg^-1^); GSH↔, GSH-Px↔, T-AOC↑, MDA↔, MPO↔ (160 mg·kg^-1^); GSH↔, GSH-Px↔, T-AOC↑, MDA↔, MPO↑ (320 mg·kg^-1^). **4. DHE staining**: ROS↔ (20 mg·kg^-1^); ROS↔ (40 mg·kg^-1^); ROS↓ (80 mg·kg^-1^); ROS↓ (160 mg·kg^-1^); ROS↔ (320 mg·kg^-1^). **5. Protein expression**: cyto-Nrf2↓, nuc-Nrf2↓, p-Nrf2↓, cyto-Keap1↓, nuc-Keap1↓, HO-1↔, NQO1↔, GCLM↔, GCLC↔, p-JNK↓, p62↔ (20 mg·kg^-1^); cyto-Nrf2↓, nuc-Nrf2↔, p-Nrf2↓, cyto-Keap1↓, nuc-Keap1↓, HO-1↔, NQO1↑, GCLM↑, GCLC↔, p-JNK↓, p62↔ (40 mg·kg^-1^);cyto-Nrf2↓, nuc-Nrf2↔, p-Nrf2↓, cyto-Keap1↔, nuc-Keap1↓, HO-1↑, NQO1↑, GCLM↑, GCLC↔, p-JNK↓, p62↔ (80 mg·kg^-1^);cyto-Nrf2↑,nuc-Nrf2↑, p-Nrf2↑, cyto-Keap1↓, nuc-Keap1↓, HO-1↔, NQO1↔, GCLM↔, GCLC↔, p-JNK↓, p62↑ (160 mg·kg^-1^);cyto-Nrf2↔, nuc-Nrf2↑, p-Nrf2↔, cyto-Keap1↔, nuc-Keap1↓, HO-1↔, NQO1↔, GCLM↔, GCLC↔, p-JNK↓, p62↑ (320 mg·kg^-1^). **6. Co-IP**. | 1. p < 0.05 or p < 0.01, 3. p < 0.05 or p < 0.01, 4. p < 0.05, 5. p < 0.05 or p < 0.01. |
|  |  |  |  |  |  |  | (2) pretreatment 3 days, and then given both Rg1 and cisplatin as mentioned above for 5 days. | **1. Blood indicators**: AST↔, ALT↔, ALP↔, LDH↔ (20 mg·kg^-1^); AST↔, ALT↔, ALP↔, LDH↔ (40 mg·kg^-1^); AST↔, ALT↔, ALP↔, LDH↔ (80 mg·kg^-1^); AST↓, ALT↓, ALP↔, LDH↔ (160 mg·kg^-1^); AST↔, ALT↔, ALP↔, LDH↔ (320 mg·kg^-1^). **2.HE Staining**. **3. Liver tissue indicators**: GSH↔, GSH-Px↔, T-AOC↔, MDA↔, MPO↔ (20 mg·kg^-1^); GSH↑, GSH-Px↑, T-AOC↔, MDA↔, MPO↓ (40 mg·kg^-1^); GSH↑, GSH-Px↔, T-AOC↑, MDA↔, MPO↓ (80 mg·kg^-1^); GSH↔, GSH-Px↔, T-AOC↔, MDA↓, MPO↓ (160 mg·kg^-1^); GSH↑, GSH-Px↔, T-AOC↑, MDA↔, MPO↔ (320 mg·kg^-1^). **4. DHE staining**: ROS↓ (20 mg·kg^-1^); ROS↓ (40 mg·kg^-1^); ROS↓ (80 mg·kg^-1^); ROS↓ (160 mg·kg^-1^); ROS↓ (320 mg·kg^-1^). **5. Protein expression**: cyto-Nrf2↓, nuc-Nrf2↓, p-Nrf2↔, cyto-Keap1↔, nuc-Keap1↓, HO-1↔, NQO1↑, GCLM↓, GCLC↔, p-JNK↓, p62↓ (20 mg·kg^-1^);cyto-Nrf2↓, nuc-Nrf2↔, p-Nrf2↓, cyto-Keap1↓, nuc-Keap1↓, HO-1↔, NQO1↔, GCLM↓, GCLC↑, p-JNK↓, p62↓ (40 mg·kg^-1^); cyto-Nrf2↓, nuc-Nrf2↔, p-Nrf2↓, cyto-Keap1↔, nuc-Keap1↔, HO-1↔, NQO1↔, GCLM↓, GCLC↔, p-JNK↓, p62↓ (80 mg·kg^-1^);cyto-Nrf2↑, nuc-Nrf2↑, p-Nrf2↓, cyto-Keap1↔, nuc-Keap1↓, HO-1↔, NQO1↑, GCLM↓, GCLC↔, p-JNK↔, p62↓ (160 mg·kg^-1^); cyto-Nrf2↔, nuc-Nrf2↑, p-Nrf2↓, cyto-Keap1↔, nuc-Keap1↓, HO-1↔, NQO1↔, GCLM↓, GCLC↔, p-JNK↓, p62↓ (320 mg·kg^-1^). **6. Co-IP**. | 1. p < 0.05 or p < 0.01, 3. p < 0.05 or p < 0.01, 4. p < 0.05, 5. p < 0.05 or p < 0.01. |
| Zhou (2020) | CK | Rat (SD; male; 8-10 weeks; 200-250g) | 10, 10, 10/10 | sodium valproate | 80, 160, 320 mg·kg^-1^ | Zhejiang, Hisun Pharmaceutical Co., LTD. | By intragastric/15 days | **1. Liver index**↓ (80 mg·kg^-1^); Liver index ↓ (160 mg·kg^-1^); Liver index ↓ (320 mg·kg^-1^). **2. Blood indicators**: ALT↓, AST↔, ALP↓, ALB↔, hepcidin↑, iron↔ (80 mg·kg^-1^); ALT↓, AST↓, ALP↔, ALB↑, hepcidin↑, iron↔ (160 mg·kg**^-1^**); ALT↓, AST↓, ALP↓, ALB↑, hepcidin↑, iron↔ (320 mg·kg-1). **3. HE Staining**. **4. TEM**. **5. Liver tissue indicators**: TG↓, CAT↔, GSH-Px↑, SOD↑, GSH↑, MDA↓, HO-1↔, hepcidin↔, iron↓ (80 mg·kg**^-1^**); TG↓, CAT↑, GSH-Px↑, SOD↑, GSH↑, MDA↓, HO-1↔, hepcidin↑, iron↓ (160 mg·kg**^-1^**); TG↓, CAT↑, GSH-Px↑, SOD↑, GSH↑, MDA↓, HO-1↔, hepcidin↑, iron↓ (320 mg·kg**^-1^**). **6. Proteomic**. **7. Protein expression**: sEH↓ (80 mg·kg**^-1^**); sEH↔ (160 mg·kg**^-1^**); sEH↔ (320 mg·kg**^-1^**). | 1. p < 0.05 or p < 0.01, 2. p < 0.05 or p < 0.01 or p < 0.001 or p < 0.0001, 5. p < 0.05 or p < 0.01 or p < 0.001 or p < 0.0001, 7. p < 0.05. |
| Li (2018) | Rh1 | Rats (SD; male; NA; 180±20g) | 20/10 | alcohol＋Pyrazole | 60 mg·kg**^-1^** | Yunnan Yonuo Biological Engineering Co. LTD | By intragastric/3 weeks | **1. Blood indicators**: AST↓, ALT↓, DBiL ‬↔, TBiL ‬↔, GGT‬↔, ALP↑. **2. HE Staining.** | 1. p < 0.05 |
|  | CK | Rats (SD; male; NA; 180±20g) | 20/10 | alcohol＋Pyrazole | 60 mg·kg^-1^ | Yunnan Yonuo Biological Engineering Co. LTD | By intragastric/3 weeks | **1. Blood indicators**: AST↓, ALT↓, DBiL ‬↔, TBiL ‬↔, GGT‬↔, ALP↑. **2. HE Staining.** | 1. p < 0.05 |
| Zhang (2019) | CK | Rats (SD; male; NA; 280-300g) | 10, 10/10 | alcohol＋Pyrazole | 20, 40 mg·kg^-1^ | Shanghai Zhenzhun Bio Co. LTD | By intraperitoneal injection/3 weeks | **1. Protein expression**: GRP78 protein↓ (20 mg·kg^-1^); GRP78 protein↓ (40 mg·kg^-1^). **2. TG**↔ (20 mg·kg^-1^), TG↓ (40 mg·kg-^1^). **3. Hepatocyte apoptosis**↔ (20 mg·kg^-1^), Hepatocyte apoptosis↓ (40 mg·kg^-1^). **4. HE Staining**. | 1. p < 0.05. 2. p < 0.05, 3. p < 0.05 |
| Gao (2016) | Rg1 | mice (C57BL/6; male; 16 weeks; 23-25g) | 19, 19, 19/20 | alcohol | 10, 20, 40 mg·kg^-1^ | Utilized in powder form was obtained from Yunna Jecui Health Industry Corp. Ltd. | By intragastric/10 days | **1. Blood indicators**: ALT↓, AST↓, LDH↓, HDL↑, LDL↔, TG↔, ALP↔, CHO↔, GGT↔, T.Bili.↔ (10 mg·kg^-1^); ALT↔, AST↓, LDH↔, HDL↔, LDL↔, TG↔, ALP↓, CHO↔, GGT↔, T.Bili.↔ (20 mg·kg^-1^); ALT↔, AST↓, LDH↓, HDL↔, LDL↔, TG↔, ALP↓, CHO↓, GGT↔, T.Bili.↔ (40 mg·kg^-1^). **2. HE Staining**. **3. Gene expression**: LKB1↔, AMPKα2↔, AMPKγ1↔, PPARα↑, AMPKγ2↔, FAS↑ (10 mg·kg^-1^); LKB1↔, AMPKα2↑, AMPKγ1↔, PPARα↑, AMPKγ2↔, FAS↑ (20 mg·kg^-1^); LKB1↔, AMPKα2↑, AMPKγ1↑, PPARα↑, AMPKγ2↔, FAS↑ (40 mg·kg^-1^). **4. Protein expression**: LKB1 (S428)↓, LKB1 (T189)↔, AMPK (Thr172)↔, PPARα↔, CPT-1↔, SREBP-1↔,cyto-Nrf2↔, nuc-Nrf2↔, HO-1↔, NQO1↔ (10 mg·kg^-1^); LKB1 (S428)↔, LKB1 (T189)↔, AMPK (Thr172)↔, PPARα↔, CPT-1↔, SREBP-1↔, cyto-Nrf2↑, nuc-Nrf2↔, HO-1↔, NQO1↔ (20 mg·kg^-1^); LKB1 (S428)↔, LKB1 (T189)↔, AMPK (Thr172)↑, PPARα↔, CPT-1↔, SREBP-1↔, cyto-Nrf2↔, nuc-Nrf2↑, HO-1↑, NQO1↑ (40 mg·kg^-1^). | 1. p < 0.05 or p < 0.01 or p < 0.001, 3. p < 0.05 or p < 0.01 or p < 0.001, 4. p < 0.05 or p < 0.01. |
| Qu (2019) | Rk3 | mice (ICR; male; 6 weeks; 18-22g) | 10, 10/10 | alcohol | 25, 50 mg·kg^-1^ | Chengdu Puruifa Technology Co., LTD. | By intragastric/6 weeks | **1. Liver index**↓ (25 mg·kg^-1^); Liver index↓ (50 mg·kg^-1^). 2. Blood indicators: ALT↓, AST↓, TNF-α↔, IL-6↓, IL-1β↓ (25 mg·kg^-1^); ALT↓, AST↓, TNF-α↓, IL-6↓, IL-1β↓ (50 mg·kg^-1^); **3. Liver tissue indicators**: SOD↔, MDA↓, GSH↑ (25 mg·kg^-1^); SOD↑, MDA↓, GSH↑ (50 mg·kg^-1^). **4. Protein expression**: CYP2E1↔, NF-κB↓, TNF-α↔, IL-6↔, IL-1β↓, Bax↓, Bcl-2↑, cleaved caspase 3↔, cleaved caspase 8↔, cleaved caspase 9↔, cleaved PARP↔ (25 mg·kg^-1^); CYP2E1↓, NF-κB↓, TNF-α↓, IL-6↓, IL-1β↓, Bax↓, Bcl-2↑, cleaved caspase 3↓, cleaved caspase 8↓, cleaved caspase 9↓, cleaved PARP↓ (50 mg·kg^-1^). **5. HE Staining**. **6. Gene experssion**: TNF-α↓, IL-6↓, IL-1β↓ (25 mg·kg^-1^); TNF-α↓, IL-6↓, IL-1β↓ (50 mg·kg^-1^). **7. TUNEL staining.** | 1. p < 0.05, 2. p < 0.05, 3. p < 0.05, 4. p < 0.05, 6. p < 0.05, |
| Kim (2020） | F2 | mice (C57BL/6; male; 8 weeks; 24-26g) | 5/5 | alcohol | 50 mg·kg^-1^ | Laboratory preparation | By intragastric/14 days | **1. Blood indicators**: ALT↓, AST↔, ALB↔ (50 mg·kg^-1^). **2. HE Staining**: inﬂammatory foci↓, F4/80+↓, Gr1+↓ (50 mg·kg^-1^). **3. Flow cytometry**: F4/80^+^CD11b^+^ cells, Gr1^+^CD11b^+^ cells, CD4^+^ T cells, CD8^+^ T cells, NK cells, NKT cells, Tregs cells, Th17 cells (50 mg·kg^-1^). **4. Gene expression**: TNF-α↓, IL-6↔, IL-17a↓, IL-10↑, Foxp3↑, Tgfb1↔ (50 mg·kg^-1^). | 1. p < 0.05, 2. p < 0.01, 3. p < 0.05 or p < 0.01, 4. p < 0.05. |
| Lai (2021) | Rb1 | Zebraﬁsh (wild-type, lfabp10α: EGFP and MPO: EGFP, NA, 4 days NA) | 6-10, 6-10, 6-10/6-10 | alcohol | 6.25, 12.5, 25 μM | Chengdu Must Biotechnology Co., Ltd. | Soluble in fish water/ 48h | **1. HE Staining. 2. Oil Red O Staining**: liver gray value↓ (6.25 μM); liver gray value↓, area of lipid droplets↓ (12.5 μM); liver gray value↔ (25 μM). **3. Nile Red staining**: mean ﬂuorescence intensity (12.5 μM). **4. ﬂuorogenic probe**: ROS↓, GSH↑ (12.5 μM). **5. Immunoﬂuorescence**: number of neutrophils↓ (12.5 μM). **6. Immunohistochemical staining**: NF-κB↓, TNF-α↓ (12.5 μM). **7. Gene expression**: NF-κB↓, TNF-α↓ (12.5 μM). | 2. p < 0.001, 3. p < 0.001, 4. p < 0.01 or p < 0.001, 5. p < 0.001, 6. p < 0.001, 7. p < 0.001. |
| Yang (2021) | Rg1 | mice (C57BL/6; female; 8-10 weeks; NA) | 3-5/3-5 | alcohol | 40 mg·kg^-1^ | Yunnan Jicui Biological Engineering Co. LTD | By intraperitoneal injection/2 days | **1. Blood indicators**: ALT↓, AST↓, TBiL↓, IL-1β↔, IL-18↓ (40 mg·kg^-1^). **2. HE Staining**. **3. Flowcytometry**: neutrophils↓ (40 mg·kg^-1^). **4. Liver tissue indicators**: ADH↔ (40 mg·kg^-1^). **5. Gene expression**: CYP2E1↓ (40 mg·kg^-1^). **6. Protein expression**: CYP2E1↓, NLRP3↓, ASC↓, pro caspase-1↓, caspase-1↓ (40 mg·kg^-1^). **7. Immunohistochemistry staining**. 8. GSH-Px↑, MDA↓ (40 mg·kg^-1^). **9. TUNEL staining**. | 1. p < 0.01 or p < 0.001, 3. p < 0.01, 5. p < 0.001, 6. p < 0.001, 8. p < 0.01. |
| Yang (2015) | Rg1 | mice (Kunming; male; NA; 18-22g) | 10, 10, 10/10 | concanavalin A | 15, 30, 60 mg·kg^-1^ | Shanghai Ronghe Medical Technology Development Co., LTD | By intragastric/pretreatment 15 days | **1. Blood indicators**: ALT↔, AST↓, TNF-α↓, IFN-γ↓ (15 mg·kg^-1^); ALT↓, AST↓, TNF-α↓, IFN-γ↓ (30 mg·kg^-1^); ALT↓, AST↓, TNF-α↓, IFN-γ↓ 60 mg·kg^-1^). **2. HE Staining.** | 1. p < 0.01 or p < 0.05. |
| Chen (2020) | Rg1 | Rats（SD; NA; NA; NA) | 15/15 | high sugar and high fat feeding+streptozocin | 10 mg·kg^-1^ | NA | By intragastric/8 weeks | **1. Blood indicators**: FBG↓, AST↓, ALT↓ MDA↓, SOD↑, CAT↑, GSH-Px↑, T-AOC↑. **2. Immunoﬂuorescence Staining**: TLR4 protein↓. **3. HE Staining**. | 1. p < 0.05. 2. p < 0.05 |
| Li (2015) | Rg1 | Rats（SD; NA; NA; 200-220g) | 10, 10, 10/10 | high sugar and high fat feeding+streptozocin | 2, 4, 8 mg·kg^-1^ | Baoji Chenguang Biotechnology Co. LTD | By intragastric/8 weeks | **1. Blood indicators**: FBG↔, AST↔, ALT↓ MDA↔, SOD↔, TNF-α↔, IL-6↔ (2 mg·kg^-1^); FBG↓, AST↓, ALT↓ MDA↓, SOD↑, TNF-α↓, IL-6↓ (4 mg·kg^-1^); FBG↓, AST↓, ALT↓ MDA↓, SOD↑, TNF-α↓, IL-6↓ (8 mg·kg^-1^). **2. HE Staining**. | 1. p < 0.01 |
| Yan (2020) | CK | Rats (SD; male; NA; 200±20g) | 10, 10/10 | high sugar and high fat feeding+streptozocin | 5.25, 10.5 mg·kg^-1^ | Shanghai Yuanye Biotechnology Co., LTD. | By intragastric/8 weeks | **1. Blood indicators**: TC↓, TG↓, LDL↓, HDL↓, ALT↓, AST↓ (5.25 mg·kg^-1^); TC↓, TG↓; LDL↓, HDL↓, ALT↓, AST↓ (10.5 mg·kg^-1^). **2. Liver tissue indicators**: MDA↓, SOD↑, CAT↑, GSH↑, TNF-α↓, IL-6↓, IFN-γ↓, IL-1β↓ (5.25 mg·kg^-1^); MDA↓, SOD↑, CAT↑, GSH↑, TNF-α↓, IL-6↓, IFN-γ↓, IL-1β↓ (10.5 mg·kg^-1^). **3. Protein expression**: TLR4↓, Myd88↓, p-65↓, p- IKB-α3↓ (5.25 mg·kg^-1^); TLR4↓, Myd88↓, p-65↓, p- IKB-α3↓ (10.5 mg·kg^-1^). **4. HE Staining**. | 1. p < 0.01 or p < 0.05. 2. p < 0.01 or p < 0.05 |
| Tian (2017) | Rg1 | Rat (SD; male; 4 weeks; 200±20g) | 20, 20/20 | high sugar and high fat feeding+streptozocin | 25, 50 mg·kg^-1^ | the College of Pharmacy of Kunming Medical University | By intragastric/4 or 8 weeks | **1. HE Staining**. **2. Blood indicators**: Glu↓, insulin↔, IRI↓, ALT↓, AST↓, TC↓, TG↓, LDL↓, IL-1↓, IL-6↓, TNF-α↓ (25 mg·kg^-1^, 4 weeks); Glu↓, insulin↔, IRI↓, ALT↓, AST↓, TC↓, TG↓, LDL↓,IL-1↓, IL-6↓, TNF-α↓ (25 mg·kg^-1^, 8 weeks); Glu↓, insulin↔, IRI↓, ALT↓, AST↓, TC↓, TG↓, LDL↓,IL-1↓, IL-6↓, TNF-α↓ (50 mg·kg^-1^, 4 weeks); Glu↓, insulin↔, IRI↓, ALT↓, AST↓, TC↓, TG↓, LDL↓,IL-1↓, IL-6↓, TNF-α↓ (50 mg·kg^-1^, 8 weeks). **3. Protein expression**: p-JNK↓, JNK↔, caspase-3↓, Bcl-2↑, Bax↓ (25 mg·kg^-1^, 4 weeks); p-JNK↓, JNK↔, caspase-3↓, Bcl-2↑, Bax↓ (25 mg·kg^-1^, 8 weeks); p-JNK↓, JNK↔, caspase-3↓, Bcl-2↑, Bax↓ (50 mg·kg^-1^, 4 weeks); p-JNK↓, JNK↔, caspase-3↓, Bcl-2↑, Bax↓ (50 mg·kg^-1^, 8 weeks). **4. Gene expression**: JNK↔, caspase-3↓, Bcl-2↑, Bax↓ (25 mg·kg^-1^, 4 weeks); JNK↔, caspase-3↓, Bcl-2↑, Bax↓ (25 mg·kg^-1^, 8 weeks); JNK↔, caspase-3↓, Bcl-2↑, Bax↓ (50 mg·kg^-1^, 4 weeks); JNK↔, caspase-3↓, Bcl-2↑, Bax↓ (50 mg·kg^-1^, 8 weeks). | 2. p < 0.05, 3. p < 0.05, 4. p < 0.05. |
| Yao (2016) | Rg1 | Rats (NA; NA; NA; NA) | 12, 12, 12/12 | high sugar and high fat feeding | 2, 4, 8 mg·kg^-1^ | NA | By intragastric/8 weeks | **1. Blood indicators**: FBG↔, ALT↔, AST↔, MDA↔, SOD↔, TNF-α↔, IL-6↔ (2 mg·kg^-1^); FBG↓, ALT↓, AST↓, MDA↓, SOD↑, TNF-α↓, IL-6↓ (4 mg·kg^-1^); FBG↓, ALT↓, AST↓, MDA↓, SOD↑, TNF-α↓, IL-6↓ (8 mg·kg^-1^). | 1. p < 0.05 |
| Jiang (2021) | Rg3 | mice (db/db; male; 8 weeks; NA) | 8/8 | Spontaneous | 30 mg·kg^-1^ | Jilin Yatai Pharmaceutical Co., Ltd. | By intragastric/8 weeks | **1. Weight**↔ (30 mg·kg^-1^). **2. Blood indicators**: TG↔, TC↔, HDL↔, LDL↔, ALT↓, AST↓ (30 mg·kg-1). **3. HE Staining**. **4. Immunohistochemical**: PPARγ↑, TGF-β1↓, CTGF↓ (30 mg·kg-1). **5. Liver tissue indicators**: ACE↓, Ang II↓ (30 mg·kg-1). **6. Gene expression**: TNF-α↓, IL-6↓, Col-­I↓, Col-­III↓ (30 mg·kg^-1^). | 1. p < 0.05, 2. p < 0.05, 4. p < 0.05, 5. p < 0.05, 6. p < 0.05. |
| Liu (2014) | Rb1 | Rats (SD; male; 8-10 weeks; 180-220g) | 10, 10, 10/10 | Ischemia reperfusion | 20, 40 mg·kg^-1^ | National Institutes for Food and Drug Control | By vein injection/pretreatment once | **1. Blood indicators**: AST↓, ALT↓ MDA↓, SOD↑ (20 mg·kg^-1^); AST↔, ALT↔ (40 mg·kg^-1^). **2. HE Staining.** | 1. p < 0.05 |
| Lin (2020) | Rg1 | Rats (SD; male; NA; 250-300g) | 8/8 | Ischemia reperfusion | 20 mg·kg^-1^ | Shandong Xiya Chemical Technology Co., LTD | By tail vein injection/pretreatment once | **1. Blood indicators**: ALT↓, AST↓ (20 mg·kg^-1^). **2. HE Staining**. **3. Pathological score**↓ (20 mg·kg^-1^). **3. TUNEL staining**: apoptosis index↓ (20 mg·kg^-1^). **4. Protein expression**: cleaved caspase-3↓, cleaved Caspase-9↓, Cyt-C↓, CypD↓ (20 mg·kg^-1^). **5. Gene expression**: cleaved caspase-3↓, cleaved Caspase-9↓, Cyt-C↓, CypD↓ (20 mg·kg^-1^). **6. Flow cytometry**: MMP↑ (20 mg·kg-1). **7. TEM.** | 1. p < 0.05, 3. p < 0.05, 4. p < 0.01, 4. p < 0.05, 5. p < 0.05, 6. p < 0.05. |
| Zhang (2021) | Rg1 | mice (C57BL/6; male; 7-8 weeks; NA) | 6/6 | Ischemia reperfusion | 30 mg·kg^-1^ | NA | By tail vein injection/once | **1. Liver tissue indicators**: ROS↓, JC-1↑, ATP↑, ALT↓ (30 mg·kg^-1^). **2. Gene expression**: Erα↑, Yap↑ (30 mg·kg^-1^). **3. HE Staining**. | 1. p < 0.0001, 2. p < 0.0001. |
| Zhang (2015) | Rg1 | mice (C57BL/6; male; 6-8 weeks; 22-25 g) | 10/10 | Ischemia reperfusion | 20 mg·kg^-1^ | Greenherbs Science and Technology Development CO., LTD. | By intraperitoneal injection/pretreatment 7 days | **1. Blood indicators**: ALT (0, 6, 12, 24h) ↓, AST (0, 6, 12, 24h) ↓ (30 mg·kg-1). **2. HE Staining**: necrotic areas (6, 12, 24h) ↓ (30 mg·kg^-1^). **3. TUNEL staining**: Apoptotic(12h) ↓, MAC-1(0, 6, 12, 24h) ↓, Ly6G (6, 12, 24h) ↓, CD3(0, 6, 12, 24h) ↓ (30 mg·kg-1). **4. Gene expression**: Bcl-2(12h) ↔, Bax(12h) ↓, TNF-α(12h) ↓, IL-1β(12h) ↓, IL-6(12h) ↓, MCP-1(12h) ↓, CXCL2(12h) ↓ (30 mg·kg^-1^). **5. Protein expression**: Bcl-2(12h) ↓, Bax(12h) ↓, P-p65(12h) ↓ (30 mg·kg^-1^). | 1. p < 0.01 or p < 0.001, 2. p < 0.001, 3. p < 0.05 or p < 0.001, 4. p < 0.001, 5. p < 0.001. |
| Wang (2008) | Rb1 | Rat (Wistar; male; NA; 220-250g) | 10, 10/10 | Ischemia reperfusion | 20, 40 mg·kg^-1^ | the Research Center of Traditional Chinese Medicine, Wuhan | By intraperitoneal injection/pretreatment once | **1. HE Staining**: necrosis scroe↓ (20 mg·kg^-1^); necrosis scroe↓ (40 mg·kg^-1^). **2. Liver tissue indicators**: MDA↓, TNF-α↓, MPO↓ (20 mg·kg^-1^); MDA↓, TNF-α↓, MPO↓ (40 mg·kg^-1^). **3. Blood indicators**: ALT↓, AST↓ (20 mg·kg^-1^); ALT↓, AST↓ (40 mg·kg^-1^). **4. Immunohistochemical staining**: ICAM-1↓, NF-κB↓ (20 mg·kg^-1^); ICAM-1↓, NF-κB↓ (40 mg·kg^-1^). **5. protein expression**: NF-κB↓ (20 mg·kg^-1^); NF-κB↓ (40 mg·kg^-1^). | 1. p < 0.05, 2. p < 0.05, 3. p < 0.05, 4. p < 0.05, 5. p < 0.05. |
| Tiao (2014) | Rg1 | mice (C57BL/6; male; 6-8 weeks; 22-30g) | 6/6 | Ischemia reperfusion | 20 mg·kg^-1^ | Sigma-Aldrich Chemical Co. LTD | By intraperitoneal injection/pretreatment once | **1. HE Staining**: necrosis scroe↓ (20 mg·kg^-1^). **2. Blood indicators**: ALT↓, AST↓ (20 mg·kg^-1^). **3. Gene expression**: IL-1β↓, TNF-a↓, IL-6↓, IFN-γ↓, ICAM-1↓, CXCL-10↓ (20 mg·kg^-1^). **4. protein expression**. **5. TUNEL Staining**: apoptosis↓ (20 mg·kg^-1^). | 1. p < 0.01, 2. p < 0.05, 3. p < 0.05 or p < 0.01 or p < 0.001. 5. p < 0.05. |
| Wang (2022) | Mc1 | Rat (Wistar; male; 20-24 months; 300±25g) | 6/6 | high sugar and high fat feeding+streptozocin+Ischemia reperfusion | 10 mg·kg^-1^ | NA | By intraperitoneal injection/pretreatment 4 weeks | **1. Blood indicators**: ALT↓, AST↓ (10 mg·kg^-1^). **2. HE Staining**: necrosis score↓ (10 mg·kg^-1^). **3. Protein expression**: cleaved caspase-3↓, Bcl-2↔, Bax↓, AMPK↔, p-AMPK↑ (10 mg·kg^-1^). **4. Liver tissue indicators**: ROS↓, SOD↑, GSH↑, 8-isoprostane↔ (10 mg·kg^-1^). | 1. p < 0.05, 2. p < 0.05, 3. p < 0.05, 4. p < 0.05. |
| Wu (2021) | Rg3 | mice (C57BL/6; male; 7 weeks; 23–25g) | 6/6 | Caecal ligation and puncture | 20 mg·kg^-1^ | Sigma-Aldrich Chemical Co. LTD | By intraperitoneal injection/pretreatment once | **1. HE Staining**. **2. protein expression**: complex I↑, complex II↑, OPA1↑ (20 mg·kg^-1^). **3. Gene expression**: TUG1↑ (20 mg·kg^-1^). | 2. p < 0.05, 3. p < 0.01. |

**Table 1.** Summary of included studies. ALT: Alanine aminotransferase; AST: Aspartate aminotransferase; SOD: superoxide dismutase; MDA: malondialdehyde; GSH: glutathione; GSH-Px: glutathione peroxidase; CAT: catalase; HA: hyaluronic acid; PCⅢ: procollagen Ⅲ; TGF-β: transforming growth factor β; IL-6: Interleukin-6; IL-8: Interleukin-8; ALP: alkaline phosphatase; TNF-α: tumor necrosis factor-α; IL-1β: Interleukin-1β; COX-2: cyclooxygenase-2; iNOS: inducible nitric oxide synthase; Keap1: kelch-like ECH associating protein 1; Nrf2: Nuclear factor E2-related factor 2; NQO1: NAD(P)H quinone oxidoreductase 1; GCLM: glutamate-cysteine ligase modifier subunit; Bsep: bile salt export pump; Mrp2: multidrug resistance-associated protein 2; Mrp3: multidrug resistance-associated protein 3; Mrp4: multidrug resistance-associated protein 4; IL-18: Interleukin-18; MPO: myeloperoxidase; MMP-2: matrix metalloproteinase 2; MMP-3: matrix metalloproteinase 3; MMP-9: matrix metalloproteinase 9; COX-2l: cyclooxygenase-2; HO-1: heme oxygenase-1; AGEs: advanced glycation end products; 4-HNE: 4-hydroxynonenal; CYP2E1: cytochrome P450 E1; ALB: albumin; TBiL: total bilirubin; TEM: transmission electron microscopy; ROD: relative optical density; ROS: reactive oxygen species; TLR4: toll-like receptor 4; MyD88: myeloid differentiation factor 88; NF-κB: nuclear factor-kappa B; ASC: apoptosis associated speck-like protein containing CARD; Mcp-1: monocyte chemotactic protein-1; Mip-2: macrophage inflammatory protein-2; GCLC: glutamate-cysteine ligase catalytic; GCLM: glutamate-cysteine ligase modifier; MD2: myeloid differentiation factor 2; CD14: cluster of differentiation 14; LDH: lactate dehydrogenase; T-AOC: total antioxidative capacity; PCNA: proliferating cell nuclear antigen; 4-HNE: 4-hydroxynonenal; DBiL: direct bilirubin; GGT: γ-glutamyl transpeptidase; TG: triglyceride; CHO: cholesterol; HDL: high density lipoprotein; LDL: low density lipoprotein; LKB1: tumor suppressor liver kinase 1; AMPK: AMP-activated protein kinase; Th17: IL-17eproducing T; IL-17a: Interleukin-17a; IL-10: Interleukin-10; ADH: alcohol dehydrogenase; ASC: apoptosis-associated speck like CARD-domain containing protein; IFN-γ: interferon-γ; FBG: fasting plasma glucose/fasting blood glucose; IRI: insulin resistance index; Glu: glucose; IL-1: Interleukin-1; JNK: c-Jun N-terminal kinase; BCL-2: B cell leukemia/lymphoma 2; BAX: BCL-2-associated X protein; TC: total cholesterol; ACE: Angiotensin converting enzyme; Ang II: angiotensin II; Col-I: procollagen I; Col-III: procollagen III; TGF-β: transforming growth factor β; CTGF: connective tissue growth factor; CypD: Cyclophilin D; ATP: adenosine triphosphate; Erα: estrogen receptor α; YAP: Yes-associated protein; ICAM-1: intercellular adhesion molecule-1; OPA1: Mitochondrial Dynamin Like GTPase; TUG1: taurine-upregulated gene 1.
