# Supplementary material for: The effect of ginsenosides on liver injury in preclinical studies: a systematic review and meta-analysis
Source: Front Pharmacol. 2023 May 11;14:1184774. doi: 10.3389/fphar.2023.1184774 (PMC10213882; doi:10.3389/fphar.2023.1184774)
Supplement: Supplementary file 2 [file DataSheet1.docx]

**The effect of ginsenosides on liver injury in preclinical studies: A systematic review and meta-analysis**

Xing-Bo Bian ^1^, Peng-Cheng Yu ^2^, Xiao-Hang Yang ^1^, Qi-Yao Wang ^1^, Liu Han ^1^, Li Zhang ^1^, Lian-Xue Zhang ^3^, and Xin Sun ^1^*

1. College of pharmacy, Jilin Medical University, Jilin, 132013, Jilin Province, China
2. Heilongjiang University of Chinese Medicine, Harbin, 150006, Heilongjiang Province, China
3. College of Chinese Medicinal Materials, Jilin Agriculture University, Changchun, 130118, Jilin, China

* Corresponding author at: Jilin Medical University, Jilin, China. sxjlmu@163.com (X. Sun).

**The search strategy of the PubMed database:**

| ("Ginsenosides"[MeSH Terms] OR "Panaxosides"[Title/Abstract] OR "Ginsenoside"[Title/Abstract]) AND ("Chemical and Drug Induced Liver Injury"[MeSH Terms] OR ("Chemical"[Title/Abstract] AND "drug induced liver injury"[Title/Abstract]) OR "chemically induced liver toxicity"[Title/Abstract] OR "chemically induced liver toxicity"[Title/Abstract] OR (("Chemically-Induced"[MeSH Subheading] OR ("Chemically"[All Fields] AND "Induced"[All Fields]) OR "Chemically-Induced"[All Fields]) AND "liver toxicities"[Title/Abstract]) OR ((("Liver"[MeSH Terms] OR "Liver"[All Fields] OR "livers"[All Fields] OR "liver s"[All Fields]) AND ("Toxic"[All Fields] OR "toxical"[All Fields] OR "toxically"[All Fields] OR "toxicant"[All Fields] OR "toxicant s"[All Fields] OR "toxicants"[All Fields] OR "toxicated"[All Fields] OR "toxication"[All Fields] OR "Toxicities"[All Fields] OR "Toxicity"[MeSH Subheading] OR "Toxicity"[All Fields] OR "toxicity s"[All Fields] OR "toxics"[All Fields])) AND "Chemically-Induced"[Title/Abstract]) OR (("Liver"[MeSH Terms] OR "Liver"[All Fields] OR "livers"[All Fields] OR "liver s"[All Fields]) AND "toxicity chemically induced"[Title/Abstract]) OR (("Toxic"[All Fields] OR "toxical"[All Fields] OR "toxically"[All Fields] OR "toxicant"[All Fields] OR "toxicant s"[All Fields] OR "toxicants"[All Fields] OR "toxicated"[All Fields] OR "toxication"[All Fields] OR "Toxicities"[All Fields] OR "Toxicity"[MeSH Subheading] OR "Toxicity"[All Fields] OR "toxicity s"[All Fields] OR "toxics"[All Fields]) AND "chemically induced liver"[Title/Abstract]) OR (("Toxic"[All Fields] OR "toxical"[All Fields] OR "toxically"[All Fields] OR "toxicant"[All Fields] OR "toxicant s"[All Fields] OR "toxicants"[All Fields] OR "toxicated"[All Fields] OR "toxication"[All Fields] OR "Toxicities"[All Fields] OR "Toxicity"[MeSH Subheading] OR "Toxicity"[All Fields] OR "toxicity s"[All Fields] OR "toxics"[All Fields]) AND "chemically induced liver"[Title/Abstract]) OR "drug induced acute liver injury"[Title/Abstract] OR "drug induced acute liver injury"[Title/Abstract] OR "liver injury drug induced acute"[Title/Abstract] OR "acute liver injury drug induced"[Title/Abstract] OR "acute liver injury drug induced"[Title/Abstract] OR "hepatitis toxic"[Title/Abstract] OR "toxic hepatitis"[Title/Abstract] OR (("Hepatitis"[MeSH Terms] OR "Hepatitis"[All Fields] OR "Hepatitides"[All Fields] OR "hepatitis a"[MeSH Terms] OR "hepatitis a"[All Fields]) AND "Toxic"[Title/Abstract]) OR "toxic hepatitides"[Title/Abstract] OR "drug induced liver disease"[Title/Abstract] OR "disease drug induced liver"[Title/Abstract] OR "diseases drug induced liver"[Title/Abstract] OR "drug induced liver disease"[Title/Abstract] OR "drug induced liver diseases"[Title/Abstract] OR "liver disease drug induced"[Title/Abstract] OR "liver diseases drug induced"[Title/Abstract] OR "drug induced liver injury"[Title/Abstract] OR "drug induced liver injury"[Title/Abstract] OR "drug induced liver injuries"[Title/Abstract] OR "injuries drug induced liver"[Title/Abstract] OR "injury drug induced liver"[Title/Abstract] OR "liver injuries drug induced"[Title/Abstract] OR "liver injury drug induced"[Title/Abstract] OR "liver injury drug induced"[Title/Abstract] OR "hepatitis drug induced"[Title/Abstract] OR "drug induced hepatitides"[Title/Abstract] OR "drug induced hepatitis"[Title/Abstract] OR (("Hepatitis"[MeSH Terms] OR "Hepatitis"[All Fields] OR "Hepatitides"[All Fields] OR "hepatitis a"[MeSH Terms] OR "hepatitis a"[All Fields]) AND "Drug-Induced"[Title/Abstract]) OR "hepatitis drug induced"[Title/Abstract] OR "Hepatotoxicity"[Title/Abstract] OR "hepatic injury"[Title/Abstract] OR "liver damage"[Title/Abstract] OR "liver injury"[Title/Abstract]) |
| --- |

**Table S1.** The methodological quality of included studies.

| **Study** | **1** | **2** | **3** | **4** | **5** | **6** | **7** | **8** | **9** | **10** | **Total score** |
| --- | --- | --- | --- | --- | --- | --- | --- | --- | --- | --- | --- |
| Qi (2016) | ? | ? | ? | ? | ? | ? | ? | Y | Y | ? | 2 |
| Zhang (2006) | ? | ? | ? | ? | ? | ? | ? | Y | Y | ? | 2 |
| Li (2011) | ? | ? | ? | Y | ? | ? | ? | Y | Y | ? | 3 |
| Lu (2018) | ? | ? | ? | ? | ? | ? | ? | Y | Y | ? | 2 |
| Ning (2018) | ? | ? | ? | Y | ? | ? | ? | Y | Y | ? | 3 |
| Qi (2017) | ? | ? | ? | Y | ? | ? | ? | Y | Y | ? | 3 |
| Zhao (2021) | ? | ? | ? | Y | ? | ? | ? | Y | Y | ? | 3 |
| Yao (2016) | ? | ? | ? | Y | ? | ? | ? | Y | Y | ? | 3 |
| Kang (2007） | ? | ? | ? | ? | ? | ? | ? | Y | Y | ? | 2 |
| Li (2020) | ? | ? | ? | Y | ? | ? | ? | Y | Y | ? | 3 |
| Xiao (2018) | ? | ? | ? | Y | ? | ? | ? | Y | Y | ? | 3 |
| Liu (2021) | ? | ? | ? | Y | ? | ? | ? | Y | Y | ? | 3 |
| Ning (2018) | ? | ? | ? | Y | ? | ? | ? | Y | Y | ? | 3 |
| Lee (2005) | ? | ? | ? | ? | ? | ? | ? | Y | Y | ? | 2 |
| Lee (2005) | ? | ? | ? | ? | ? | ? | ? | Y | Y | ? | 2 |
| Bi (2021) | ? | ? | ? | ? | ? | ? | ? | Y | Y | ? | 2 |
| Gao (2021) | ? | ? | ? | Y | ? | ? | ? | Y | Y | ? | 3 |
| Qu (2021) | ? | ? | ? | Y | ? | ? | ? | Y | Y | ? | 3 |
| Ning (2018) | ? | ? | ? | Y | ? | ? | ? | Y | Y | ? | 3 |
| Wang (2017) | ? | ? | ? | Y | ? | ? | ? | Y | Y | ? | 3 |
| Ren (2019) | ? | ? | ? | Y | ? | ? | ? | Y | Y | ? | 3 |
| Zhou (2018) | ? | ? | ? | Y | ? | ? | ? | Y | Y | ? | 3 |
| Gao (2017) | ? | ? | ? | Y | ? | ? | ? | Y | Y | ? | 3 |
| Zhou (2020) | ? | ? | ? | Y | ? | ? | ? | Y | Y | ? | 3 |
| Li (2018) | ? | ? | ? | ? | ? | ? | ? | Y | Y | ? | 2 |
| Zhang (2019) | ? | ? | ? | ? | ? | ? | ? | Y | Y | ? | 2 |
| Gao (2016) | ? | ? | ? | Y | ? | ? | ? | Y | Y | ? | 3 |
| Qu (2019) | ? | ? | ? | Y | ? | ? | ? | Y | Y | ? | 3 |
| Kim (2020 | ? | ? | ? | ? | ? | ? | ? | Y | Y | ? | 2 |
| Lai (2021) | ? | ? | ? | Y | ? | ? | ? | Y | Y | ? | 3 |
| Yang (2021) | ? | ? | ? | ? | ? | ? | ? | Y | Y | ? | 2 |
| Yang (2015) | ? | ? | ? | Y | ? | ? | ? | Y | Y | ? | 3 |
| Chen (2020) | ? | ? | ? | ? | ? | ? | ? | Y | Y | ? | 2 |
| Li (2015) | ? | ? | ? | ? | ? | ? | ? | Y | Y | ? | 2 |
| Yan (2020) | ? | ? | ? | ? | ? | ? | ? | Y | Y | ? | 2 |
| Tian (2017) | ? | ? | ? | ? | ? | ? | ? | Y | Y | ? | 2 |
| Yao (2016) | ? | ? | ? | ? | ? | ? | ? | Y | Y | ? | 2 |
| Jiang (2021) | ? | ? | ? | Y | ? | ? | ? | Y | Y | ? | 3 |
| Liu (2014) | ? | ? | ? | ? | ? | ? | ? | Y | Y | ? | 2 |
| Lin (2020) | ? | ? | ? | Y | ? | ? | ? | Y | Y | ? | 3 |
| Zhang (2021) | ? | ? | ? | ? | ? | ? | ? | Y | Y | ? | 2 |
| Zhang (2015) | ? | ? | ? | Y | ? | ? | ? | Y | Y | ? | 3 |
| Wang (2008) | ? | ? | ? | ? | ? | ? | ? | Y | Y | ? | 2 |
| Tiao (2014) | ? | ? | ? | ? | ? | ? | ? | Y | Y | ? | 2 |
| Wang (2022) | ? | ? | ? | Y | ? | ? | ? | Y | Y | ? | 2 |
| Wu (2021) | ? | ? | ? | Y | ? | ? | ? | Y | Y | ? | 3 |

Note: Selection bias: 1), Was the allocation sequence adequately generated and applied; 2), Were the groups similar at baseline or were they adjusted for confounders in the analysis; 3), Was the allocation adequately concealed. Performance bias: 4), Were the animals randomly housed during the experiment; 5), Were the caregivers and/or investigators blinded from knowledge which intervention each animal received during the experiment. Detection bias: 6), Were animals selected at random for outcome assessment; 7), Was the outcome assessor blinded. Attrition bias: 8), Were incomplete outcome data adequately addressed. Reporting bias: 9), Are reports of the study free of selective outcome reporting; Other bias: 10), Was the study apparently free of other problems that could result in high risk of bias. ?, unclear; Y, low risk; N, high risk.

**Table S2.** The results of subgroup analysis of the effect of ginsenoside Rg1 on MDA.

| **Indicators** | **Subgroup** |  | **No. of studies** | **SMD [95% CI]** | **P value** | **I^2^** | **p value for heterogeneity** |
| --- | --- | --- | --- | --- | --- | --- | --- |
| **MDA** | **Species** | rats | 1 | —— | —— | —— | —— |
|  |  | mice | 2 | -3.918 [-6.261 to -1.574] | 0.001 | 73.9% | 0.050 |
|  | **Model** | liver ischemia reperfusion injury | 2 | -2.993 [-7.215 to 1.229] | 0.000 | 42.1% | 0.159 |
|  |  | chemical liver injury | 1 | —— | —— | —— | —— |
|  | **Administration** | by tail vein injection | 1 | —— | —— | —— | —— |
|  |  | by intraperitoneal injection | 2 | -3.918 [-6.261 to -1.574] | 0.001 | 73.9% | 0.050 |
|  | **Duration** | ≤7 days | 1 | —— | —— | —— | —— |
|  |  | once | 2 | -2.993 [-7.215 to 1.229] | 0.000 | 42.1% | 0.159 |

**Table S3.** The results of subgroup analysis of the effect of ginsenoside Rg1 on TNF-α.

| **Indicators** | **Subgroup** |  | **No. of studies** | **SMD [95% CI]** | **P value** | **I^2^** | **p value for heterogeneity** |
| --- | --- | --- | --- | --- | --- | --- | --- |
| **TNF-α** | **Administration** | by intragastric | 1 | —— | —— | —— | —— |
|  |  | by intraperitoneal injection | 2 | -5.200 [-11.384 to 0.984] | 0.099 | 92.9% | 0.000 |
|  | **Duration** | ≤7 days | 2 | -5.834 [-10.768 to -0.901] | 0.020 | 87.4% | 0.005 |
|  |  | once | 1 | —— | —— | —— | —— |

**Table S4.** The results of subgroup analysis of the effect of ginsenoside CK on SOD.

| **Indicators** | **Subgroup** |  | **No. of studies** | **SMD [95% CI]** | **P value** | **I^2^** | **p value for heterogeneity** |
| --- | --- | --- | --- | --- | --- | --- | --- |
| **SOD** | **Species** | rat | 3 | 9.869 [2.207 to 17.532] | 0.012 | 95.5% | 0.000 |
|  |  | mice | 1 | —— | —— | —— | —— |
|  | **Prophylactic or Therapeutic** | therapeutic | 2 | 14.405 [-12.237 to 41.047] | 0.289 | 93.8% | 0.000 |
|  |  | prophylactic | 2 | 4.093 [-1.389 to 9.574] | 0.143 | —— | —— |
|  | **Duration** | ≥30 days | 1 | —— | —— | —— | —— |
|  |  | ＞7 days and ＜ 30 days | 3 | 2.797 [0.635 to 4.958] | 0.011 | 88.5% | 0.000 |

**Table S5.** The results of subgroup analysis of the effect of ginsenoside CK on MDA.

| **Indicators** | **Subgroup** |  | **No. of studies** | **SMD [95% CI]** | **P value** | **I^2^** | **p value for heterogeneity** |
| --- | --- | --- | --- | --- | --- | --- | --- |
| **MDA** | **Species** | rat | 3 | -3.102 [-8.015 to 2.028] | 0.040 |  |  |
|  |  | mice | 1 | —— | —— | —— | —— |
|  | **Prophylactic or Therapeutic** | therapeutic | 2 | -2.994 [-8.015 to 2.028] | 0.243 | 94.7% | 0.000 |
|  |  | prophylactic | 2 | -4.233 [-5.834 to -2.633] | 0.000 | 47.9% | 0.166 |
|  | **Duration** | ≥30 days | 1 | —— | —— | —— | —— |
|  |  | ＞7 days and ＜ 30 days | 3 | -2.970 [-5.803 to -0.136] | 0.04 | 92.3% | 0.000 |


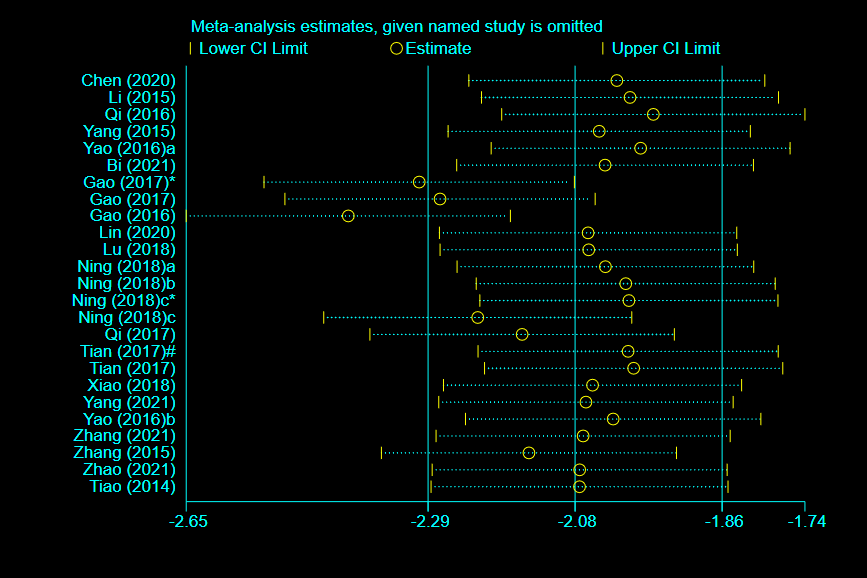


**Figure S1.** The results of the pooled effect values for ALT after excluding each trial of ginsenoside Rg1 in turn.


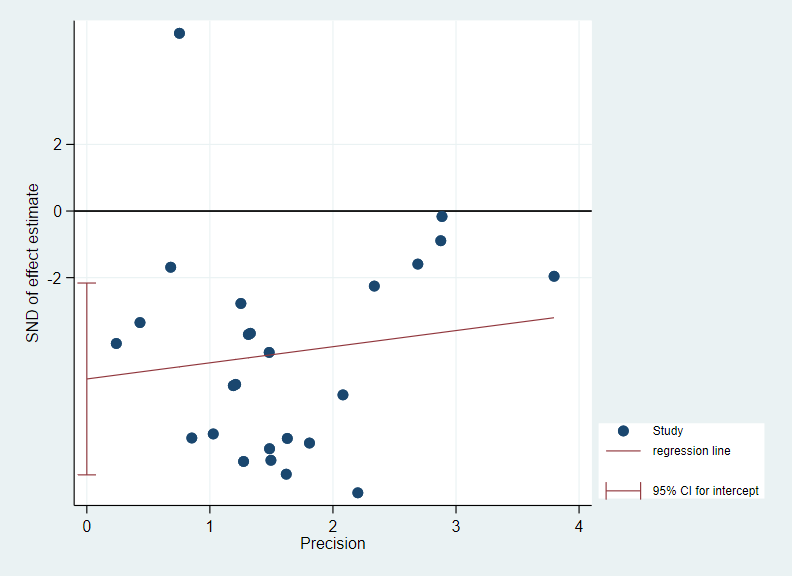


**Figure S2.** Egger’s publication bias plot for ALT ginsenoside Rg1.


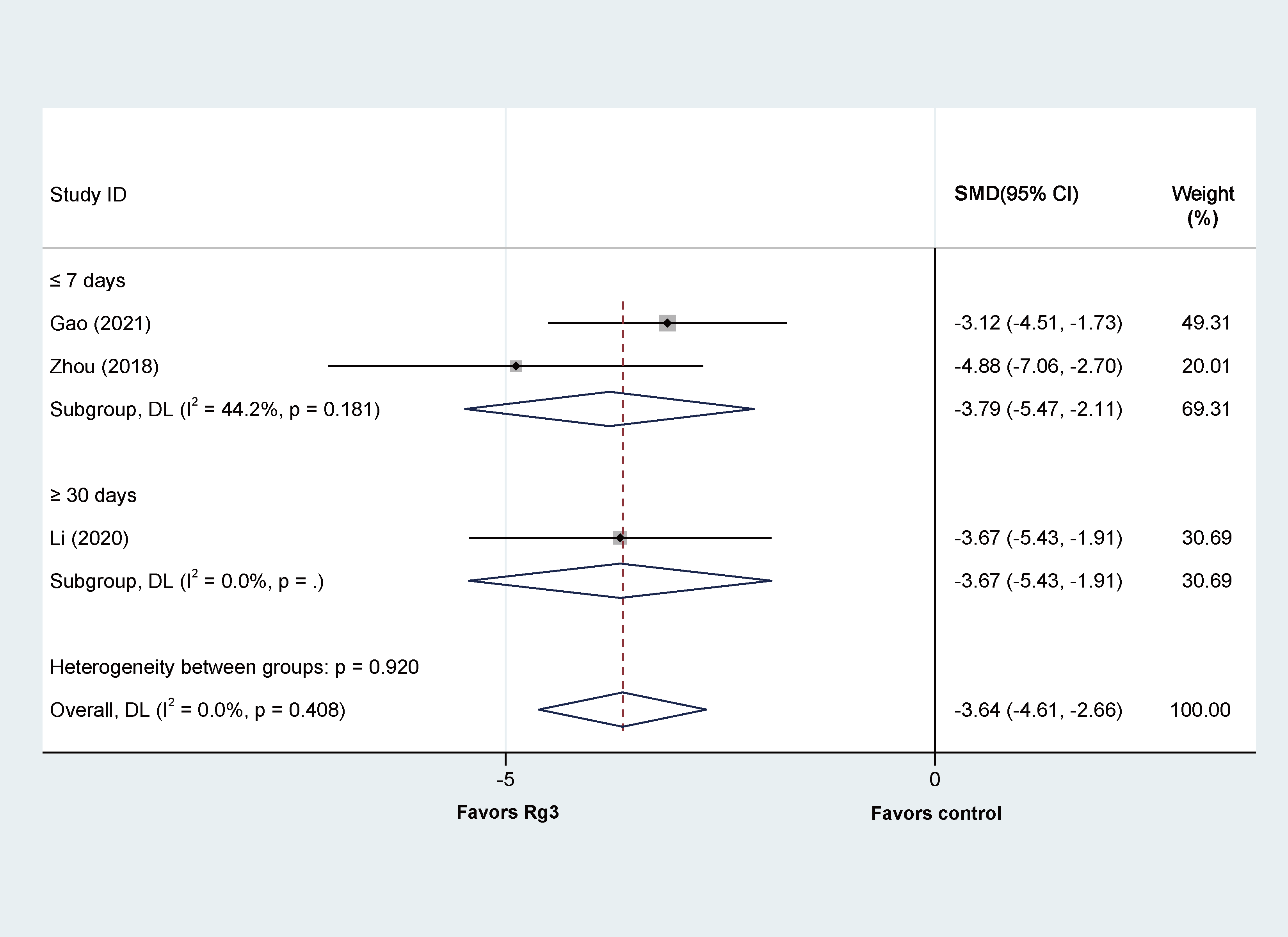


**Figure S3.** The results of subgroup analysis of the effect of ginsenoside Rg3 on MDA.


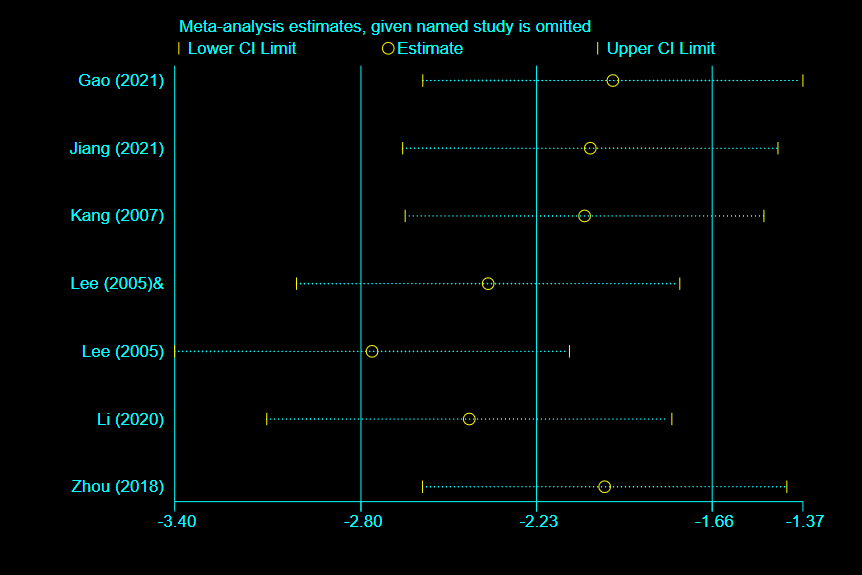


**Figure S4.** The results of the pooled effect values for ALT after excluding each trial of ginsenoside Rg3 in turn.


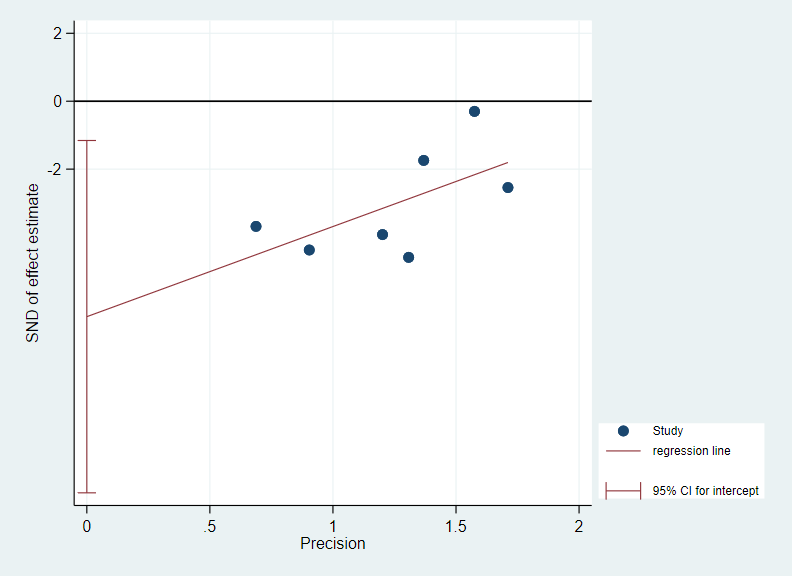


**Figure S5.** Egger’s publication bias plot for ginsenoside Rg3 on ALT.


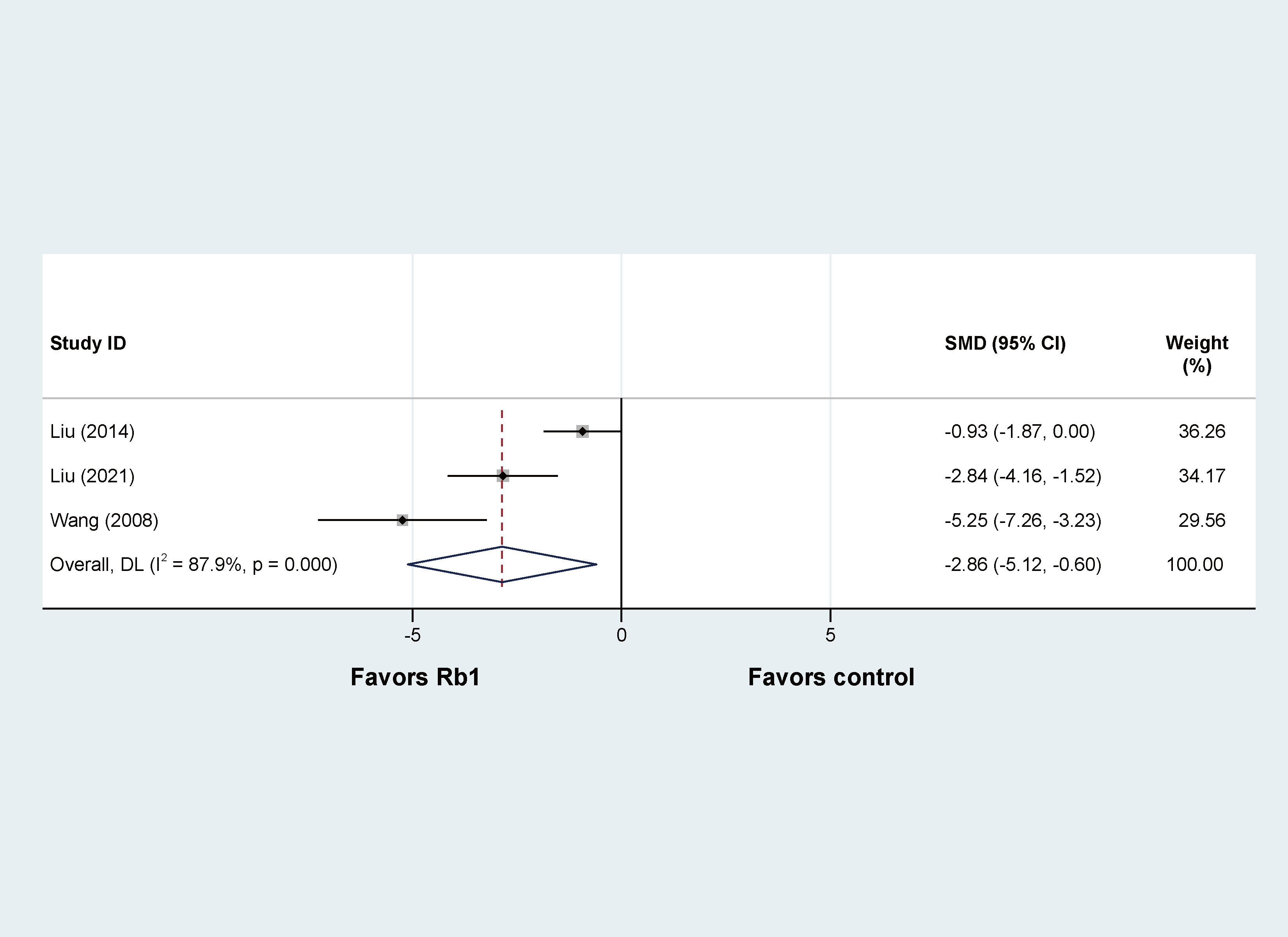


**Figure S6.** Standard mean differences estimates for the effects of ginsenoside Rb1 on MDA.


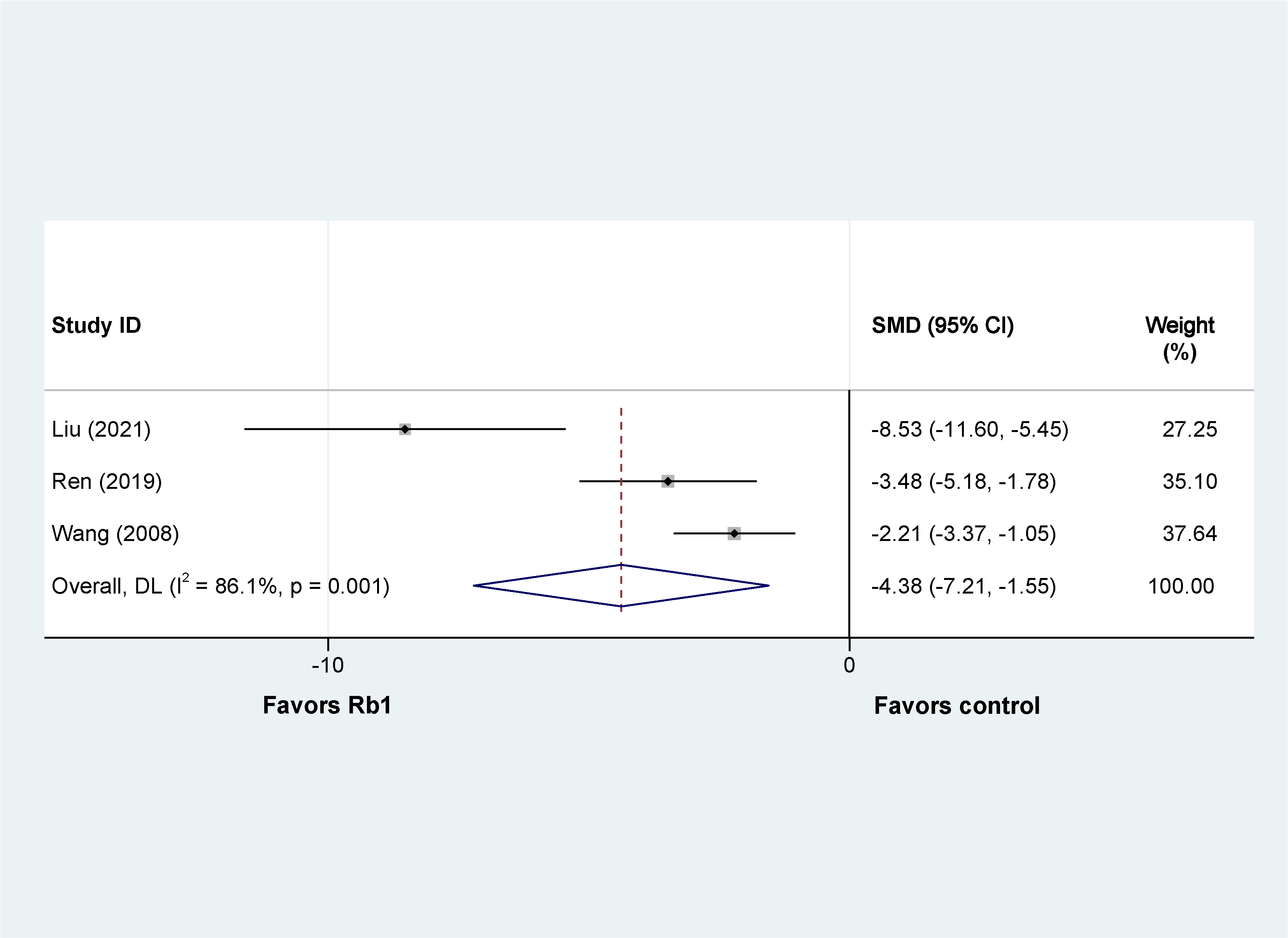


**Figure S7.** Standard mean differences estimates for the effects of ginsenoside Rb1 on TNF-α.


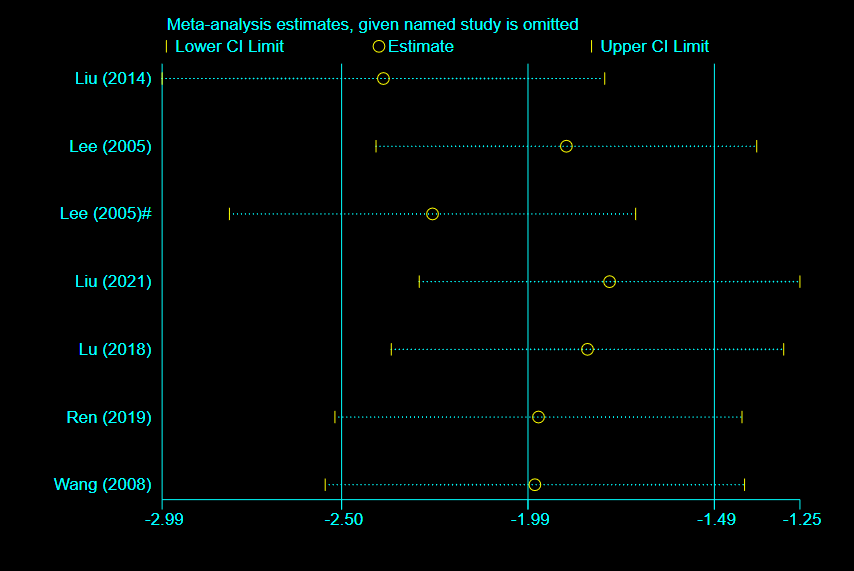


**Figure S8.** The results of the pooled effect values for ALT after excluding each trial of ginsenoside Rb1 in turn.


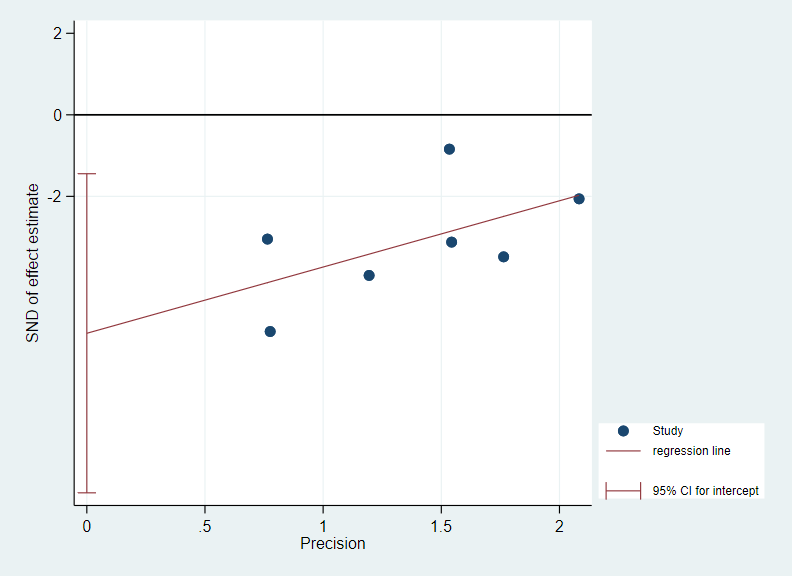


**Figure S9.** Egger’s publication bias plot for ginsenoside Rb1 on ALT.


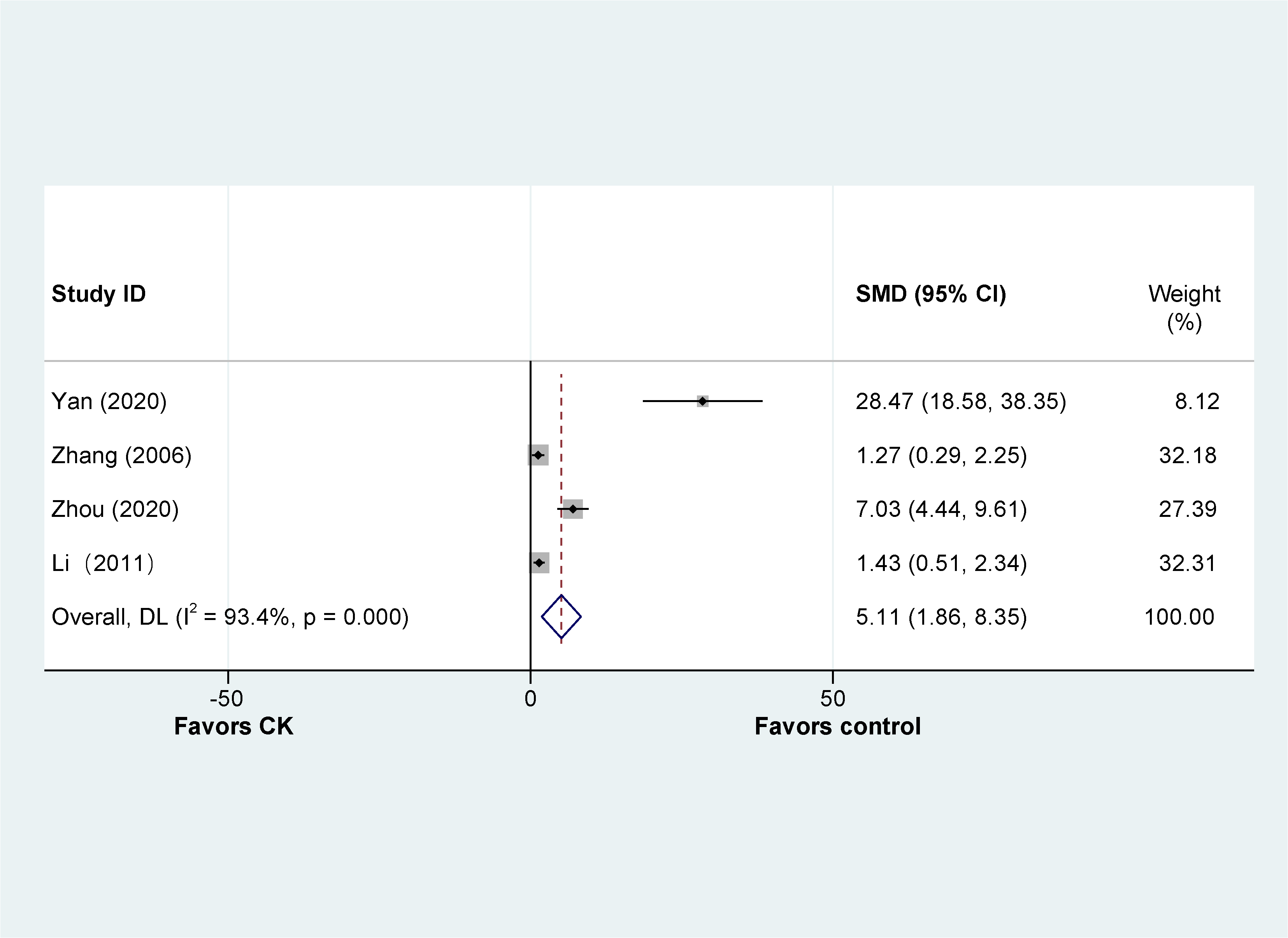


**Figure S10.** Standard mean differences estimates for the effects of ginsenoside CK on SOD.


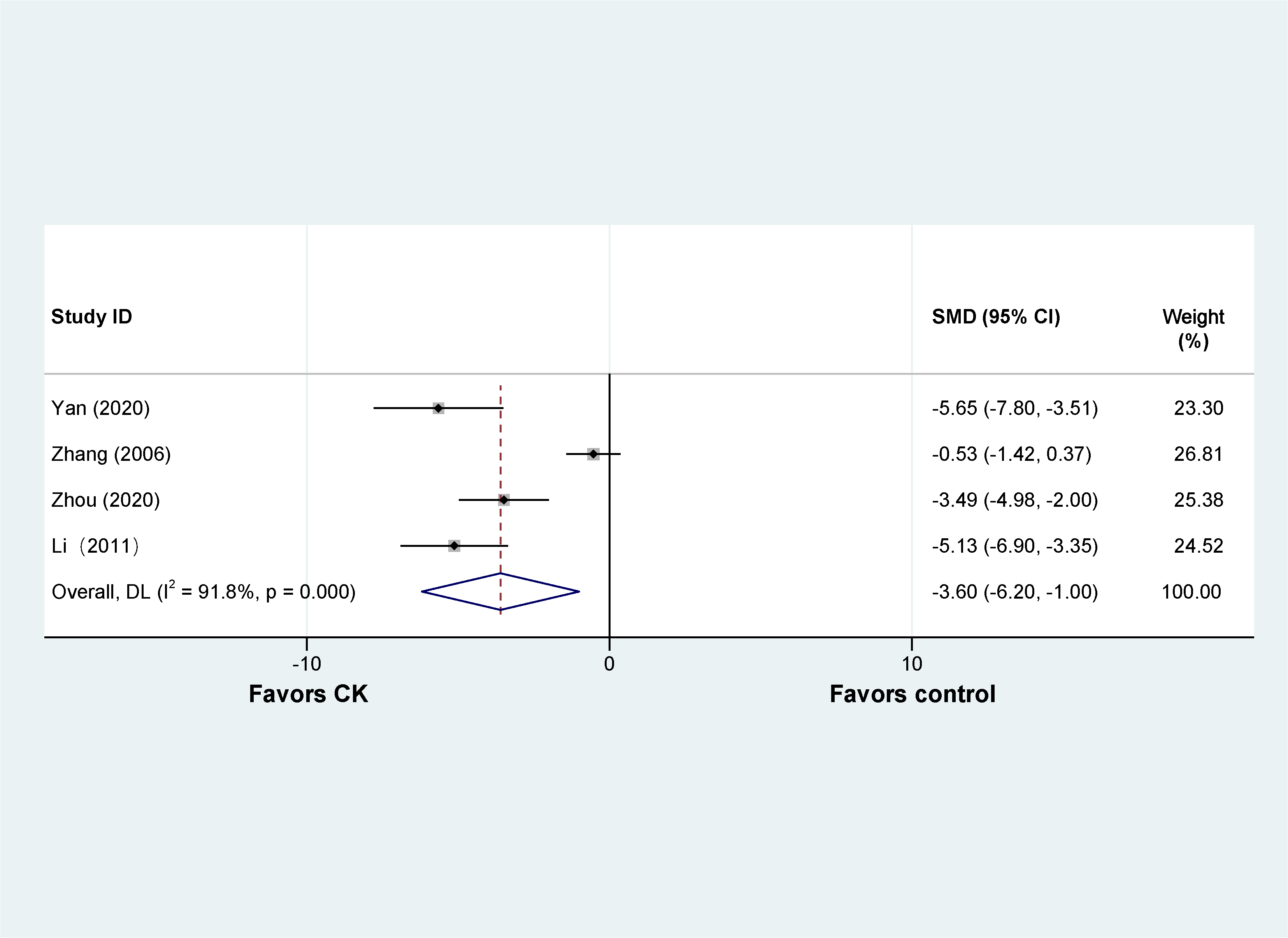


**Figure S11.** Standard mean differences estimates for the effects of ginsenoside CK on MDA.

**
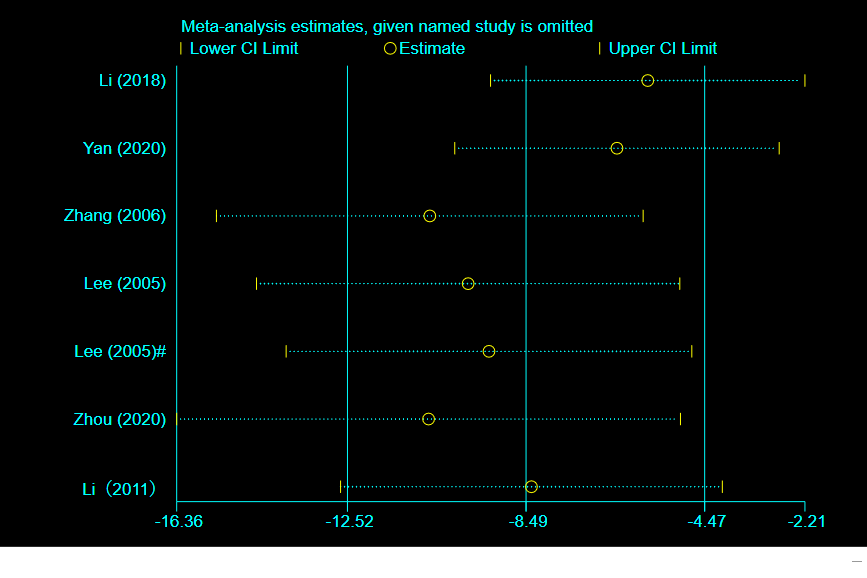
**

**Figure S12.** The results of the pooled effect values for ALT after excluding each trial of ginsenoside CK in turn.


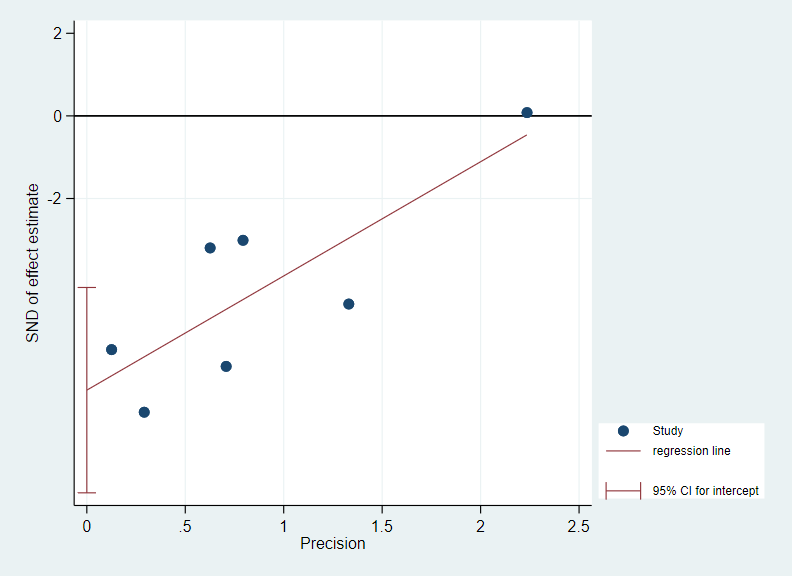


**Figure S13.** Egger’s publication bias plot for ginsenoside CK on ALT.
